# Homotrinuclear ruthenium(ii) and rhodium(i) complexes of redox-active tris(ferrocenyl)arene-based tris-phosphanes†

**DOI:** 10.1039/d4ra03822c

**Published:** 2024-08-06

**Authors:** Axel Straube, Peter Coburger, Evamarie Hey-Hawkins

**Affiliations:** a Institute of Inorganic Chemistry, Leipzig University Johannisallee 29 D-04103 Leipzig Germany hey@uni-leipzig.de

## Abstract

Homotrinuclear complexes of the *C*_3_-symmetric tris(ferrocenyl)arene-based tris-phosphanes 1a–d with ruthenium(ii) ([1a–d(Ru)_3_]) and rhodium(i) ([1a–d(Rh)_3_]) were prepared and fully characterised. Complexes [1a–d(Ru)_3_] and [1a–d(Rh)_3_] are electrochemically active. The nature of the arene core in 1a–d ranging from benzene, 1,3,5-trifluorobenzene and mesitylene to *s*-triazine allows to fine-tune the exact oxidation potentials for tailoring the electrochemical response. With a BAr^F^_4_^−^-based supporting electrolyte, a distinct separation of the three iron-centred oxidations of the ligand backbone is observable. Under these conditions, these oxidations are mostly reversible but, especially for the third oxidation, already show signs of irreversibility. In general, while the coordinated metal complex fragment does not strongly alter the electrochemical response of the arene-trisferrocenyl core 1a–d, there are observable differences. Rhodium(i) complexes are oxidised at slightly higher potentials than ruthenium(ii) complexes. In both cases, individual oxidation states for the C_6_H_3_(CH_2_)_3_-based ligand (1d) are difficult to address and the C_3_N_3_-based ligand (1c) shows the most complicated and least reversible electrochemistry with severely broadened third oxidations and reduced reversibility in cyclic voltammetry. The most well-suited system for potential applications in redox-switchable catalysis, in all cases, is the C_6_H_3_-based ligand (1a), showing entirely reversible and well-separated redox events.

## Abstract 2

*C*_3_-symmetric homotrinuclear Rh^I^ and Ru^II^ complexes with four accessible oxidation states relating to the tris(ferrocenyl)arene backbone were prepared, making these complexes potentially suitable for redox-switchable catalysis or other applications.

## Graphical abstract

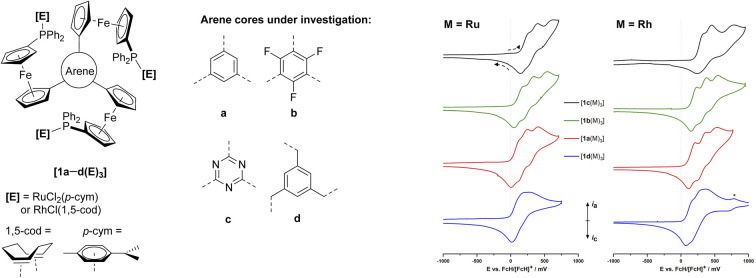

## Introduction

**Scheme 1:**
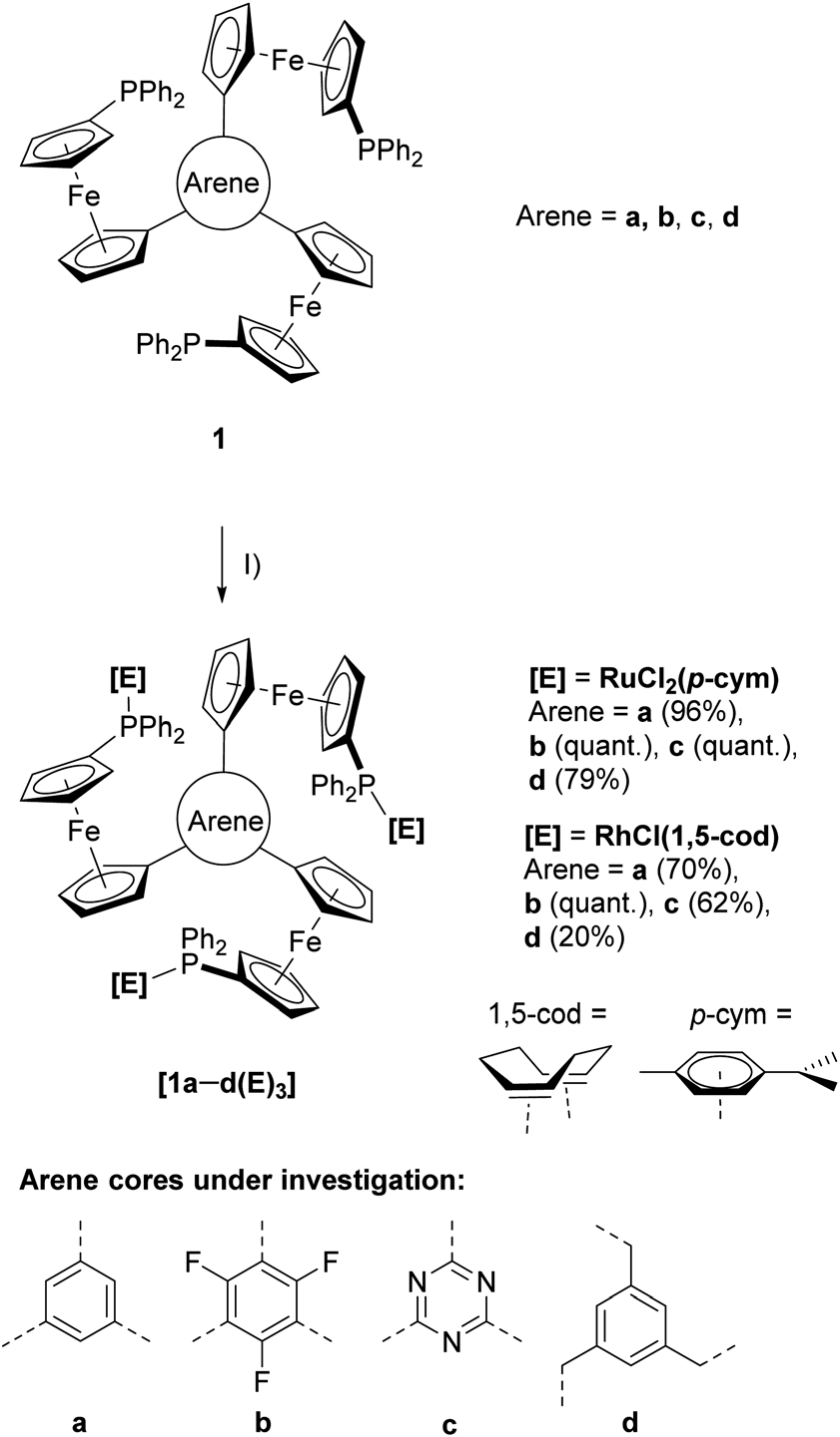
Preparation of ruthenium(ii) ([1a–d(Ru)_3_]) and rhodium(i) ([1a–d(Rh)_3_]) complexes from tris-phosphanes 1a–d and (I) [{RuCl_2_(*p*-cym)}_2_] or [{RhCl(1,5-cod)}_2_] in CH_2_Cl_2_ at r.t.

The last decades have seen *C*_3_-symmetry increasingly being adopted into ligand design due to enhanced stability and outstanding performances in asymmetric catalysis due to a reduced possible number of transition states.^1,2^ Next to countless nitrogen-containing compounds,^3–6^ several tris-phosphanes have been reported,^7–11^ often based on the archetypical triphos ligand by Hewertson and Watson.^12^ The corresponding *C*_3_-symmetric ligands featuring three ferrocenylene groups were reported by Butler and co-workers in 2003.^13^ Ferrocene is highly suitable for ligand design, owing to its amenability to synthetic modification and favourable, while modifiable, redox properties.^14,15^ Constructing multi-ferrocene systems is usually motivated by the redox properties of the individual ferrocene moieties which, when assembled, can add up to more than the mere sum of its parts.^16,17^ Motivated by the prominence of *C*_3_-symmetry in modern-day ligand design and the potential to exploit the use of three ferrocenyl groups for redox-switchable catalysis (RSC), we have built upon our previous works on ferrocenylphosphanes^18–26^ and their applications in RSC^27–29^ and reported a new family of tris-phosphanes (1a–d, Scheme 1) based on a redox-active, *C*_3_-symmetric tris(ferrocenyl)arene backbone. By incorporating an electron-withdrawing (b and c) or a tris-benzylic arene core (d), the electrochemical response as well as more subtle influences on the coordination behaviour of the corresponding phosphanes become adjustable. Thus, tris-phosphanes 1a–d were employed in the formation of mono- and trinuclear gold(i) complexes, and the latter were shown to act as four-state redox-switchable catalysts.^30–32^ We have now extended this synthetic concept to homotrinuclear rhodium(i) and ruthenium(ii) complexes.

## Experimental general procedures

### Syntheses

All reactions and manipulations were carried out under an atmosphere of either nitrogen or argon using standard Schlenk line techniques unless stated otherwise. Thin-layer chromatography (TLC) with silica gel 60 F_254_ on glass or aluminium sheets available from Merck KGaA was used for monitoring the ligand synthesis. Column chromatography of the ruthenium complexes was performed using silica gel (Macherey-Nagel 60, 0.04–0.063 mm) and dried solvents purged with nitrogen prior to use. Molecular sieves (4 Å) were activated at 300 °C *in vacuo* for a minimum of 3 h. Dry, oxygen-free solvents (THF, CH_2_Cl_2_, Et_2_O, hexanes, and toluene) were obtained from an MBraun Solvent Purification System MB SPS-800 and directly stored over 4 Å molecular sieves, except for THF, which was further distilled from potassium/benzophenone and stored over 4 Å molecular sieves. CD_2_Cl_2_ was dried by stirring over P_2_O_5_ at room temperature for several days, followed by vacuum transfer into a storage flask, degassing by the freeze–pump–thaw method, and storage over 4 Å molecular sieves. THF-d_8_ was distilled from potassium/benzophenone and stored over 4 Å molecular sieves after degassing by the freeze–pump–thaw method. Tris-phosphanes 1a,^30^1b,^30^1c,^31^ and 1d,^30^ (*n*Bu_4_N)[B{3,5-C_6_H_3_(CF_3_)_2_}_4_] (=(*n*Bu_4_N)BAr^F^_4_),^33^ and the transition metal precursors [{Rh(μ-Cl)(1,5-cod)}_2_]^34^ (1,5-cod = η^4^-cycloocta-1,5-diene) and [{RuCl(μ-Cl)(*p*-cym)}_2_]^35^ (*p*-cym = η^6^-*p*-cymene) were prepared according to previously published procedures. All other chemicals were used as purchased.

NMR spectra were recorded with a BRUKER Avance III HD 400 MHz NMR spectrometer at 25 °C (frequencies of ^1^H: 400.13 MHz; ^13^C 100.63 MHz; ^19^F: 376.53 MHz; ^31^P: 161.99 MHz). Pseudo-triplets and -quadruplets (due to additional coupling to heteronuclei like ^19^F and ^31^P) of ferrocenyl protons are abbreviated as pt/pq and their observable coupling constants *J* are given. Quintuplets are abbreviated as “quint”. Assignment of ^1^H and ^13^C signals to the respective chemical entities are based on ^1^H,^1^H COSY, phase-sensitive ^1^H,^13^C HSQC and ^1^H,^13^C HMBC NMR experiments. TMS was used as the internal standard in the ^1^H and ^13^C{^1^H}/^13^C{^31^P,^1^H} NMR spectra, and spectra of all other nuclei were referenced to TMS using the Ξ scale.^36^ The numbering schemes for the assignment of specific nuclei is given in the ESI.†

Electrospray ionisation (ESI) mass spectrometry was performed with an ESI ESQUIRE 3000 PLUS spectrometer with an IonTrap analyser from Bruker Daltonics, or a MicroTOF spectrometer from Bruker Daltonics with a ToF analyser in positive mode. As solvents for the measurements, pure degassed CH_2_Cl_2_ or mixtures of degassed CH_2_Cl_2_ and CH_3_CN were used. Elemental analyses were performed with a VARIO EL elemental analyser from Heraeus. Melting points were determined with a Gallenkamp MPD350 BM2.5 melting point device and are reported uncorrected. FTIR spectra were obtained with a PerkinElmer FT-IR spectrometer Spectrum 2000 as KBr pellets and with a Thermo Scientific Nicolet iS5 with an ATR unit in the range from 4000 to 400 cm^−1^.

### Crystallography

The data were collected on a Gemini-CCD diffractometer (RIGAKU INC.) using Mo-K_α_ radiation (*λ* = 0.71073 Å), ω-scan rotation. Data reduction was performed with CrysAlis Pro^37^ including the program SCALE3 ABSPACK^38^ for empirical absorption correction. The structure solution for [1a(Rh)_3_] was performed with SHELXS-97 (direct methods).^39^ The anisotropic full-matrix least-squares refinement on *F*^2^ of all non-hydrogen atoms was performed with SHELXL-97.^40^ All non-hydrogen atoms were refined with anisotropic thermal parameters. The structure figures were generated with Mercury (versions 3.8 and 3.10)^40^ and POV-Ray (Version 3.7).^41^ CCDC 1990280 contains the supplementary crystallographic data for this paper. These data are provided free of charge by The Cambridge Crystallographic Data Centre.

### Electrochemistry

Cyclic voltammetry (CV) measurements on 1.0 mmol per L analyte solutions in dry, oxygen-free dichloromethane containing 0.1 mol per L (*n*Bu_4_N)BF_4_ or (*n*Bu_4_N)BAr^F^_4_ as supporting electrolyte were conducted in a three-electrode setup (GAMRY Instruments, SP-50 potentiostat by BioLogic Science Instruments) under a blanket of nitrogen at room temperature. The glassy-carbon working electrode (ALS; surface area 0.07 cm^2^) and the counter electrode (neoLab; platinum wire, 99.9%) were immersed in the analyte solution, while the reference electrode (ALS; Ag/AgNO_3_ (0.01 mol L^−1^) in 0.1 mol per L tetrabutylammonium hexafluorophosphate in dry, oxygen-free CH_3_CN) was connected to the cell *via* a bridge tube (filled with the supporting electrolyte) through Vycor tips. The reference electrode was calibrated against decamethylferrocene as an internal standard at the end of the CV experiment,^42^ and the results were converted to the FcH/[FcH]^+^ scale in accordance with the IUPAC requirements.^43^

### Synthesis of μ_3_-[1,3,5-tris(1-diphenylphosphanyl-1′-ferrocenylene)benzene-1κ^1^*P*,2κ^1^*P*,3κ^1^*P*]-tris[dichlorido-η^6^-(*p*-cymene)ruthenium(ii)] ([1a(Ru)_3_])

In a Schlenk flask, 1a (100 mg, 84.7 μmol, 1.00 eq.) and [{RuCl(μ-Cl)(*p*-cym)}_2_] (77.7 mg, 127 μmol, 1.50 eq.) were dissolved in CH_2_Cl_2_ (10 mL) and stirred overnight at room temperature. The reaction mixture was concentrated to half the original volume and filtered over degassed silica, using THF to elute the product. Complex [1a(Ru)_3_] was obtained as a red microcrystalline solid (170 mg, 96%) after removal of the volatiles *in vacuo*.

M.p.: 210 °C (decomposition; from THF); ^1^H NMR (CD_2_Cl_2_): *δ* [ppm] = 7.91–7.84 (m, 12H, H10), 7.51–7.37 (m, 18H, H11 + 12), 7.17 (s, 3H, H2), 5.08 (m, 12H, H14 + 15), 4.43 (pq, *J* = 1.7 Hz, 6H, H8), 4.38 (pt, *J*_H,H_ = 1.9 Hz, 6H, H4/5), 4.14 (pq, *J* = 1.7 Hz, 6H, H7), 3.83 (pt, *J*_H,H_ = 1.9 Hz, 6H, H5/4), 2.46 (sept,^3^*J*_H,H_ = 6.9 Hz, 3H, H18), 1.76 (s, 9H, H17), 0.94 (d, ^3^*J*_H,H_ = 6.9 Hz, 18H, H19); ^13^C{^1^H} NMR (CD_2_Cl_2_): *δ* [ppm] = 138.2 (s, C1), 136.2 (d, ^1^*J*_C,P_ = 47.3 Hz, C9), 134.2 (d, ^2^*J*_C,P_ = 9.3 Hz, C10), 130.1 (d, ^4^*J*_C,P_ = 2.4 Hz, C12), 127.5 (d, ^3^*J*_C,P_ = 9.8 Hz, C11), 122.2 (s, C2), 108.8 (d, ^2^*J*_C,P_ = 1.0 Hz, C13), 95.2 (s, C16), 90.1 (d, ^2^*J*_C,P_ = 4.4 Hz, C15), 86.2 (s, C3), 86.1 (d, ^2^*J*_C,P_ = 6.0 Hz, C14), 78.1 (d, ^1^*J*_C,P_ = 47.5 Hz, C6), 75.6 (d, ^3^*J*_C,P_ = 10.5 Hz, C8), 73.4 (d, ^2^*J*_C,P_ = 7.9 Hz, C7), 72.4 (s, C4/5), 67.9 (s, C5/4), 30.1 (s, C18), 21.6 (s, C19), 17.1 (s, C17); ^31^P{^1^H} NMR (CD_2_Cl_2_): *δ* [ppm] = 18.9 (s); IR (neat): *ν̃* [cm^−1^] = 3077 (w), 3052 (w), 2957 (m), 2923 (m), 2867 (m, all *ν*(C–H)), 1594 (w), 1559 (w), 1540 (w), 1507 (w), 1498 (w), 1481 (w), 1472 (w), 1457 (w), 1432 (m, *ν*(C–P)), 1381 (w), 1361 (w), 1318 (w), 1306 (w), 1198 (w), 1188 (w), 1157 (m), 1094 (m), 1058 (m), 1027 (m), 999 (w), 921 (w), 888 (w), 827 (m), 799 (m), 744 (m), 695 (s), 669 (m), 623 (m), 562 (w), 540 (m), 519 (m), 509 (m), 492 (s), 471 (s), 414 (m); HRMS (ESI): *m*/*z* calcd for C_102_H_99_Cl_4_Fe_3_P_3_Ru_3_ 1015.5458 [M − 2Cl]^2+^; found 1015.5474; elemental analysis calcd [%] for C_102_H_99_Cl_6_Fe_3_P_3_Ru_3_: C 58.30, H 4.75, found: C 55.95, H 4.72.

### Synthesis of μ_3_-[2,4,6-tris(1-diphenylphosphanyl-1′-ferrocenylene)-1,3,5-trifluorobenzene-1κ^1^*P*,2κ^1^*P*,3κ^1^*P*]-tris[dichlorido-η^6^-(*p*-cymene)ruthenium(ii)] ([1b(Ru)_3_])

[1b(Ru)_3_] was prepared analogously to [1a(Ru)_3_] using 1b (50.0 mg, 40.4 μmol, 1.00 eq.) and obtained as a red amorphous solid in quantitative yield (87.0 mg).

M.p.: 188 °C (decomposition; from THF); ^1^H NMR (CD_2_Cl_2_): *δ* [ppm] = 7.99–7.71 (m, 12H, H10), 7.54–7.28 (m, 18H, H11 + 12), 5.09 (pq, *J* = 6.2 Hz, 12H, H14 + 15), 4.44 (m, 6H, H8), 4.37 (m, 6H, H4), 4.31 (m, 6H, H7), 3.96 (pt, *J*_H,H_ = 1.9 Hz, 6H, H5), 2.45 (hept, ^3^*J*_H,H_ = 7.0 Hz, 3H, H18), 1.76 (s, 9H, H17), 0.94 (d, ^3^*J*_H,H_ = 7.0 Hz, 18H, H19); ^13^C{^1^H} NMR (CD_2_Cl_2_):^44^*δ* [ppm] = 155.2 (s, C1), 135.8 (d, ^1^*J*_C,P_ = 47.3 Hz, C9), 134.1 (d, ^2^*J*_C,P_ = 9.4 Hz, C10), 130.1 (d, ^4^*J*_C,P_ = 1.9 Hz, C12), 127.4 (d, ^3^*J*_C,P_ = 9.7 Hz, C11), 111.5 (s, C2), 108.6 (d, ^2^*J*_C,P_ = 0.7 Hz, C13), 95.0 (s, C16), 90.2 (d, ^2^*J*_C,P_ = 4.4 Hz, C15), 85.9 (d, ^2^*J*_C,P_ = 5.8 Hz, C14), 78.1 (d, ^1^*J*_C,P_ = 47.7 Hz, C6), 75.7 (d, ^3^*J*_C,P_ = 10.2 Hz, C8), 74.6 (s, C3), 72.9 (d, ^2^*J*_C,P_ = 7.9 Hz, C7), 71.7 (s, C5), 71.2 (m, C4), 30.0 (s, C18), 21.5 (s, C19), 17.0 (s, C17); ^19^F{^1^H} (CD_2_Cl_2_): *δ* [ppm] = −107.5 (s); ^31^P{^1^H} (CD_2_Cl_2_): *δ* [ppm] = 19.4 (s); IR (neat): *ν̃* [cm^−1^] = 3077 (w), 3052 (w), 2957 (m), 2923 (m), 2867 (m, all *ν*(C–H)), 1594 (w), 1559 (w), 1540 (w), 1507 (w), 1498 (w), 1483 (m), 1472 (w), 1457 (w), 1432 (m, *ν*(C–P)), 1421 (m, *ν*(C–F)), 1387 (m), 1361 (w), 1318 (w), 1308 (m), 1229 (m), 1198 (w), 1188 (w), 1157 (m), 1094 (m), 1058 (m), 1027 (m), 999 (w), 921 (w), 888 (w), 827 (m), 799 (m), 744 (m), 695 (s), 669 (m), 623 (m), 562 (w), 540 (m), 520 (m), 509 (m), 492 (s), 469 (*vs.*), 454 (s), 414 (m), 405 (s); HRMS (ESI): *m*/*z* calcd for C_102_H_96_Cl_6_F_3_Fe_3_P_3_Ru_3_ [M]^+^ 2156.0003, calcd for C_102_H_96_Cl_5_F_3_Fe_3_P_3_Ru_3_ [M − Cl]^+^ 2120.0323; found 2156.0060, 2120.0362; elemental analysis calcd [%] for C_102_H_96_Cl_6_F_3_Fe_3_P_3_Ru_3_: C 56.84, H 4.49, found: C 56.43, H 4.11.

### Synthesis of μ_3_-[2,4,6-tris(1-diphenylphosphanyl-1′-ferrocenylene)-1,3,5-triazine-1κ^1^*P*,2κ^1^*P*,3κ^1^*P*]-tris[dichlorido-η^6^-(*p*-cymene)-ruthenium(ii)] ([1c(Ru)_3_])

[1c(Ru)_3_] was prepared analogously to [1a(Ru)_3_] using 1c (50.0 mg, 42.2 μmol, 1.00 eq.) and obtained as a dark-red amorphous solid in quantitative yield (84.9 mg).

M.p.: 175 °C (decomposition; from THF); ^1^H NMR (CD_2_Cl_2_): *δ* [ppm] = 7.94–7.84 (m, 12H, H9), 7.58–7.34 (m, 18H, H10 + 11), 5.10 (pq, *J* = 6.0 Hz, 12H, H13 + 14), 4.84 (m, 6H, H3), 4.51 (m, 6H, H7), 4.07 (m, 6H, H6), 3.99 (m, 6H, H4), 2.46 (hept, ^3^*J*_H,H_ = 6.9 Hz, 3H, H17), 1.78 (s, 9H, H16), 0.93 (d, ^3^*J*_H,H_ = 6.9 Hz, 18H, H18); ^13^C{^1^H} NMR (CD_2_Cl_2_): *δ* [ppm] = 175.0 (s, C1), 136.1 (d, ^1^*J*_C,P_ = 47.3 Hz, C8), 134.1 (d, ^2^*J*_C,P_ = 9.4 Hz, C9), 130.2 (d, ^4^*J*_C,P_ = 2.4 Hz, C11), 127.5 (d, ^3^*J*_C,P_ = 9.7 Hz, C10), 108.7 (s, C12), 95.1 (s, C15), 90.2 (d, ^2^*J*_C,P_ = 4.4 Hz, C14), 86.0 (d, ^2^*J*_C,P_ = 6.0 Hz, C13), 80.4 (s, C2), 78.3 (d, ^1^*J*_C,P_ = 47.0 Hz, C5), 76.0 (d, ^3^*J*_C,P_ = 10.2 Hz, C7), 75.2 (s, C4), 73.3 (d, ^2^*J*_C,P_ = 7.7 Hz, C6), 70.7 (s, C3), 30.0 (s, C17), 21.5 (s, C18), 17.0 (s, C16); ^31^P{^1^H} (CD_2_Cl_2_): *δ* [ppm] = 18.8 (s); IR (neat): *ν̃* [cm^−1^] = 3077 (w), 3052 (w), 2957 (m), 2923 (m), 2867 (m, all *ν*(C–H)), 1506 (s), 1498 (w), 1483 (m), 1472 (w), 1457 (w), 1432 (m, *ν*(C–P)), 1380 (m), 1357 (m), 1319 (m), 1308 (m), 1229 (m), 1198 (w), 1188 (w), 1157 (m), 1094 (m), 1058 (m), 1028 (m), 999 (w), 925 (w), 888 (w), 827 (m), 799 (m), 744 (m), 695 (s), 669 (m), 623 (m), 562 (w), 540 (m), 509 (m), 492 (s), 469 (s), 454 (s), 424 (m), 409 (m); HRMS (ESI): *m*/*z* calcd for C_99_H_96_Cl_5_Fe_3_N_3_P_3_Ru_3_ [M − Cl]^+^ 2069.0462, calcd for C_101_H_99_Cl_5_Fe_3_N_4_P_3_Ru_3_ [M − Cl + CH_3_CN]^+^ 2110.0728; found 2069.0479, 2110.0723; elemental analysis calcd [%] for C_99_H_96_Cl_6_Fe_3_N_3_P_3_Ru_3_: C 56.51, H 4.60, N 2.00, found: C 55.77, H 4.41, N 1.95.

### Synthesis of μ_3_-[1,3,5-tris{(1-diphenylphosphanyl-1′-ferrocenylene)methyl}benzene -1κ^1^*P*,2κ^1^*P*,3κ^1^*P*]-tris[dichlorido-η^6^-(*p*-cymene)-ruthenium(ii)] ([1d(Ru)_3_])

[1d(Ru)_3_] was prepared analogously to [1a(Ru)_3_] using 1d (50.0 mg, 40.8 μmol, 1.00 eq.) and obtained as a dark-red amorphous solid (69.0 mg, 79%).

M.p.: >160 °C decomp. (from THF/diethyl ether); ^1^H NMR (CD_2_Cl_2_): *δ* [ppm] = 7.92–7.81 (m, 12H, H11), 7.47–7.34 (m, 18H, H12 + H13), 6.48 (s, 3H, H1), 5.14–5.06 (m, 12H, H15 + H16), 4.37 (pq, *J*_H,H_ = 1.7 Hz, 6H, H9), 4.27 (pq, *J*_H,H_ = 1.7 Hz, 6H, H8), 3.69 (pt, *J*_H,H_ = 1.8 Hz, 6H, H5), 3.57 (pt, *J*_H,H_ = 1.8 Hz, 6H, H6), 3.23 (s, 6H, H3), 2.49 (hept, ^3^*J*_H,H_ = 7.0 Hz, 3H, H18), 1.79 (s, 9H, H20), 0.97 (d, ^3^*J*_H,H_ = 7.0 Hz, 18H, H19); ^13^C{^1^H} NMR (CD_2_Cl_2_): *δ* [ppm] = 141.3 (s, C2), 136.2 (d, ^1^*J*_C,P_ = 47.3 Hz, C10), 134.1 (d, ^2^*J*_C,P_ = 9.3 Hz, C11), 130.0 (d, ^4^*J*_C,P_ = 2.3 Hz, C13), 127.3 (d, ^3^*J*_C,P_ = 9.7 Hz, C12), 125.7 (s, C1), 108.6 (s, C14), 95.0 (s, C17), 90.1 (d, ^2^*J*_C,P_ = 4.4 Hz, C16), 89.3 (s, C4), 85.9 (d, ^2^*J*_C,P_ = 5.9 Hz, C15), 77.4 (d, ^1^*J*_C,P_ = 48.4 Hz, C7), 75.1 (d, ^3^*J*_C,P_ = 10.5 Hz, C9), 71.2 (d, ^2^*J*_C,P_ = 8.0 Hz, C8), 70.29 (s, C5/C6), 70.27 (s, C6/C5), 35.0 (s, C3), 30.0 (s, C18), 21.5 (s, C19), 17.0 (s, C20); ^31^P{^1^H} NMR (CD_2_Cl_2_): *δ* [ppm] = 18.8 (s); IR (neat, ATR): *ν̃* [cm^−1^] = 3077 (m), 3053 (m), 2959 (s), 2926 (s), 2867 (m, all *ν*(C–H)), 1599 (m), 1574 (w), 1537 (w), 1481 (m), 1468 (m), 1433 (s, *ν*(C–P)), 1385 (m), 1305 (m), 1236 (m), 1190 (m), 1157 (s), 1095 (m), 1058 (m), 1026 (s), 1000 (w), 981 (w), 925 (w), 890 (w), 827 (m), 800 (m), 745 (m), 697 (s), 670 (m), 541 (m), 520 (m), 491 (s), 471 (s), 457 (m), 437 (w), 423 (w); HRMS (ESI): *m*/*z* calcd for C_105_H_105_Cl_6_Fe_3_P_3_Ru_3_ [M]^+^ 2143.0760, for C_105_H_105_Cl_5_Fe_3_P_3_Ru_3_ [M − Cl]^+^ 2108.1077; found 2143.0737, 2108.1052; elemental analysis calcd [%] for C_105_H_105_Cl_6_Fe_3_P_3_Ru_3_: C 58.84, H 4.94, found: C 58.17, H 4.97.

### Synthesis of μ_3_-[1,3,5-tris(1-diphenylphosphanyl-1′-ferrocenylene)benzene-1κ^1^*P*,2κ^1^*P*,3κ^1^*P*]-tris[chlorido-1,2,5,6-η^4^-(cycloocta-1,5-diene)rhodium(i)] ([1a(Rh)_3_])

Under stirring, a solution of 1a (120 mg, 101 μmol, 1.00 eq.) in THF (10 mL) was added to [{Rh(μ-Cl)(1,5-cod)}_2_] (76.0 mg, 154 μmol, 1.52 eq.) in THF (5 mL) and kept stirring at room temperature overnight. Complex [1a(Rh)_3_] was precipitated from the clear orange solution using diethyl ether (45 mL), yielding a fine orange powder which was dried *in vacuo* at 40 °C (136 mg, 70%). Crystals suitable for XRD were obtained from vapour diffusion of diethyl ether into 0.7 mL of the reaction mixture in an NMR tube at room temperature.

M.p.: >185 °C decomp. (from THF/diethyl ether); ^1^H NMR (THF-d_8_): *δ* [ppm] = 7.65 (s, 3H, H2), 7.62–7.48 (m, 12H, H11), 7.39–7.14 (m, 18H, H10 + 12), 5.56 (m, 6H, H13/14/17/18), 5.10 (pt, *J*_H,H_ = 1.9 Hz, 6H, H4/5), 4.78 (m, 6H, H8), 4.55 (pt, *J*_H,H_ = 1.9 Hz, 6H, H5/4), 4.25 (m, 6H, H7), 3.03 (m, 6H, H14/13/18/17), 2.49–2.34 (m, 12H, H15 + 16,19 + 20), 2.07 (m, 6H, H15 + 16,19 + 20), 1.92 (m, 6H, H15 + 16,19 + 20); ^13^C{^1^H} NMR (THF-d_8_): *δ* [ppm] = 138.7 (s, C1), 133.7 (d, ^3^*J*_C,P_ = 11.0 Hz, C11), 133.6 (d, ^1^*J*_C,P_ = 42.5 Hz, C9), 129.2 (d, ^4^*J*_C,P_ = 1.9 Hz, C12), 127.1 (d, ^2^*J*_C,P_ = 9.5 Hz, C10), 122.1 (s, C2), 103.8 (dd, ^2^*J*_C,P_ = 13.5 Hz, ^1^*J*_C,Rh_ = 7.5 Hz, C13/14/17/18), 86.4 (s, C3), 75.7 (d, ^3^*J*_C,P_ = 10.5 Hz, C8), 73.5 (d, ^2^*J*_C,P_ = 6.9 Hz, C7), 73.4 (d, ^1^*J*_C,P_ = 47.5 Hz, C6), 72.0 (s, C4/5), 69.5 (d, ^1^*J*_C,Rh_ = 13.3 Hz, C14/13/18/17), 68.3 (s, C5/4), 32.60 (s, C15/16/19/20), 32.58 (s, C15/16/19/20), 28.3 (s, C15/16/19/20), 25.0 (s, C15/16/19/20); ^31^P{^1^H} NMR (THF-d_8_): *δ* [ppm] = 22.9 (d, ^1^*J*_P,Rh_ = 152.4 Hz); IR (KBr): *ν̃* [cm^−1^] = 3105 (w), 3092 (w), 3074 (w), 3054 (w), 2968 (m), 2934 (m), 2915 (m), 2831 (m, all *ν*(C–H)), 1970 (w), 1899 (w), 1769 (w, all aromatic overtones), 1597 (m), 1479 (m), 1434 (s, *ν*(C–P)), 1384 (m), 1333 (m), 1304 (m), 1261 (w), 1164 (s), 1111 (s), 1095 (s), 1061 (s), 1035 (s), 1029 (s), 997 (m), 961 (w), 921 (w), 899 (w), 871 (w), 832 (m), 813 (m), 746 (m), 702 (s), 694 (s), 626 (m), 541 (m), 522 (m), 498 (s), 470 (m), 445 (w), 430 (m); HRMS (ESI): *m*/*z* calcd for C_96_H_93_Cl_3_Fe_3_P_3_Rh_3_ [M]^+^ 1922.0776; found 1922.0763; elemental analysis calcd [%] for C_96_H_93_Cl_3_Fe_3_P_3_Rh_3_: C 59.98, H 4.83, found: C 60.16, H 4.97.

### Synthesis of μ_3_-[2,4,6-tris(1-diphenylphosphanyl-1′-ferrocenylene)-1,3,5-trifluorobenzene-1κ^1^*P*,2κ^1^*P*,3κ^1^*P*]-tris[chlorido-1,2,5,6-η^4^-(cycloocta-1,5-diene)rhodium(i)] ([1b(Rh)_3_])

[1b(Rh)_3_] was prepared analogously to [1a(Rh)_3_] using 1b (50.0 mg, 40.4 μmol, 1.00 eq.) and obtained as a fine orange powder in quantitative yield (80.0 mg) after precipitation by adding diethyl ether (THF : Et_2_O = 1 : 8 v/v).

M.p.: 188–191 °C (from THF/diethyl ether); ^1^H NMR (CD_2_Cl_2_): *δ* [ppm] = 7.77–7.49 (m, 12H, H11), 7.40–7.34 (m, 6H, H12), 7.33–7.26 (m, 12H, H10), 5.50 (s (br), *ω*_1/2_ = 11.3 Hz, 6H, H13/14/17/18), 5.01 (pt, *J*_H,H_ = 1.9 Hz, 6H, H4), 4.75 (pt, *J*_H,H_ = 1.9 Hz, 6H, H5), 4.70 (pq, *J* = 2.0 Hz, 6H, H8), 4.40 (pt, *J* = 2.0 Hz, 6H, H7), 3.13 (s (br), *ω*_1/2_ = 10.5 Hz, 6H, H14/13/18/17), 2.53–2.36 (m, 12H, H15/16/19/20), 2.15–2.03 (m, 6H, H15/16/19/20), 2.00–1.87 (m, 6H, H15/16/19/20); ^13^C{^1^H} NMR (CD_2_Cl_2_):^44^*δ* [ppm] = 155.7 (s, C1), 133.8 (d, ^3^*J*_C,P_ = 11.2 Hz, C11), 133.3 (d, ^1^*J*_C,P_ = 42.6 Hz, C9), 130.0 (d, ^4^*J*_C,P_ = 2.2 Hz, C12), 127.7 (d, ^2^*J*_C,P_ = 9.6 Hz, C10), 112.3 (s, C2), 104.9 (dd, ^2^*J*_C,P_ = 12.9 Hz, ^1^*J*_C,Rh_ = 6.9 Hz, C13/14/17/18), 75.9 (d, ^3^*J*_C,P_ = 9.9 Hz, C8), 74.7 (s, C3) 73.7 (d, ^2^*J*_C,P_ = 6.7 Hz, C7), 73.3 (d, ^1^*J*_C,P_ = 47.1 Hz, C6), 71.9 (s, C5), 71.7 (m, C4), 70.3 (d, ^1^*J*_C,Rh_ = 13.7 Hz, C14/13/18/17), 33.04 (s, C15/16/19/20), 33.01 (s, C15/16/19/20), 28.8 (s, C15/16/19/20); ^19^F{^1^H} NMR (CD_2_Cl_2_): *δ* [ppm] = −107.4 (s); ^31^P{^1^H} NMR (CD_2_Cl_2_): *δ* [ppm] = 22.7 (d, ^1^*J*_P,Rh_ = 151.8 Hz); IR (neat, ATR): *ν̃* [cm^−1^] = 3098 (w), 3068 (w), 3049 (w), 2959 (w), 2935 (m), 2914 (m), 2874 (m), 2828 (m, all *ν*(C–H)), 1479 (m), 1433 (s, *ν*(C–P)), 1422 (m, *ν*(C–F)), 1388 (m), 1332 (m), 1304 (m), 1273 (w), 1230 (w), 1218 (w), 1204 (w), 1177 (m, sh), 1163 (m), 1096 (m), 1066 (m), 1024 (s), 997 (m), 960 (w), 912 (w), 888 (w), 859 (w), 832 (m), 815 (m), 763 (m, sh), 745 (s), 692 (*vs.*), 627 (m), 540 (m), 521 (s), 497 (*vs.*), 488 (*vs.*), 468 (*vs.*), 428 (s); HRMS (ESI): *m*/*z* calcd for C_96_H_90_Cl_3_F_3_Fe_3_P_3_Rh_3_ [M − Cl]^+^ 1941.0813; found 1941.0819; elemental analysis calcd [%] for C_96_H_90_Cl_3_F_3_Fe_3_P_3_Rh_3_: C 58.34, H 4.59, found: C 58.02, H 4.69.

### Synthesis of μ_3_-[2,4,6-tris(1-diphenylphosphanyl-1′-ferrocenylene)-1,3,5-triazine-1κ^1^*P*,2κ^1^*P*,3κ^1^*P*]-tris[chlorido-1,2,5,6-η^4^-(cycloocta-1,5-diene)rhodium(i)] ([1c(Rh)_3_])

[1c(Rh)_3_] was prepared analogously to [1a(Rh)_3_] using 1c (50.0 mg, 42.2 μmol, 1.00 eq.) and obtained as a red powder (50.0 mg, 62%) which already partly precipitated from the reaction mixture.

M.p.: 246–248 °C (from THF/diethyl ether); ^1^H NMR (CD_2_Cl_2_): *δ* [ppm] = 7.63–7.50 (m, 12H, H10), 7.42–7.26 (m, 18H, H9 + H11), 5.55 (s (br), *ω*_1/2_ = 11.0 Hz, 6H, H12/13/16/17), 5.53 (pt, *J*_H,H_ = 2.0 Hz, 6H, H3), 4.93 (pt, *J*_H,H_ = 2.0 Hz, 6H, H4), 4.71 (pq, *J* = 2.0 Hz, 6H, H7), 4.26 (pt, *J* = 2.0 Hz, 6H, H6), 3.19 (s (br), *ω*_1/2_ = 10.6 Hz, 6H, H13/12/17/16), 2.56–2.42 (m, 12H, H14/15/18/19), 2.20–2.06 (m, 6H, H14/15/18/19), 2.05–1.94 (m, 6H, H14/15/18/19); ^13^C{^1^H} NMR (CD_2_Cl_2_): *δ* [ppm] = 175.5 (s, C1), 133.9 (d, ^3^*J*_C,P_ = 11.1 Hz, C10), 133.1 (d, ^1^*J*_C,P_ = 42.4 Hz, C8), 130.0 (d, ^4^*J*_C,P_ = 2.2 Hz, C11), 127.8 (d, ^2^*J*_C,P_ = 9.7 Hz, C9), 105.0 (dd, ^2^*J*_C,P_ = 12.7 Hz, ^1^*J*_C,Rh_ = 6.9 Hz, C12/13/16/17), 81.2 (s, C2), 76.0 (d, ^3^*J*_C,P_ = 9.7 Hz, C7), 74.9 (s, C4), 74.2 (d, ^1^*J*_C,P_ = 46.4 Hz, C6), 73.9 (d, ^2^*J*_C,P_ = 6.6 Hz, C8), 71.4 (s, C3), 70.3 (d, ^1^*J*_C,Rh_ = 13.7 Hz, C13/12/17/16), 33.12 (s, C14/15/18/19), 33.09 (s, C14/15/18/19), 28.8 (s, C14/15/18/19); ^31^P{^1^H} NMR (CD_2_Cl_2_): *δ* [ppm] = 22.4 (d, ^1^*J*_P,Rh_ = 152.0 Hz); IR (neat, ATR): *ν̃* [cm^−1^] = 3092 (w), 3071 (w), 3050 (w), 2992 (w), 2936 (w), 2914 (w), 2875 (m), 2828 (m, all *ν*(C–H)), 1506 (*vs.*), 1481 (s), 1434 (m, *ν*(C–P)), 1394 (w), 1379 (m), 1355 (m), 1332 (w), 1319 (m), 1305 (m), 1218 (w), 1161 (m), 1096 (m), 1072 (w), 1054 (w), 1027 (m), 997 (m), 960 (w), 926 (w), 889 (w), 859 (w), 832 (m), 760 (m), 743 (s), 691 (s), 626 (m), 502 (*vs.*), 471 (s), 425 (s); HRMS (ESI): *m*/*z* calcd for C_93_H_90_Cl_2_Fe_3_N_3_P_3_Rh_3_ [M − Cl]^+^ 1890.0952; found 1890.0957; elemental analysis calcd [%] for C_93_H_90_Cl_3_Fe_3_N_3_P_3_Rh_3_: C 58.02, H 4.71, N 2.18, found: C 57.25, H 4.66, N 2.16.

### Synthesis of μ_3_-[1,3,5-tris{(1-diphenylphosphanyl-1′-ferrocenylene)methyl}benzene-1κ^1^*P*,2κ^1^*P*,3κ^1^*P*]-tris[chlorido-1,2,5,6-η^4^-(cycloocta-1,5-diene)rhodium(i)] ([1d(Rh)_3_])

[1d(Rh)_3_] was prepared analogously to [1a(Rh)_3_] using 1d (50.0 mg, 40.8 μmol, 1.00 eq.). For precipitation, significantly larger amounts of diethyl ether, hexanes (THF : Et_2_O : hexanes = 1 : 10 : 4 v/v/v) and low temperatures (−20 °C) had to be used. The very fine, light yellow precipitate was filtered over degassed Celite and recovered by dissolution in THF. Removal of the volatiles gave [1d(Rh)_3_] in 20% yield (16 mg).

M.p.: >160 °C decomp. (from THF); ^1^H NMR (THF-d_8_): *δ* [ppm] = 7.65–7.55 (m, 12H, H12), 7.40–7.27 (m, 18H, H11 + H13), 6.86 (s, 3H, H1), 5.49 (s (br), *ω*_1/2_ = 11.2 Hz, 6H, H14/15/18/19), 4.71 (pq, *J* = 2.0 Hz, 6H, H9), 4.36–4.29 (m, 6H, H8), 4.28 (s, 12H, H5 + H6), 3.72 (s, 6H, H3), 3.06 (s, 6H, H14/15/18/19), 2.43–2.27 (m, 12H, H16/17/20/21), 2.07–1.97 (m, 6H, H16/17/20/21), 1.92–1.83 (m, 6H, H16/17/20/21); ^13^C{^1^H} NMR (THF-d_8_): *δ* [ppm] = 142.4 (s, C2), 135.1 (d, ^1^*J*_C,P_ = 42.5 Hz, C10), 135.0 (d, ^3^*J*_C,P_ = 10.9 Hz, C12), 130.5 (d, ^4^*J*_C,P_ = 2.1 Hz, C13), 128.4 (d, ^2^*J*_C,P_ = 9.5 Hz, C11), 126.9 (s, C1), 105.0 (dd, ^2^*J*_C,P_ = 13.2 Hz, ^1^*J*_C,Rh_ = 7.1 Hz, C14/15/18/19), 90.8 (s, C4), 76.4 (d, ^3^*J*_C,P_ = 10.5 Hz, C9), 74.1 (d, ^1^*J*_C,P_ = 47.9 Hz, C7), 72.6 (d, ^2^*J*_C,P_ = 6.9 Hz, C8), 71.5 (s, C5/C6), 71.2 (s, C6/C5), 70.7 (d, ^1^*J*_C,Rh_ = 13.6 Hz, C15/14/19/18), 36.4 (s, C3), 33.88 (s, C16/17/20/21), 33.86 (s, C16/17/20/21), 29.6 (s, C16/17/20/21); ^31^P{^1^H} NMR (THF-d_8_): *δ* [ppm] = 22.9 (d, ^1^*J*_P,Rh_ = 152.2 Hz); IR (neat, ATR): *ν̃* [cm^−1^] = 3073 (m), 3051 (m), 2996 (m), 2934 (m), 2912 (s), 2876 (s), 2828 (s, all *ν*(C–H)), 1979 (w), 1600 (m), 1572 (w), 1526 (w), 1480 (m), 1433 (*vs.*, *ν*(C–P)), 1387 (w), 1333 (m), 1305 (m), 1229 (m), 1160 (s), 1096 (s), 1059 (m), 1027 (s), 997 (m), 961 (m), 925 (w), 899 (w), 857 (m), 829 (m), 815 (m), 744 (s), 702 (s), 693 (s), 625 (m), 540 (m), 522 (m), 496 (s), 467 (s), 445 (m), 426 (m), 406 (m); HRMS (ESI): *m*/*z* calcd for C_96_H_99_Cl_2_Fe_3_P_3_Rh_3_ [M − Cl]^+^ 1929.1567; found 1929.1592; elemental analysis calcd [%] for C_99_H_99_Cl_3_Fe_3_P_3_Rh_3_: C 60.53, H 5.08, found: C 59.16, H 5.16.^45^

## Results and discussion

### Synthesis and characterisation

Reacting the tris-phosphanes 1a–d, prepared according to the published procedure,^30,31^ with the suitable precursor compounds [{RuCl_2_(*p*-cym)}_2_] (*p*-cym = η^6^-*p*-cymene) or [{RhCl(1,5-cod)}_2_] (1,5-cod = η^4^-cycloocta-1,5-diene) in CH_2_Cl_2_ at r.t. in slight stoichiometric excess (1 : 3) afforded, after simple work-up procedures, the homotrinuclear metal complexes [1a–d(Ru)_3_] and [1a–d(Rh)_3_] in good to excellent yields (Scheme 1).

NMR spectroscopy (^31^P{^1^H} NMR chemical shifts for all complexes are presented in Table 1) does not suggest hindered rotation about the arene–ferrocenylene bonds, and all complexes remain homotrinuclear in the gas phase as assessed by high-resolution electrospray-ionisation mass spectrometry (HR-ESI MS, see ESI†).

**Table 1:** ^31^P{^1^H} NMR chemical shifts (in ppm) of ligands 1a–d^30,31^ and their corresponding homotrinuclear complexes with RuCl_2_(*p*-cym) or RhCl(1,5-cod), determined in CD_2_Cl_2_

	a	b	c	d
1	−17.5 (ref. 30)	−17.6 (ref. 30)	−18.4 (ref. 31)	−17.1 (ref. 30)
[1(Ru)_3_]	18.9	19.4	18.8	18.8
[1(Rh)_3_]	22.9^a^	22.7	22.4	22.9^a^

a In THF-d_8_.

**Fig. 1:**
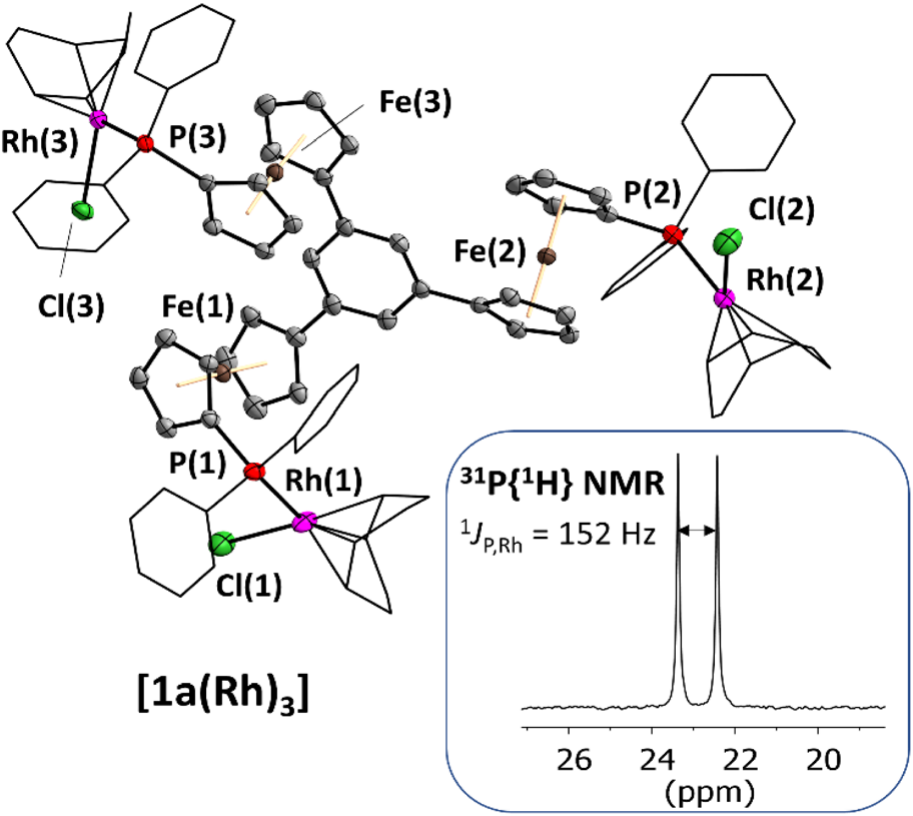
Molecular structure of the homotrinuclear rhodium(i) complex [1a(Rh)_3_] with part of the atom-numbering scheme and the ^31^P{^1^H} NMR signal in THF-d_8_ solution, featuring the characteristic ^1^*J* coupling between ^31^P and ^103^Rh. Thermal ellipsoids are set at the 50% probability level. For clarity, the phenyl rings and 1,5-cyclooctadiene ligands are depicted in wireframe style, and co-crystallised solvent and hydrogen atoms have been omitted.

Single crystals of [1a(Rh)_3_] suitable for XRD were obtained by slow diffusion of diethyl ether into a THF solution, confirming the trinuclear nature of the complex in the solid state (Fig. 1). As most of the few reported crystal structures of Rh_3_P_3_ complexes contain triangular or linear Rh_3_-derived cores,^46–48^[1a(Rh)_3_] is, to the best of our knowledge, only the second tris-phosphane-based example. The other entry features rhodium(iii) atoms coordinated by tris(2-diphenylphosphanylethyl)amine and includes one Rh–N bond.^49^ Balakrishna and co-workers have prepared *C*_3_-symmetric rhodium(i) tris–phosphane complexes, yet have not been able to determine their solid-state molecular structures.^50^ Tris(*N*-heterocyclic carbene)^51^ and tris(pyridyl) ligands^52,53^ have also been used for the preparation of *C*_3_-symmetric trinuclear rhodium complexes. Surprisingly, even though many ferrocenylphosphane rhodium complexes are listed in the CSD, the simple diphenylferrocenylphosphane cyclooctadiene (cod) rhodium(i) chloride moiety has not yet been crystallographically described; cationic complexes like the planar chiral diphenylphosphinoferrocenylthioether-derived rhodium(cod) complexes by Manoury and co-workers are not well comparable to [1a(Rh)_3_].^54^ The closest analogue, a [1]phosphaferrocenophane-derived complex by Breher and co-workers,^55^ compares favourably with [1a(Rh)_3_] (Table 2; more information in the ESI†) regarding the Rh–P bond lengths of 2.304–2.314 Å (their work: 2.296 Å).

**Table 2:** Selected bond lengths and metal–metal distances [Å] and angles [°] of complex [1a(Rh)_3_], numbering scheme according to Fig. 1. A more detailed overview can be found in the ESI

	[1a(Rh)_3_]
Rh(1,2,3)–P(1,2,3)	2.314(1)/2.382(1)/2.307(1)
Rh(1,2,3)–Cl(1,2,3)	2.355(1)/2.304(1)/2.370(1)
P(1,2,3)–Rh(1,2,3)–Cl(1,2,3)	91.28(4)/88.98(4)/91.38(4)
Rh(1,2,3)⋯Rh(2,3,1)^a^	11.5266(7)/15.9121(7)/11.5422(5)/(*6.1288*(*6)*)
Rh(1,2,3)⋯Fe(1,2,3)	4.289(1)/4.428(1)/4.460(1)

a Intramolecular distances; the shortest intermolecular distance is given italicised and in brackets.

No single crystals suitable for XRD analysis could be obtained for the ruthenium(ii) complexes [1a–d(Ru)_3_] which were, however, fully characterised spectroscopically and by HR-ESI MS. In CD_2_Cl_2_ or THF-d_8_ they undergo a slow chemical transformation, liberating *p*-cymene, exemplarily shown by ^1^H and ^31^P{^1^H} NMR spectroscopy of [1a(Ru)_3_] (*cf.* ESI). This process is also solvent-dependent. We speculate that the loss of *p*-cymene is induced by either intra- or intermolecular η^6^-coordination of one of the phenyl rings in the PPh_2_ moiety. The addition of three equivalents of *p*-cymene slowed down this degradation, which is thus most likely connected to the intra- or intermolecular substitution by a P-bound phenyl ring,^56,57^ a process we have recently employed to prepare tethered *P*-chiral ruthenium(ii) complexes.^23^ In the present case, the resulting products are hardly soluble and likely oligomeric or polymeric. These findings notwithstanding, compounds [1a–d(Ru)_3_] expand the scope of trinuclear ruthenium complexes and are the first non-cluster examples to incorporate more than one ferrocenyl moiety in the complex.^58^

### Cyclic voltammetry

**Fig. 2:**
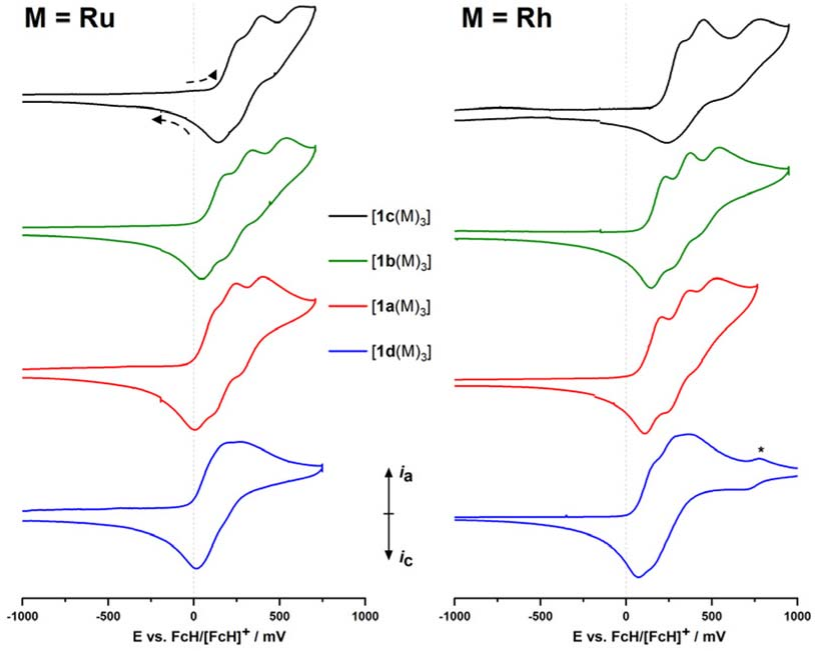
Partial cyclic voltammograms (iron-centred oxidations only) of ruthenium (left) and rhodium (right) complexes [1a–d(M)_3_] at *ca.* 1 mmol L^−1^ in 0.1 mol per L (^*n*^Bu_4_N)BAr^F^_4_/CH_2_Cl_2_ (scan rate: 100 mV s^−1^, working electrode: glassy carbon, counter electrode: platinum wire). The 2^nd^ of three cycles is shown for all compounds, recorded currents are shown normalised for easier comparison. Scanning direction as indicated. The rhodium-centred oxidation event for [1d(Rh_3_)] is marked with an asterisk (*). For full voltammograms, see ESI (Section 5).†

Complexes [1a–d(Ru)_3_] and [1a–d(Rh)_3_] are electrochemically active (Fig. 2). As a general observation, the electrochemistry in a BF_4_^−^-based supporting electrolyte (SE) system is more complicated, less reversible, and hence more difficult to interpret. Accordingly, the BAr^F^_4_^−^-based SE was used which enables a distinct separation of the three iron-centred oxidations of the ligand backbone. Under these conditions, these oxidations are mostly reversible but, especially for the third oxidation, already show signs of irreversibility. The system with no deviations from (quasi-)reversibility, in all cases, is the C_6_H_3_-based ligand (1a); all other systems show reduced reversibility, most likely arising from follow–up reactions (for a detailed discussion of the complexes' electrochemistry as probed by cyclic voltammetry and, in selected cases, differential pulse voltammetry, see the ESI, Section 5†), which makes them less well-suited for potential applications in redox-switchable catalysis which relies on fully reversible redox events of the catalyst to ensure full control.

In general, while the coordinated metal complex fragment does not strongly alter the electrochemical response of the arene-trisferrocenyl core, there are observable differences. Rhodium(i) complexes are oxidised at slightly higher potentials than ruthenium(ii) complexes (Table 3). For both metal fragments, individual oxidation states for the C_6_H_3_(CH_2_)_3_-based complexes are difficult to address and the C_3_N_3_-based complexes show severely broadened third, Fe-centred oxidations and reduced reversibility, meaning that the initial system cannot be fully restored under these conditions.

**Table 3:** Redox potentials for the first iron-centred oxidation *E*^0^_1_ (*vs.* FcH/[FcH]^+^) of 1a–d,^30,31^[1a–d(Rh)_3_] and [1a–d(Ru)_3_], in 0.1 mol per L (^*n*^Bu_4_N)BAr^F^_4_/CH_2_Cl_2_ determined by cyclic voltammetry (BAr^F^_4_ = [B{3,5-(CF_3_)_2_C_6_H_3_}_4_]).^a^

*E* ^0^_1_ (Δ*E*_p_)^a^ [mV]					
1a^30^	138 (98)	[1a(Ru)_3_]	64 (118)	[1a(Rh)_3_]	158 (99)
1b^30^	206 (116)	[1b(Ru)_3_]	116 (140)	[1b(Rh)_3_]	190 (86)
1c^31^	275 (160)	[1c(Ru)_3_]	204 (122)	[1c(Rh)_3_]	283 (86)
1d^30^	113^b^	[1d(Ru)_3_]	91 (148)	[1d(Rh)_3_]	116 (93)

a Potentials *vs.* the FcH/[FcH]^+^ couple at a glassy carbon working electrode (scan rate 100 mV s^−1^). Determined on 1 mmol per L samples in anhydrous 0.1 mol per L (*n*Bu_4_N)BAr^F^_4_/CH_2_Cl_2_ as SE (working electrode: glassy carbon). The difference between oxidation and reduction potential, Δ*E*_p_, is given in brackets.

b Determined from square-wave voltammetry due to close peak-to-peak separation, leaving Δ*E*_p_ inaccessible.

Oxidation events that are likely associated with the coordinated metal complex fragments can be well separated from the ligand-centred oxidations. Thus, a fourth, likely Rh-centred oxidation can be observed for the less electron-withdrawing ligands, but for ligands with a C_6_F_3_ or C_3_N_3_ core, the fourth oxidation event is outside the window of the electrochemical stability of the supporting electrolyte (see ESI,† Section 5). [1a–d(Rh)_3_] show metal-centred oxidations owing to the redox-active rhodium(i) centres. The peak potentials for the Rh^I^/Rh^II^ couple in [1a(Rh)_3_] (751 mV with BF_4_^−^, 854 mV with BAr^F^_4_^−^) are significantly higher and the oxidations less reversible than for a related P-ferrocenophane-derived chlorido(cyclooctadiene)rhodium(i) complex (*E*^0^ = 390 mV *vs.* FcH/[FcH]^+^) reported by Breher and co-workers.^55^ This reduced reversibility might be tied to the fact that the oxidation of the rhodium centre will most likely generate a quadruply charged cation.

In general, all ruthenium(ii) complexes show a very similar electrochemical fingerprint under the given conditions, but more oxidation events associated with the coordinated metal are observed within the available electrochemical window. When measured in the BAr^F^_4_^−^-based SE, the ruthenium-centred oxidations split into two distinct yet irreversible oxidation events, apparently consisting of one 1e^−^- and one 2e^−^-transfer steps. As an electrochemical comparison for [1a(Ru)_3_], a tethered (1′-methoxy-1-ferrocenylene)-based diarylphosphane ruthenium(ii) complex, reported previously by us, is well suited and shows similar redox properties with *E*^0^(Fe^II^/Fe^III^) = 110 mV and *E*^ox^(Ru^II^/Ru^III^) = 700 mV *vs.* FcH/[FcH]^+^ in (^*n*^Bu_4_N)PF_6_/CH_2_Cl_2_.^59^

For both metal complex fragments, the onset of ligand oxidation is determined by the arene substitution pattern and follows the expected order with 1d being the easiest, 1c the hardest to oxidise. None of the Ru/Rh-centred oxidations appear to be electrochemically reversible (see ESI,† Section 5). Consequently, redox state control of the metal centre, another variation of redox-switchable catalysis, is not possible with these complexes. In conclusion, this means that, among the systems investigated in this study, fully reversible ligand-centred redox control is most promising for [1a(M)_3_] (M = Ru, Rh).

## Conclusions

We have demonstrated the synthesis of rhodium(i) and ruthenium(ii) complexes with the tris(ferrocenyl)arene-based tris-phosphanes 1a–d, which form well-defined, *C*_3_-symmetric homotrinuclear transition metal complexes with four accessible oxidation states relating to the tris(ferrocenyl)arene backbone. With a BAr^F^_4_^−^-based supporting electrolyte, a distinct separation of the three iron-centred oxidations of the ligand backbone was observed, making these complexes potentially suitable for redox-switchable catalysis or other applications in which control of the charge state of the system could be of interest, such as electrochromic films or materials with switchable surface characteristics.^59,60^

## Data availability

The data supporting this article have been included as part of the ESI.†

## Author contributions

A. S. has carried out the syntheses and characterisation of the compounds, including the electrochemical experiments. P. C. has acquired and solved the solid-state structure. E. H.-H. has supervised and administered the project, helped in acquiring funding for A. S. and P. C.; E. H.-H. and A. S. wrote the first draft and revised the manuscript according to the referee comments.

## Conflicts of interest

There are no conflicts to declare.

## Supplementary Material

RA-014-D4RA03822C-s001

RA-014-D4RA03822C-s002
