# Supplementary material for: Homotrinuclear ruthenium(ii) and rhodium(i) complexes of redox-active tris(ferrocenyl)arene-based tris-phosphanes
Source: RSC Adv. 2024 Aug 6;14(34):24652–60. doi: 10.1039/d4ra03822c (PMC11302318; doi:10.1039/d4ra03822c)
Supplement: RA-014-D4RA03822C-s001 [file RA-014-D4RA03822C-s001.pdf]

Supplementary Information (SI) for RSC Advances.
This journal is © The Royal Society of Chemistry 2024

SUPPORTING INFORMATION

Homotrinuclear ruthenium(II) and rhodium(I) complexes of redox-active tris(ferrocenyl)arene-based tris-phosphanes

Axel Straube,[a] Peter Coburger,[b] and Evamarie Hey-Hawkins*[a]

Table of contents

1. Atom numbering scheme for assignments in NMR spectra 1

2. NMR spectra of complexes **[1a-d(Ru)$_3$]** and **[1a-d(Rh)$_3$]** 2

3. Stability of **[1a(Ru)$_3$]** in CD_2Cl_2 and THF-d_8 18

4. Single crystal X-ray diffraction analyses 20

5. Electrochemistry of complexes **[1a-d(Ru)$_3$]** and **[1a-d(Rh)$_3$]** 22

[a] Dr. A. Straube, Dr. P. Coburger Prof. Dr. E. Hey-Hawkins, Institute of Inorganic Chemistry, Leipzig University, Johannisallee 29, D-04103 Leipzig (Germany). E-mail: hey@uni-leipzig.de
[a] New address: Dr. A. Straube, Wiley-VCH, Boschstr. 12, D-69469 Weinheim (Germany)
[b] New address: Dr. P. Coburger, Department of Inorganic Chemistry, TU München, Lichtenbergstraße 4, 85747 Garching (Germany)

1. Atom numbering scheme for assignments in NMR spectra

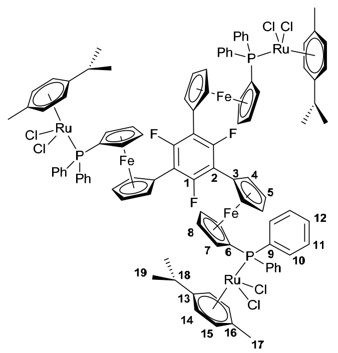

[1a(Ru)$_3$]

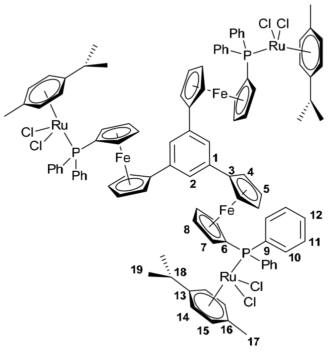

[1b(Ru)$_3$]

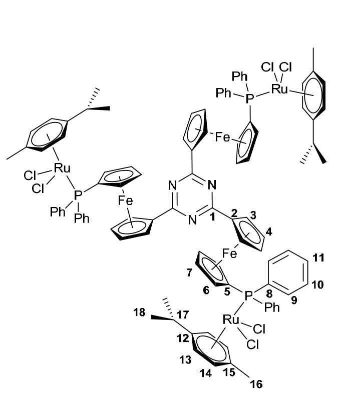

[1c(Ru)$_3$]

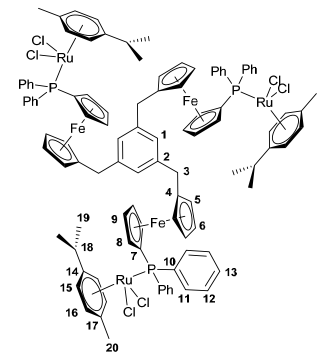

[1d(Ru)$_3$]

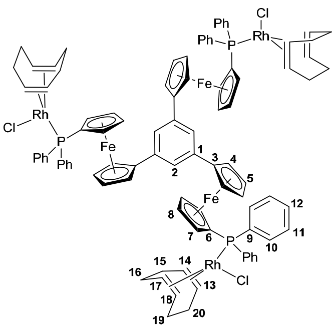

[1a(Rh)$_3$]

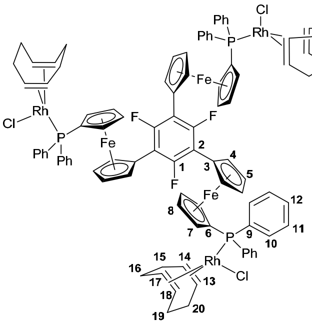

[1b(Rh)$_3$]

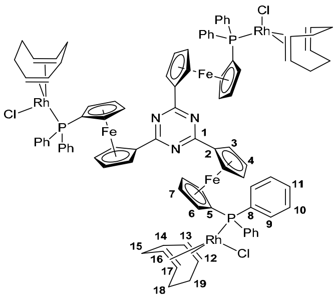

[1c(Rh)$_3$]

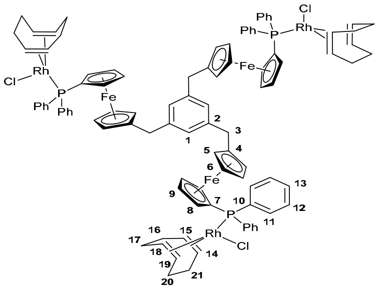

[1d(Rh)$_3$]

2. NMR spectra of complexes [1a-d(Ru)3] and [1a-d(Rh)3]

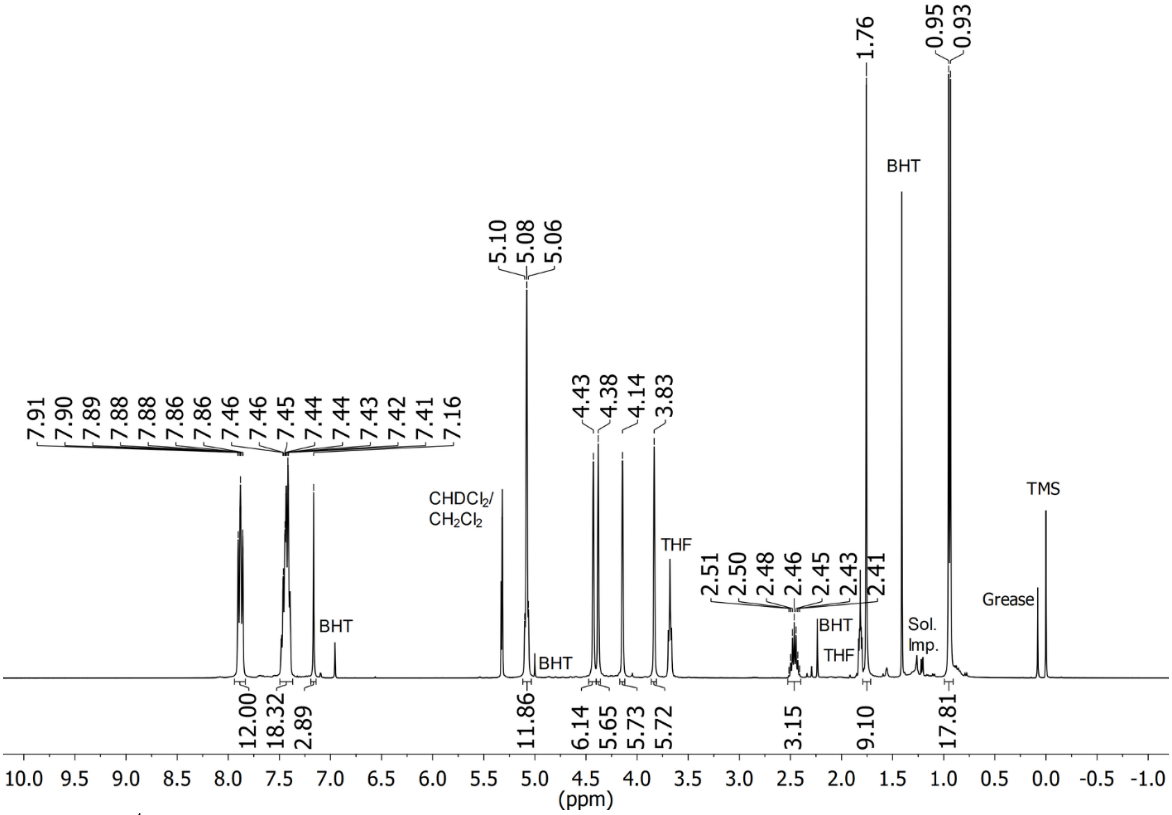

Figure S1. ^{1}H NMR spectrum of **[1a(Ru)3]** in CD_2Cl_2 [BHT: dibutylhydroxytoluene (stabiliser in THF)]

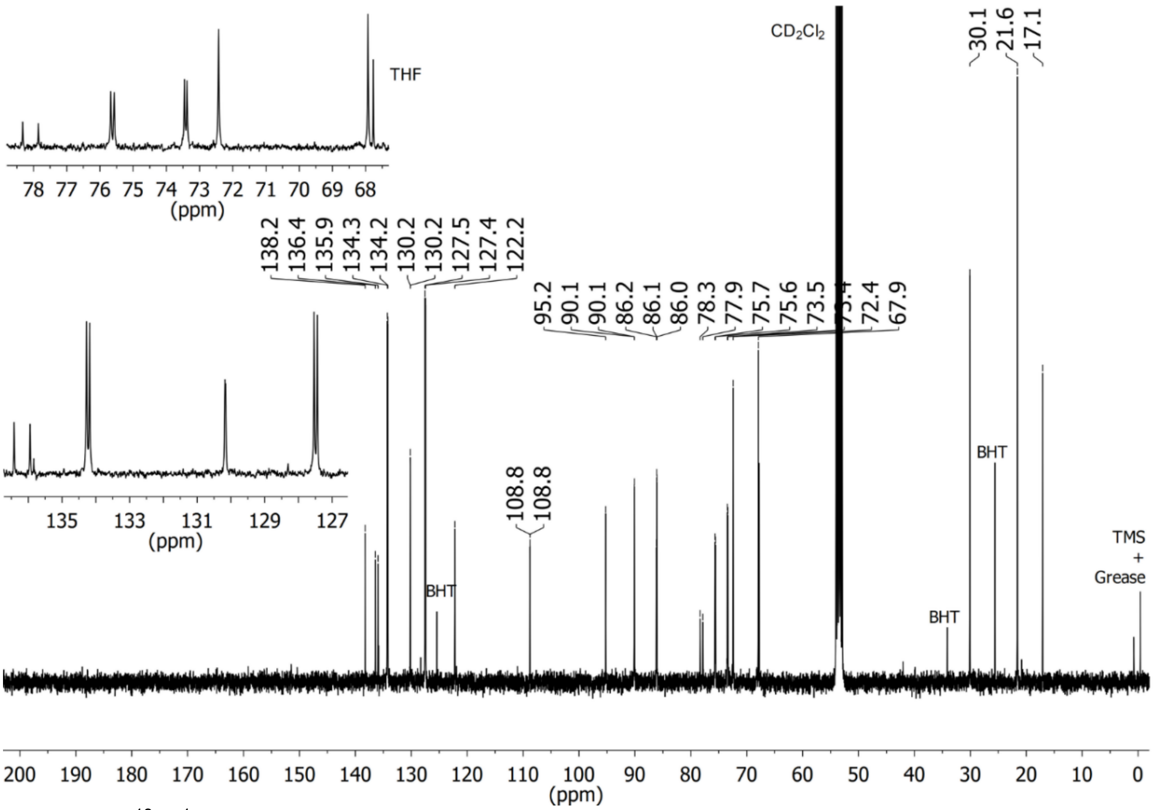

Figure S2. ^{13}C{^{1}H} NMR spectrum of **[1a(Ru)3]** in CD_2Cl_2 [BHT: dibutylhydroxytoluene (stabiliser in THF)].

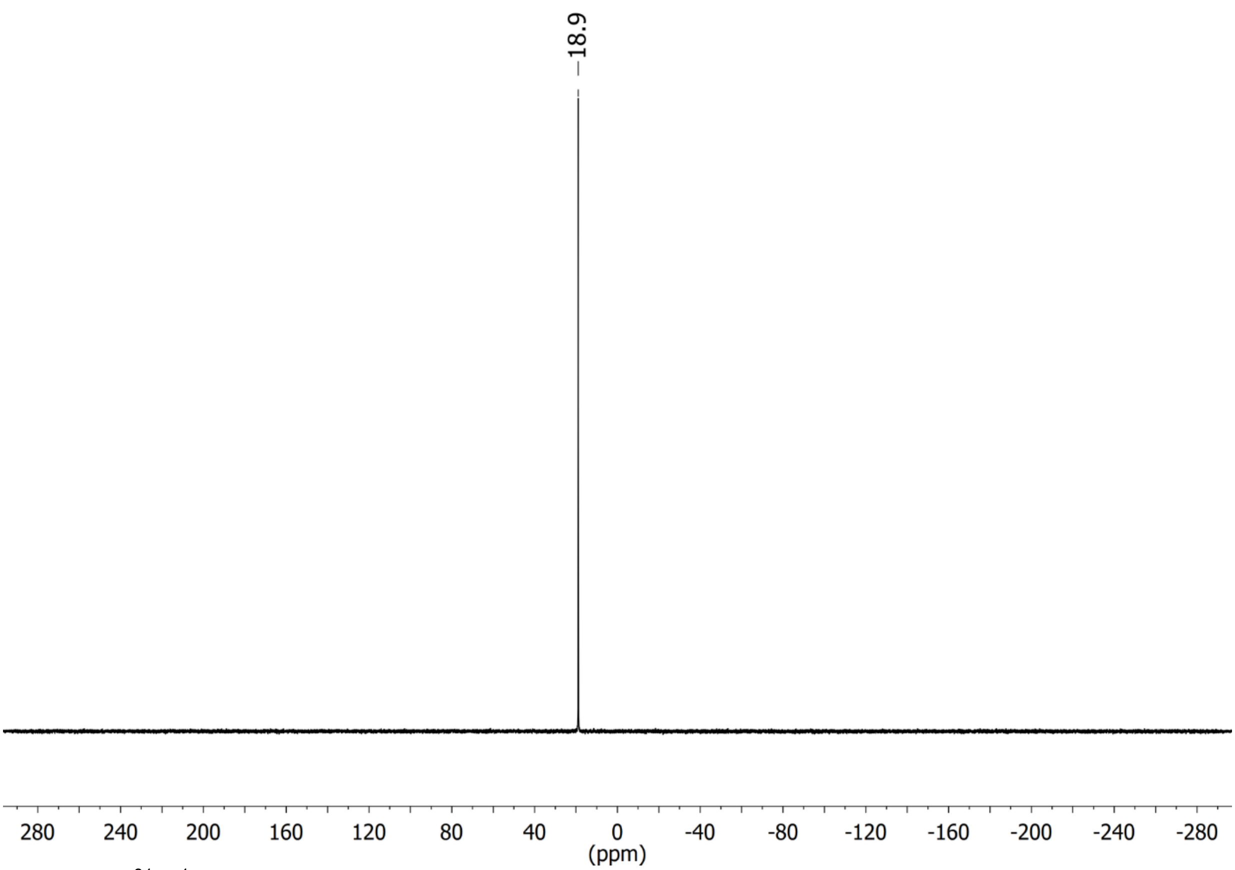

Figure S3. $^{31}P\{^{1}H\}$ NMR spectrum of **[1a(Ru)$_3$]** in CD_2Cl_2

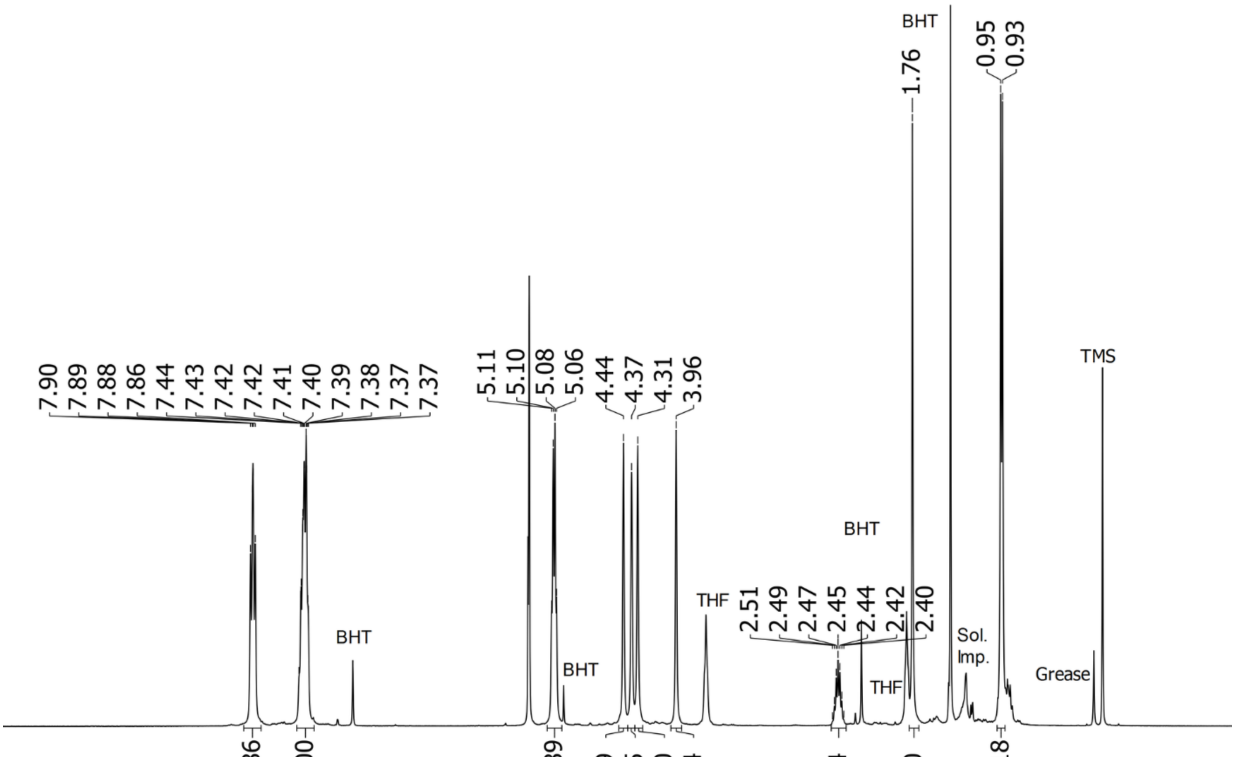

Figure S4. ^{1}H NMR spectrum of **[1b(Ru)$_3$]** in CD_2Cl_2 [BHT: dibutylhydroxytoluene (stabiliser in THF)].

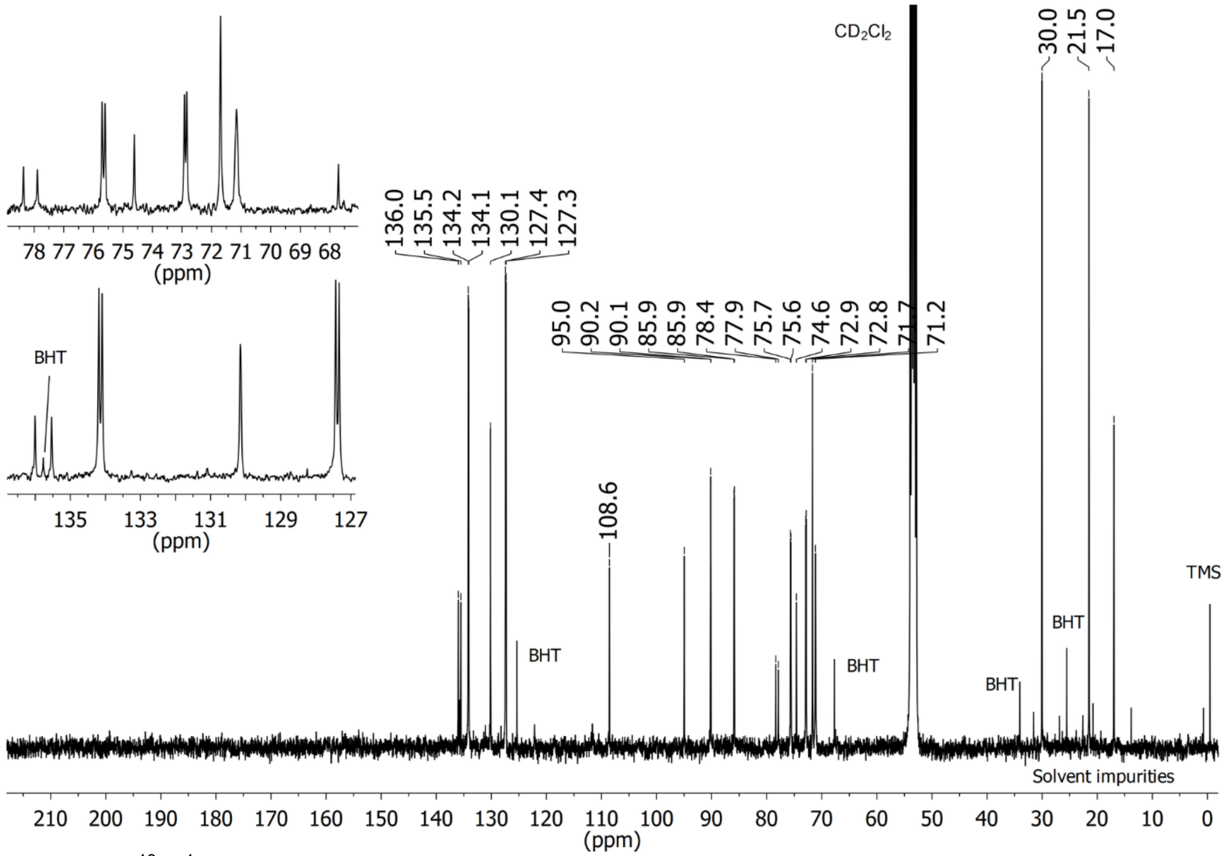

Figure S5. $^{13}C\{^1H\}$ NMR spectrum of **[1b(Ru)$_3$]** in CD_2Cl_2 [BHT: dibutylhydroxytoluene (stabiliser in THF)].

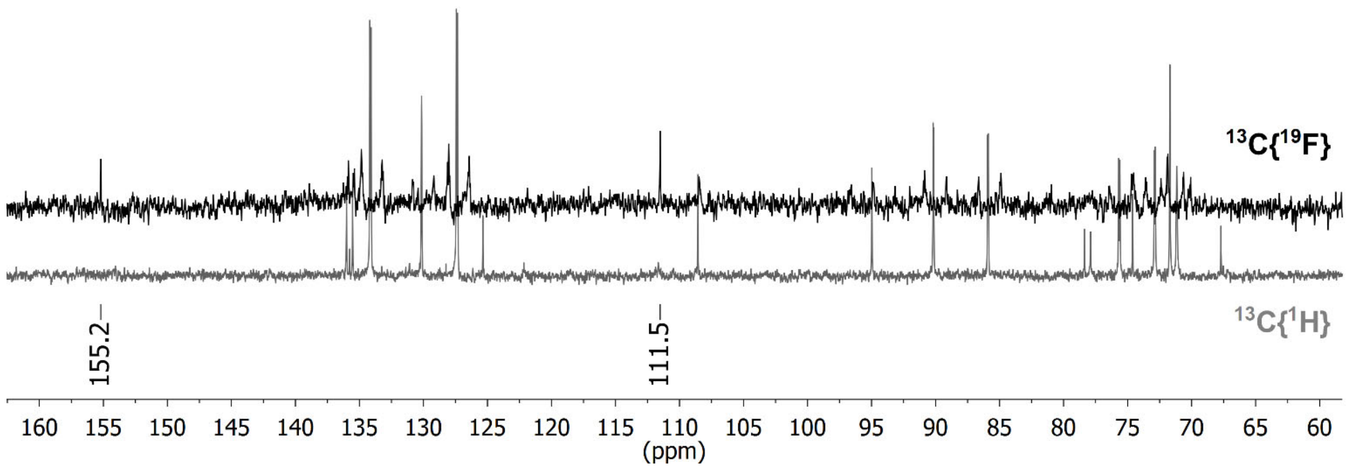

Figure S6. $^{13}C\{^{19}F\}$ NMR spectrum of **[1b(Ru)$_3$]** in CD_2Cl_2

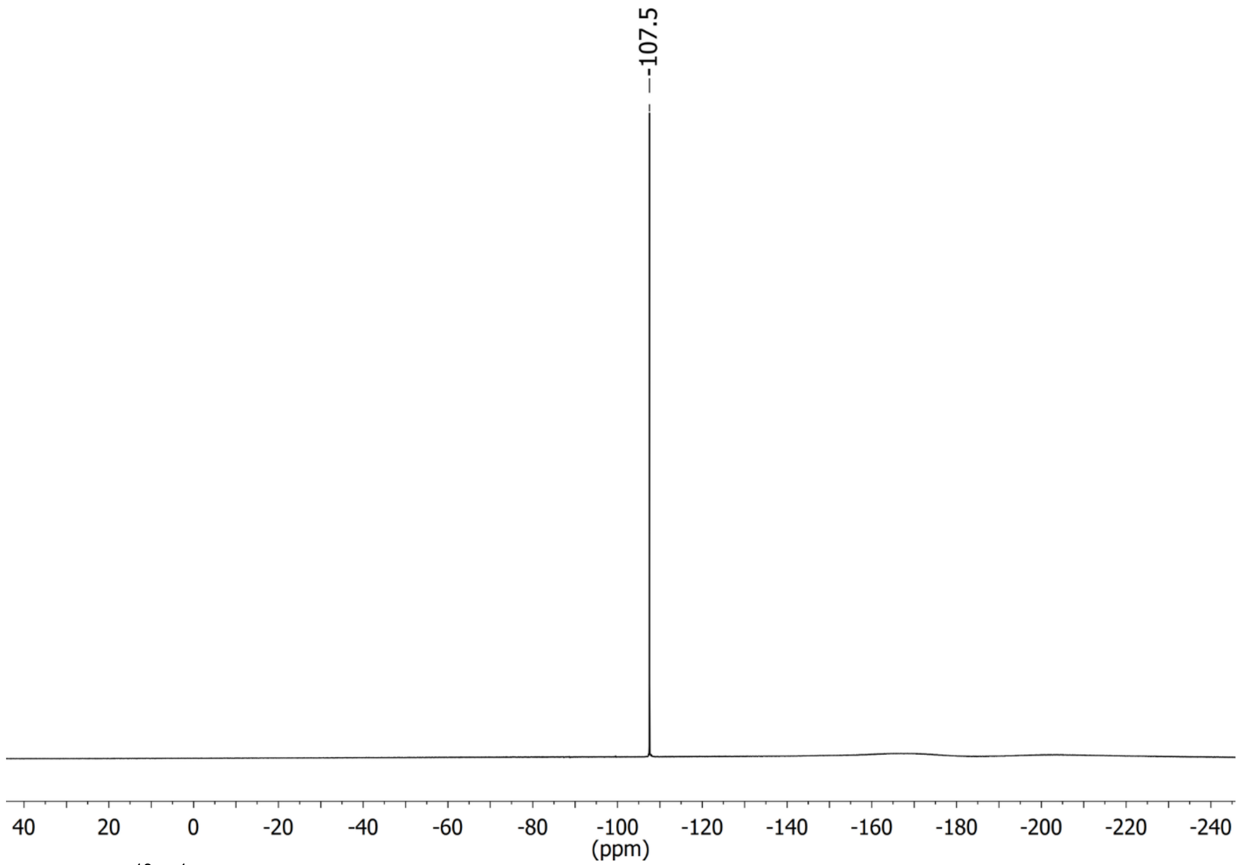

Figure S7. $^{19}F\{^{1}H\}$ NMR spectrum of **[1a(Ru)3]** in CD_2Cl_2

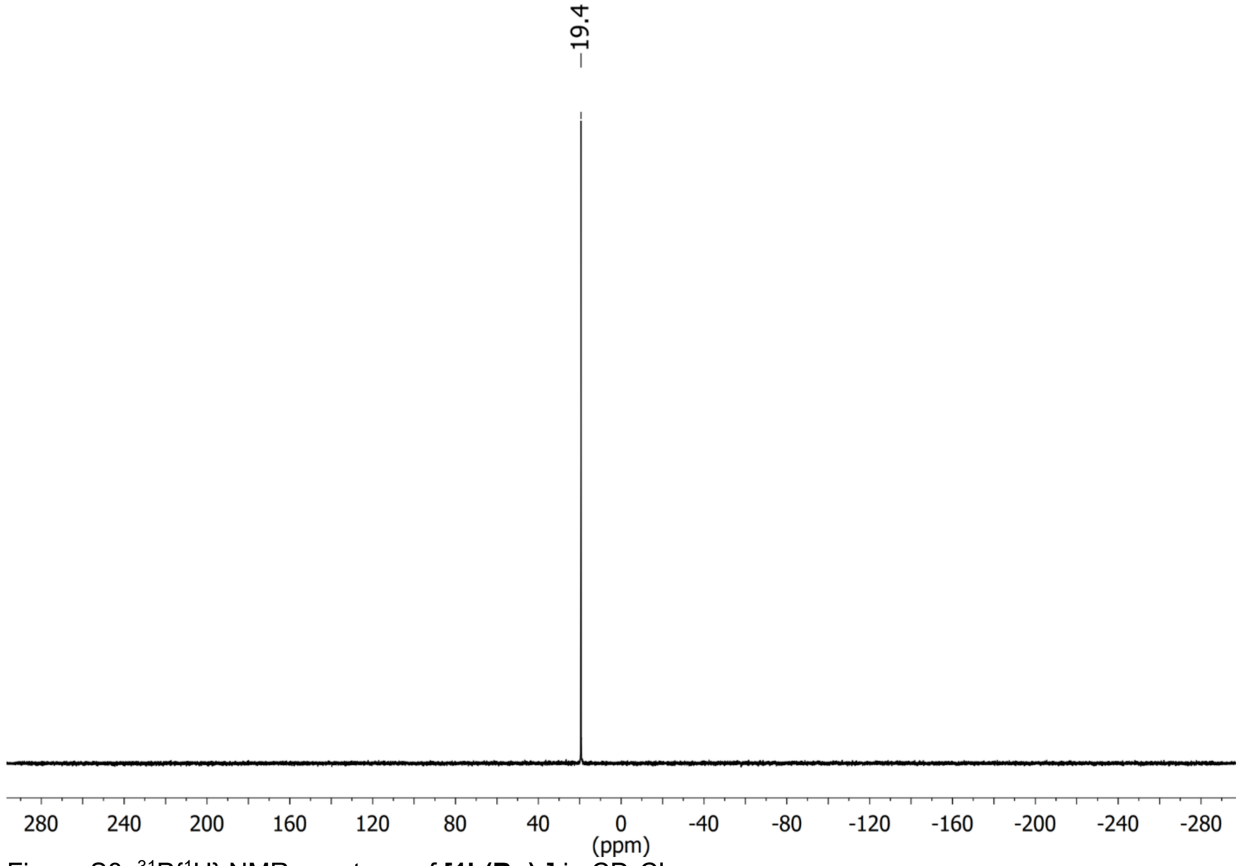

Figure S8. $^{31}P\{^{1}H\}$ NMR spectrum of **[1b(Ru)3]** in CD_2Cl_2

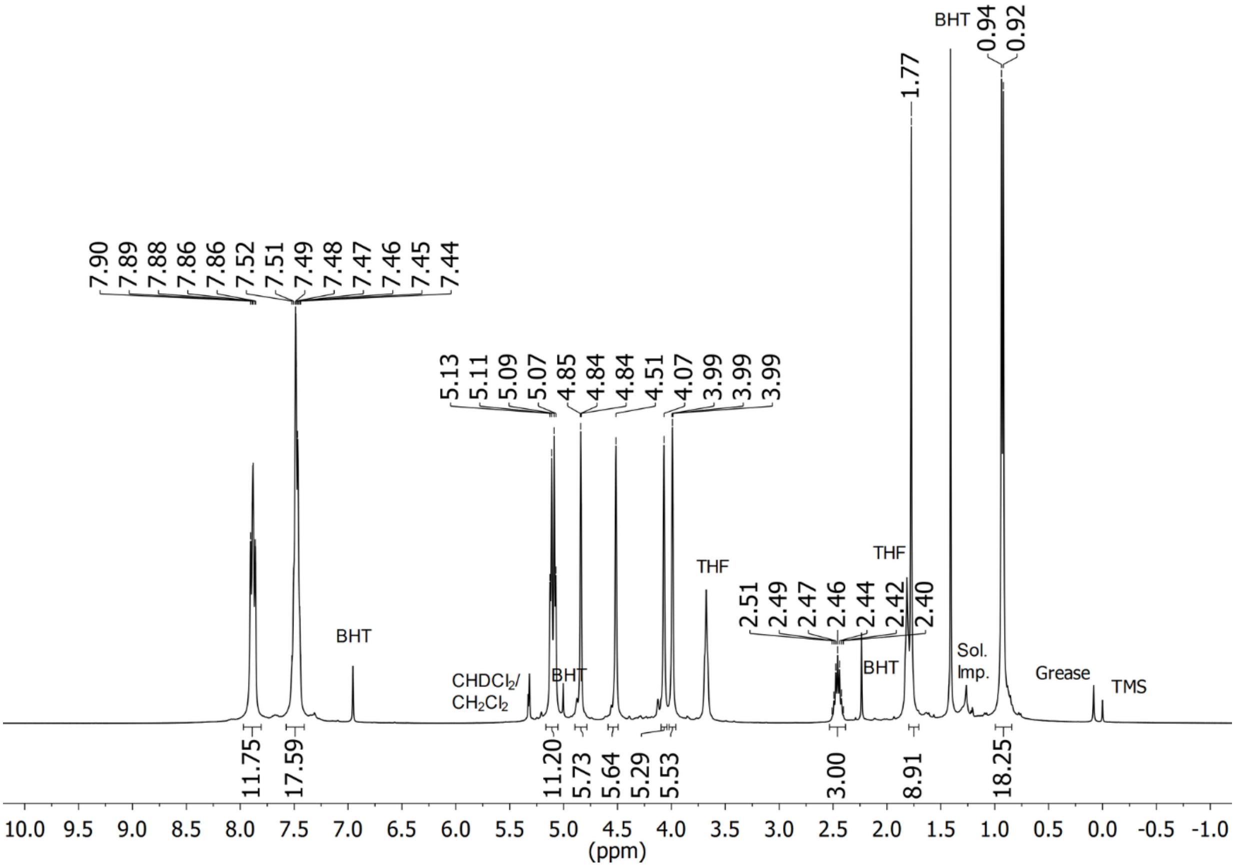

Figure S9. ^{1}H NMR spectrum of **[1c(Ru)$_3$]** in CD_2Cl_2 [BHT: dibutylhydroxytoluene (stabiliser in THF)].

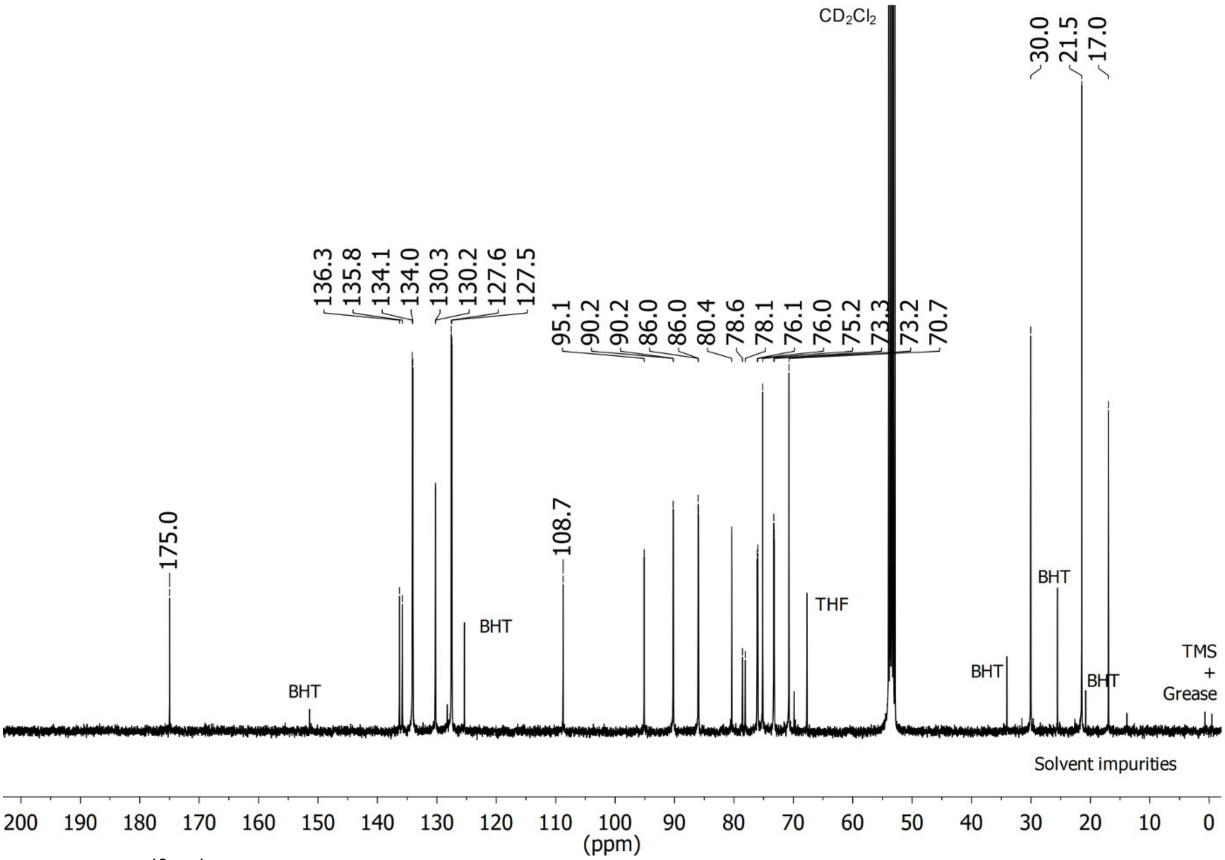

Figure S10. $^{13}C\{^{1}H\}$ NMR spectrum of **[1c(Ru)$_3$]** in CD_2Cl_2 [BHT: dibutylhydroxytoluene (stabiliser in THF)].

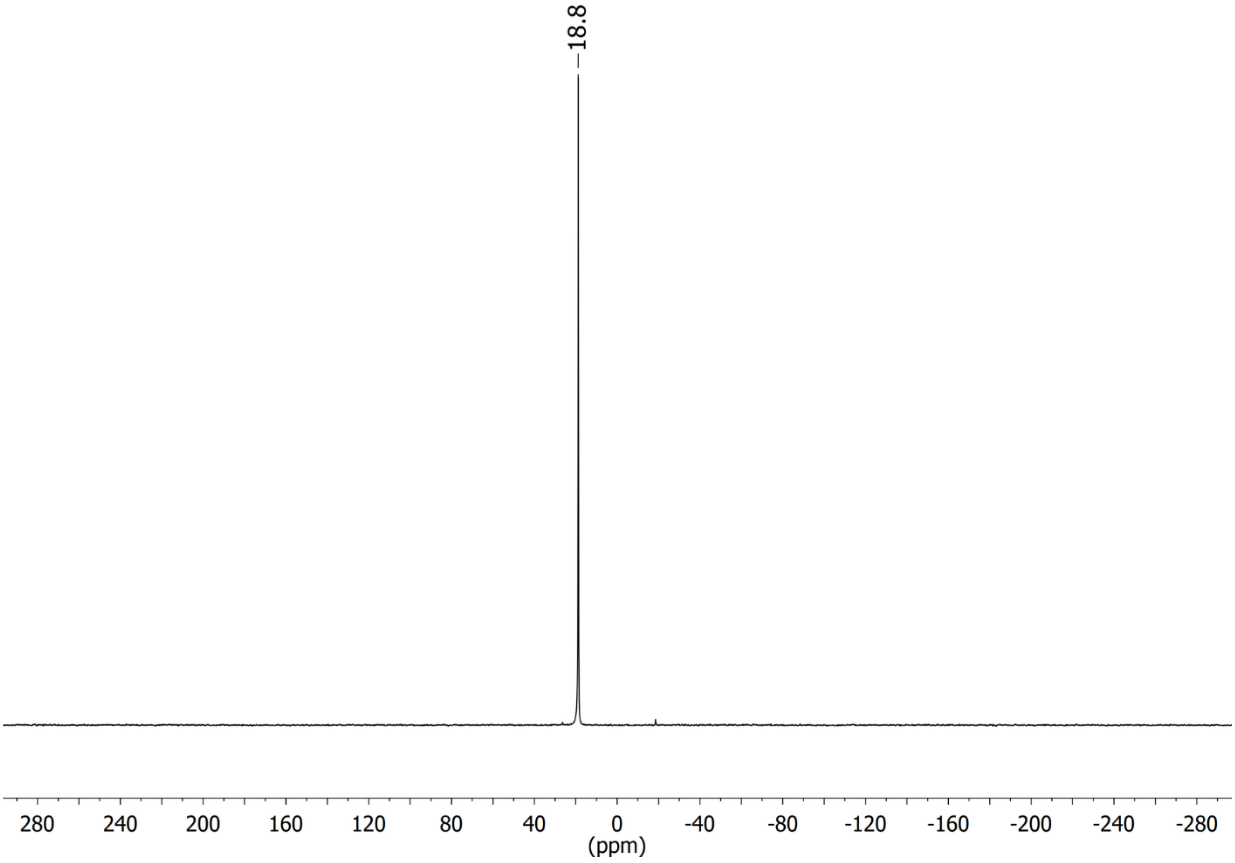

Figure S11. $^{31}P\{^1H\}$ NMR spectrum of **[1c(Ru)$_3$]** in CD_2Cl_2

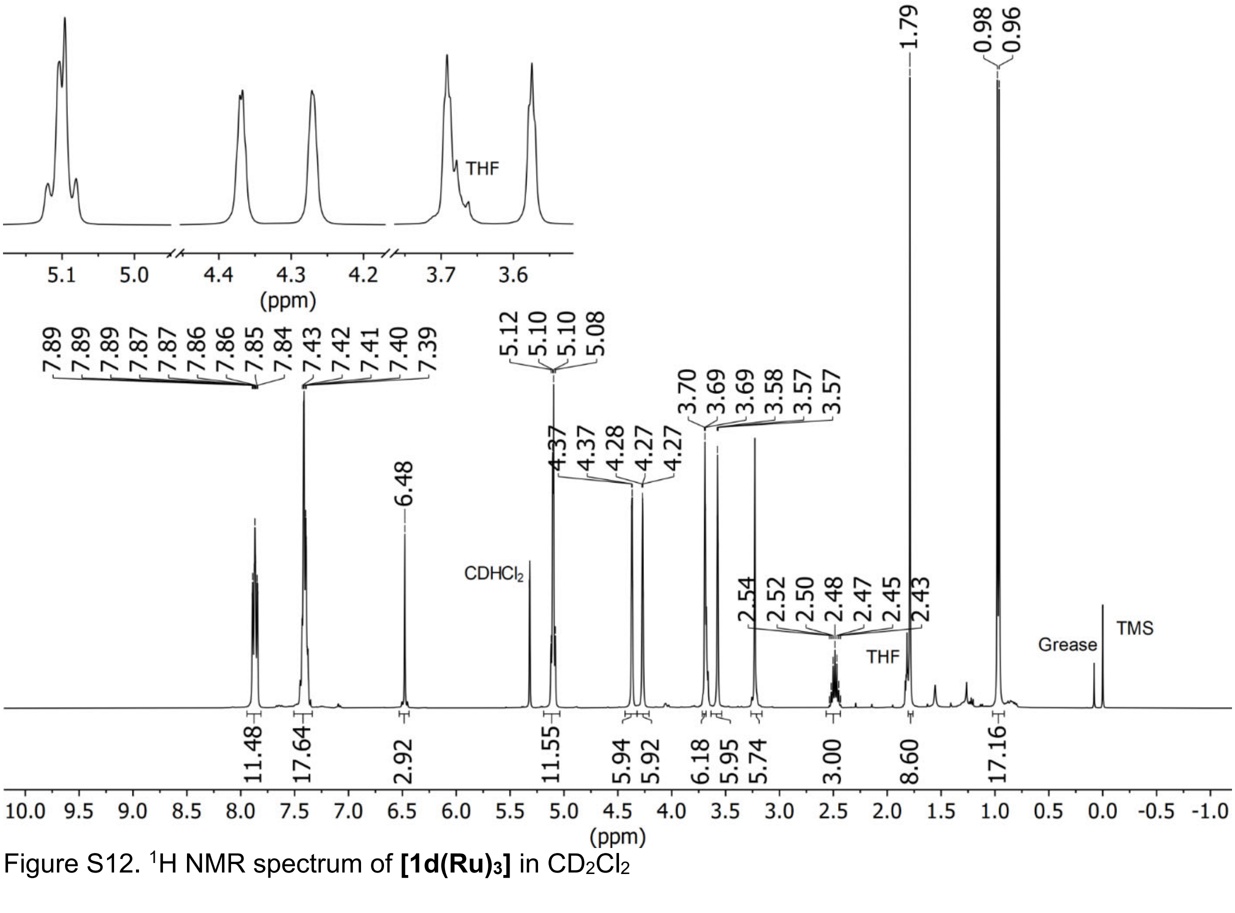

Figure S12. ^1H NMR spectrum of **[1d(Ru)$_3$]** in CD_2Cl_2

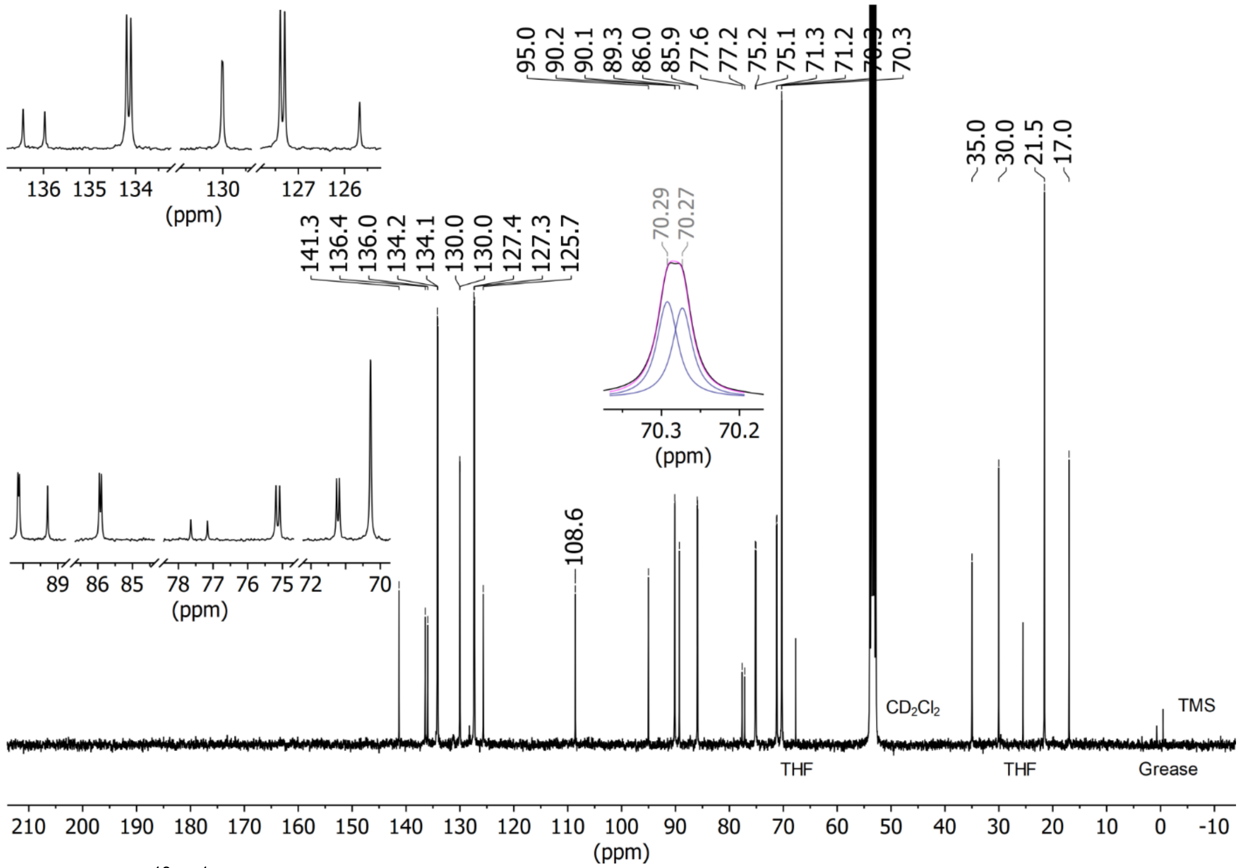

Figure S13. $^{13}C\{^1H\}$ NMR spectrum of **[1d(Ru)3]** in CD_2Cl_2

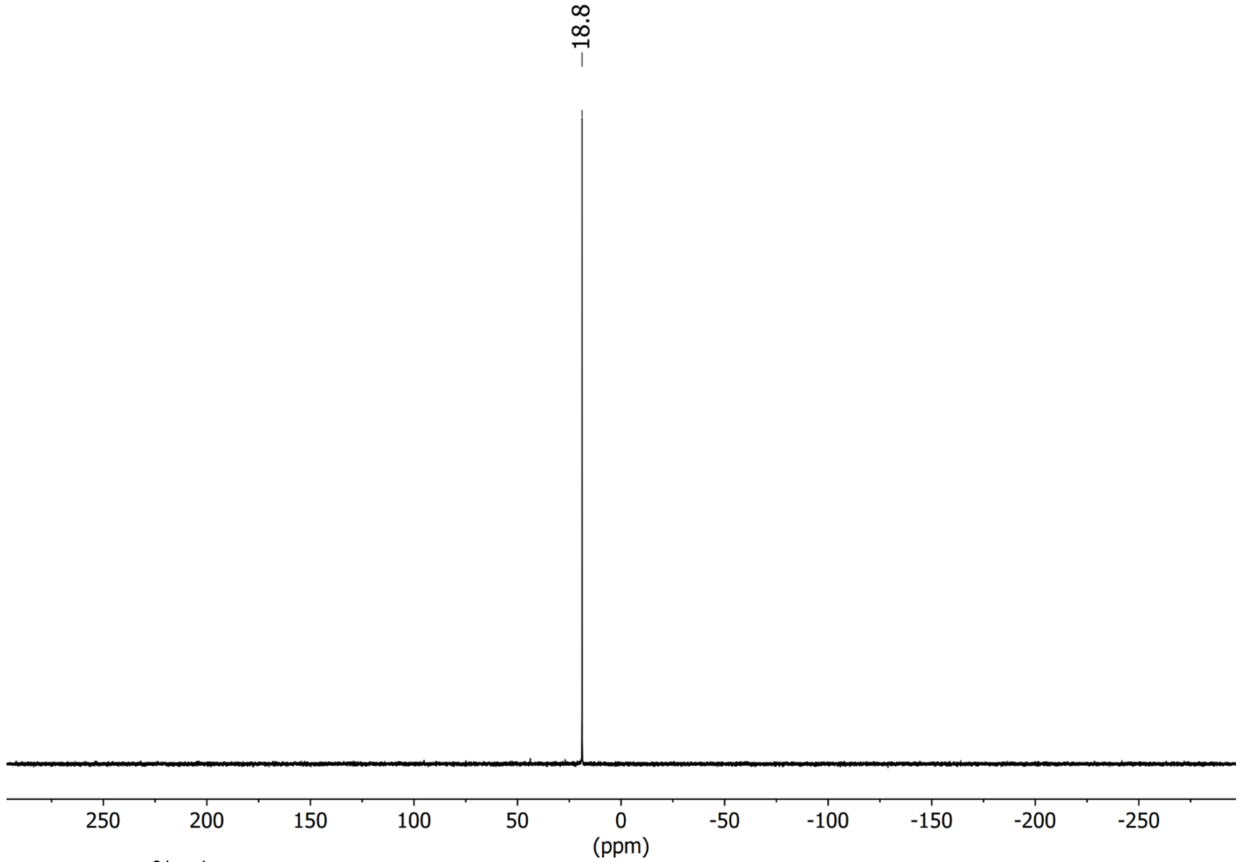

Figure S14. $^{31}P\{^1H\}$ NMR spectrum of **[1d(Ru)3]** in CD_2Cl_2

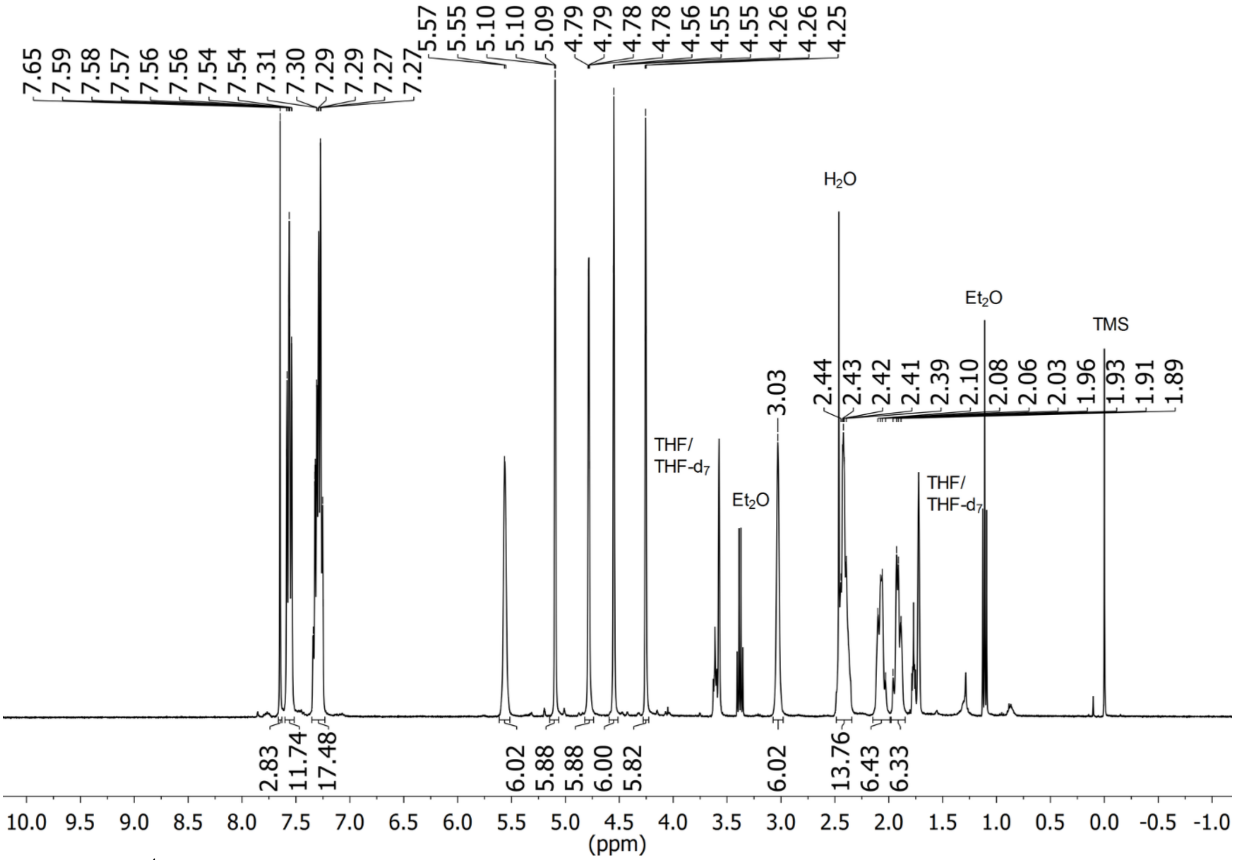

Figure S15. ^{1}H NMR spectrum of **[1a(Rh)3]** in THF-d_8

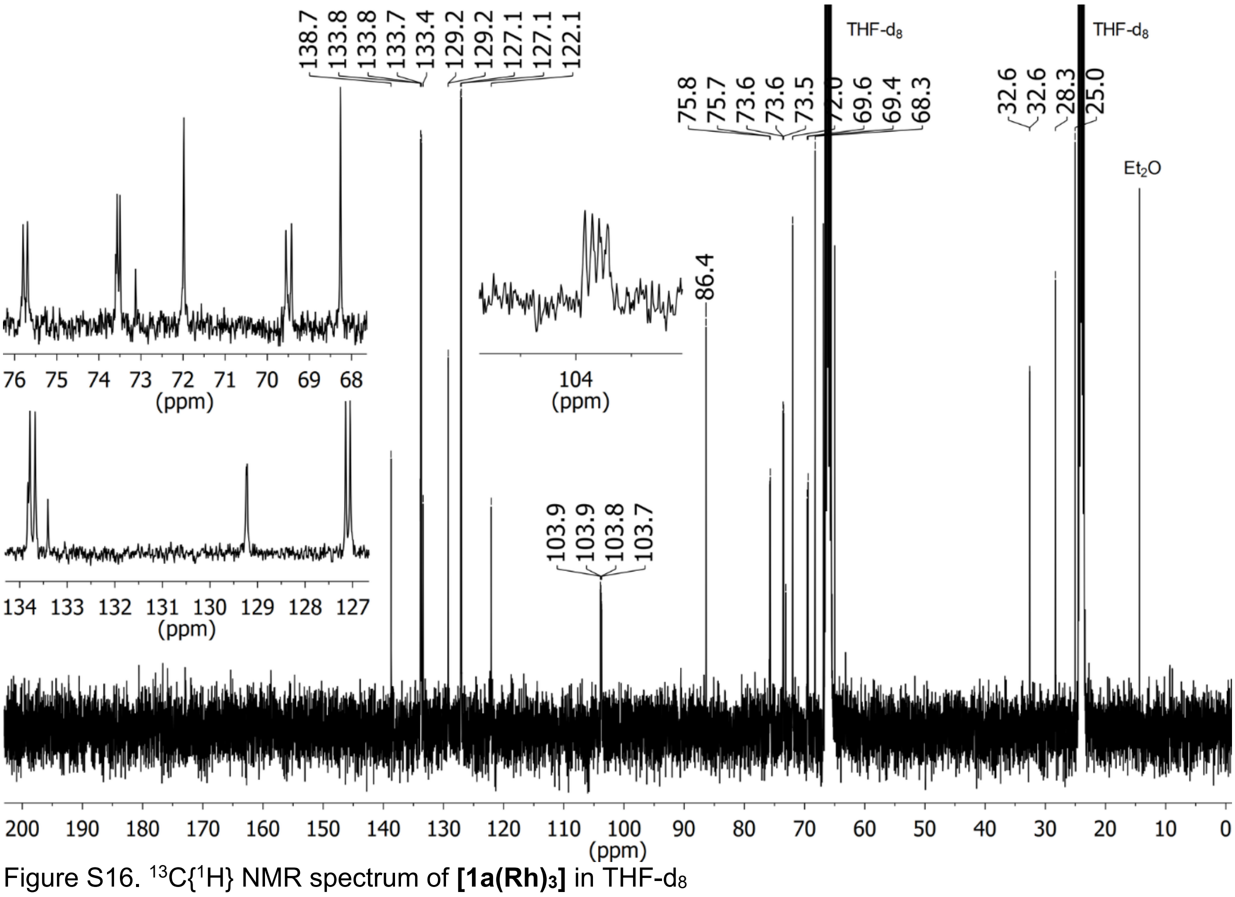

Figure S16. $^{13}C\{^{1}H\}$ NMR spectrum of **[1a(Rh)3]** in THF-d_8

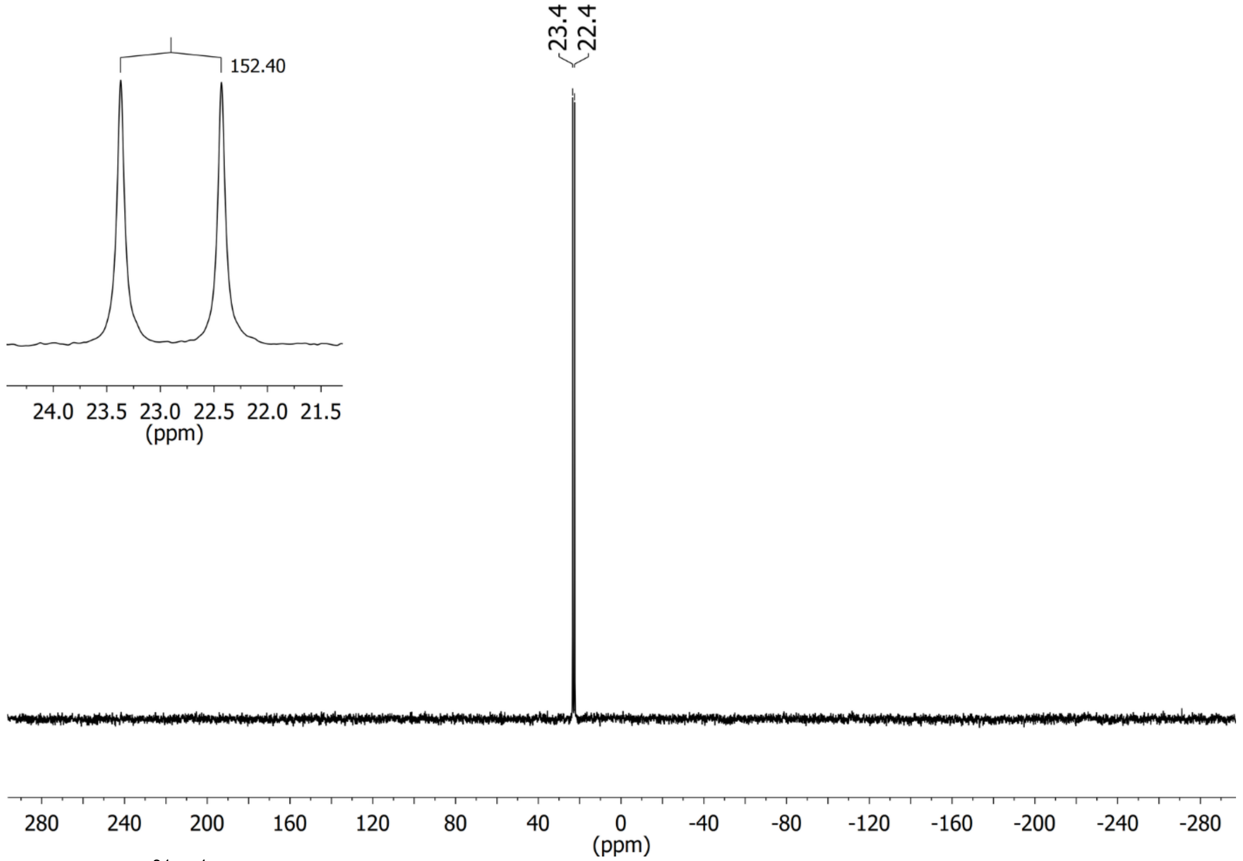

Figure S17. $^{31}P\{^{1}H\}$ NMR spectrum of **[1a(Rh)$_3$]** in THF-d_8

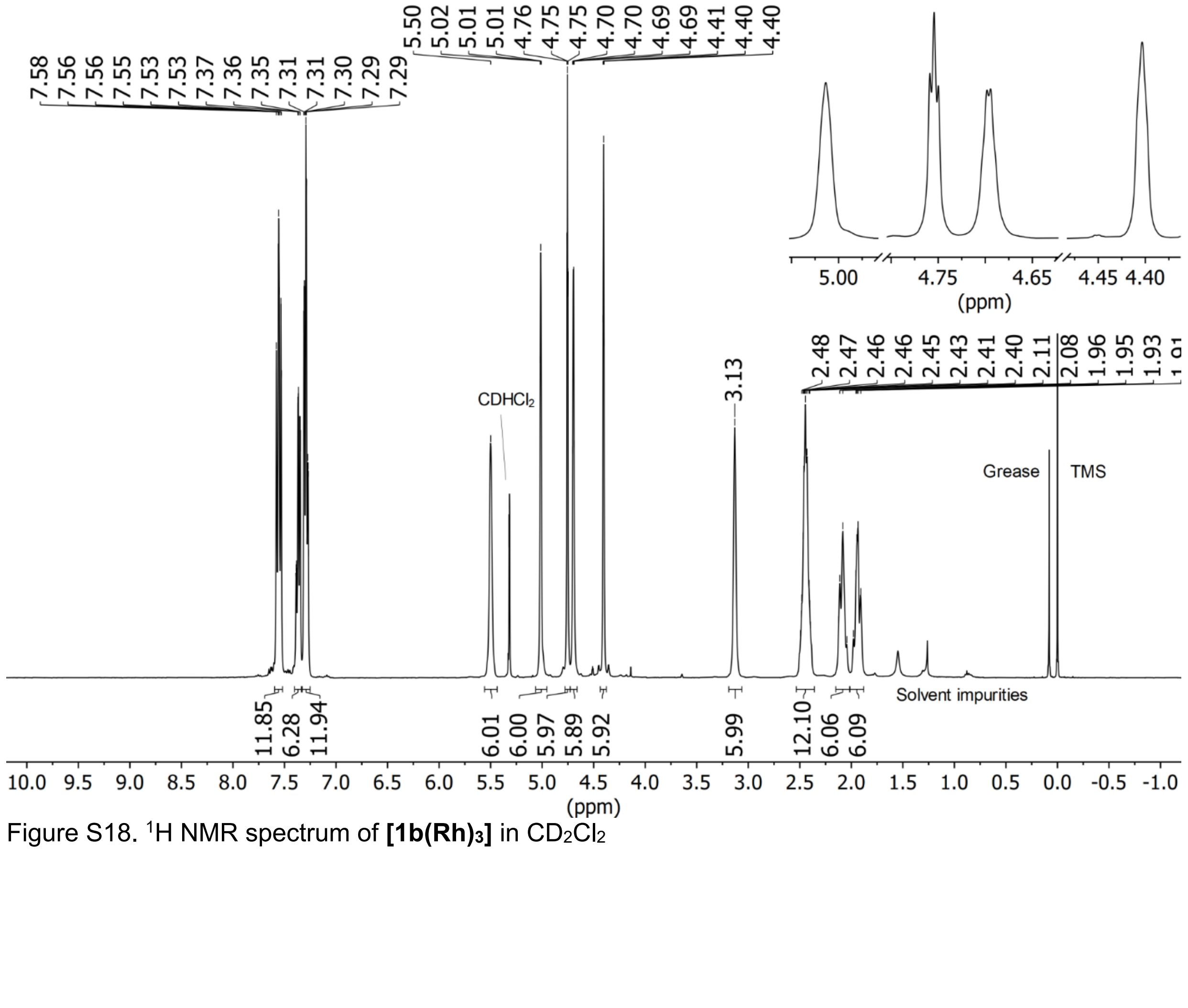

Figure S18. ^{1}H NMR spectrum of **[1b(Rh)$_3$]** in CD_2Cl_2

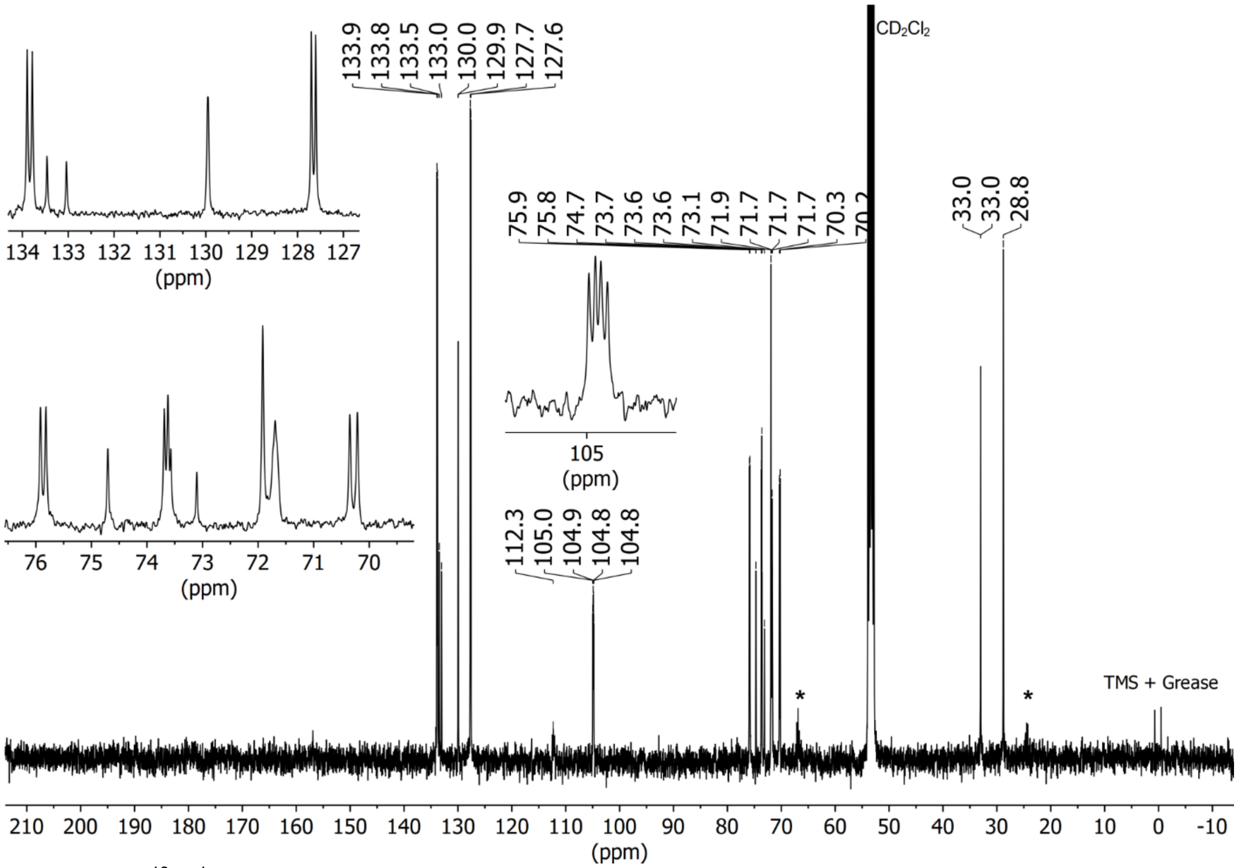

Figure S19. $^{13}C\{^{1}H\}$ NMR spectrum of **[1b(Rh)$_3$]** in CD_2Cl_2

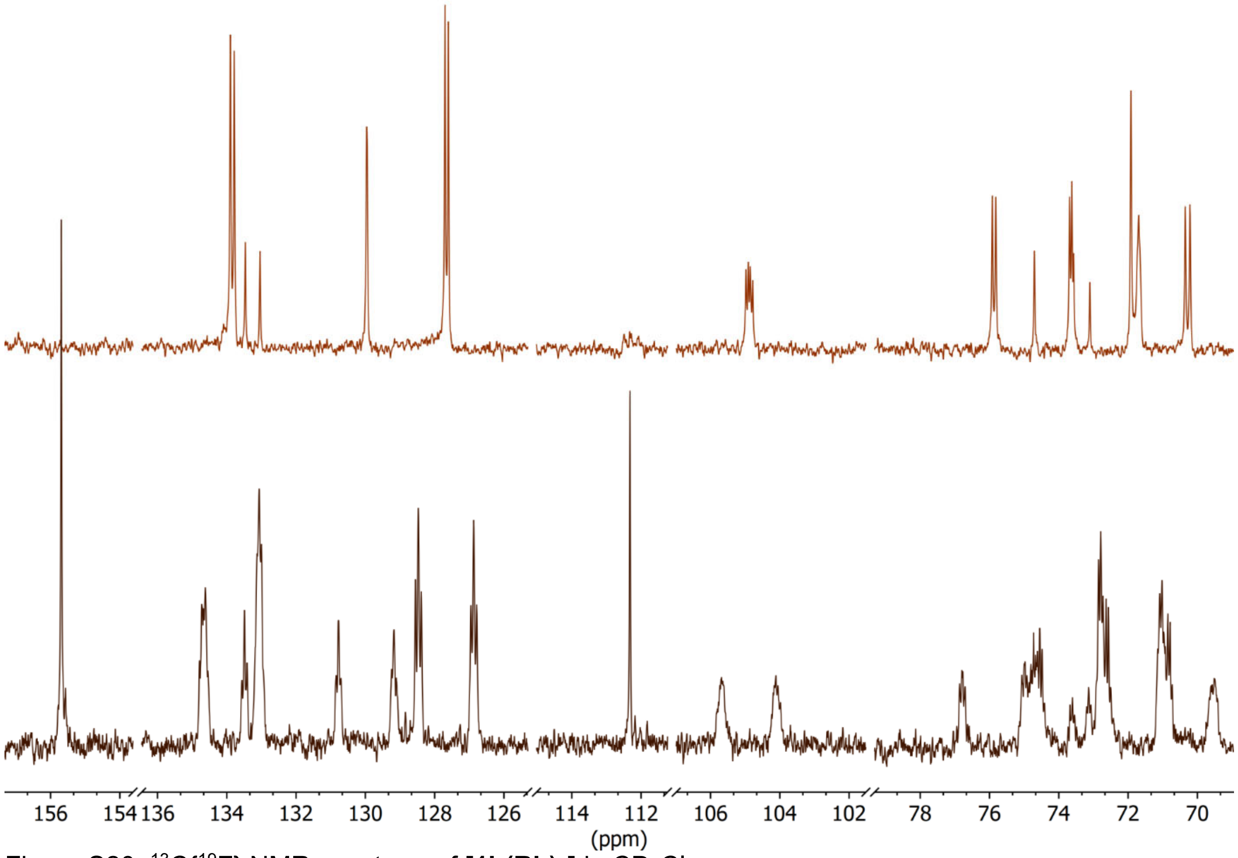

Figure S20. $^{13}C\{^{19}F\}$ NMR spectrum of **[1b(Rh)$_3$]** in CD_2Cl_2

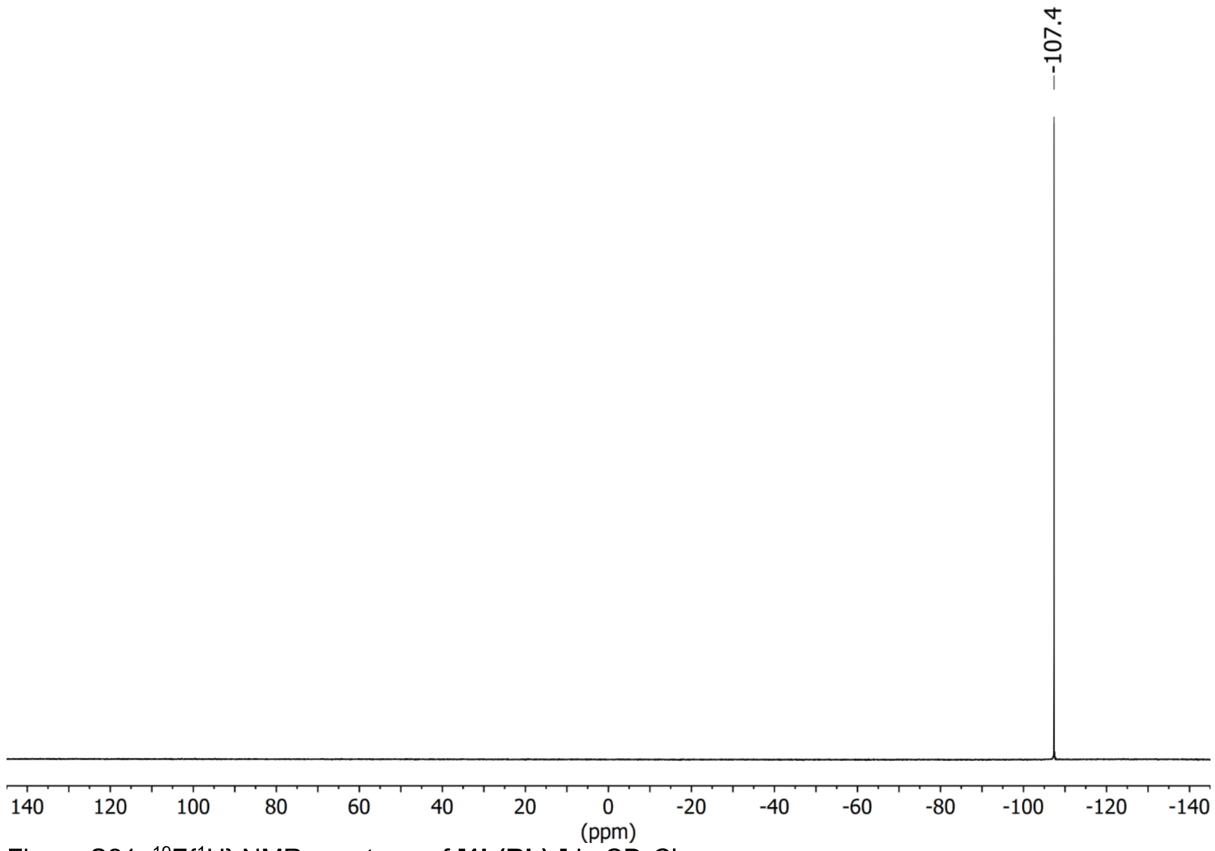

Figure S21. $^{19}F\{^1H\}$ NMR spectrum of **[1b(Rh)$_3$]** in CD_2Cl_2

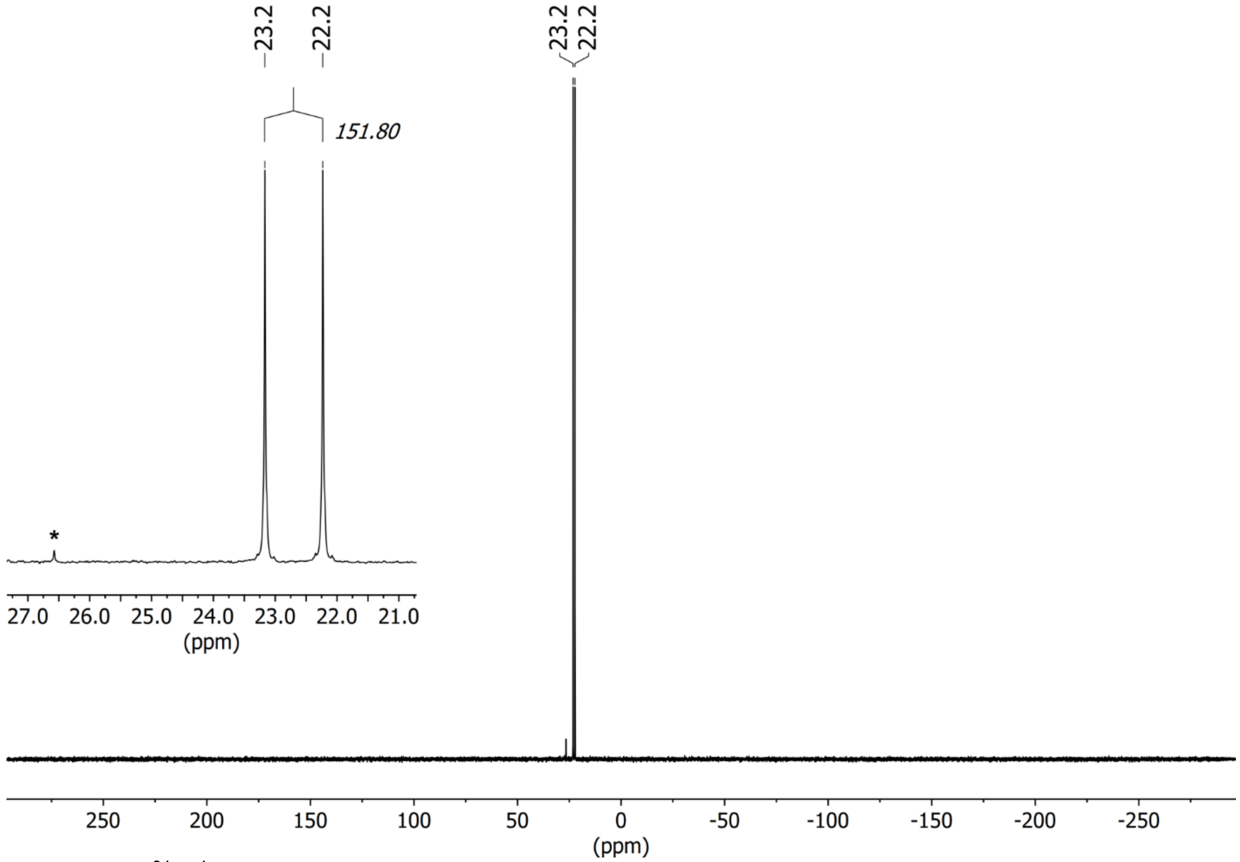

Figure S22. $^{31}P\{^1H\}$ NMR spectrum of **[1b(Rh)$_3$]** in CD_2Cl_2

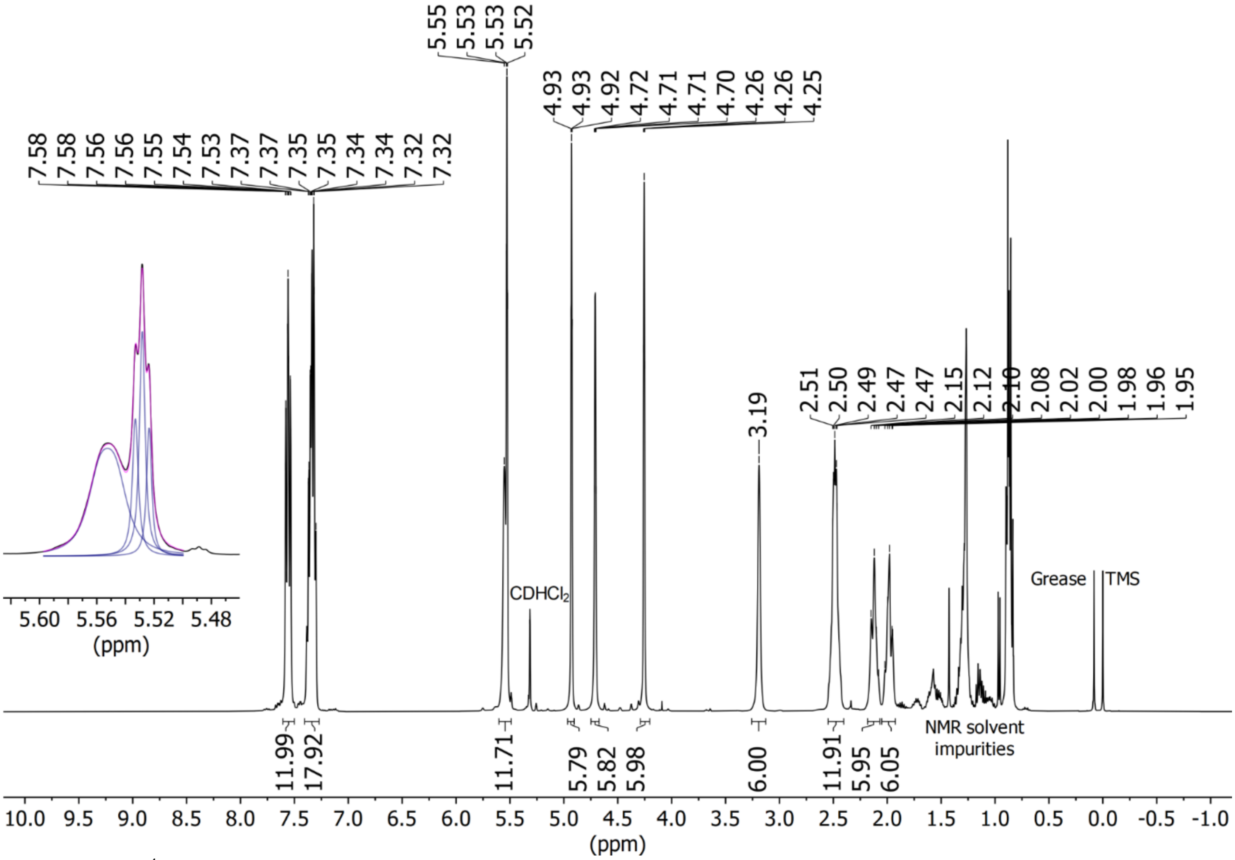

Figure S23. ^{1}H NMR spectrum of **[1c(Rh)$_3$]** in CD_2Cl_2

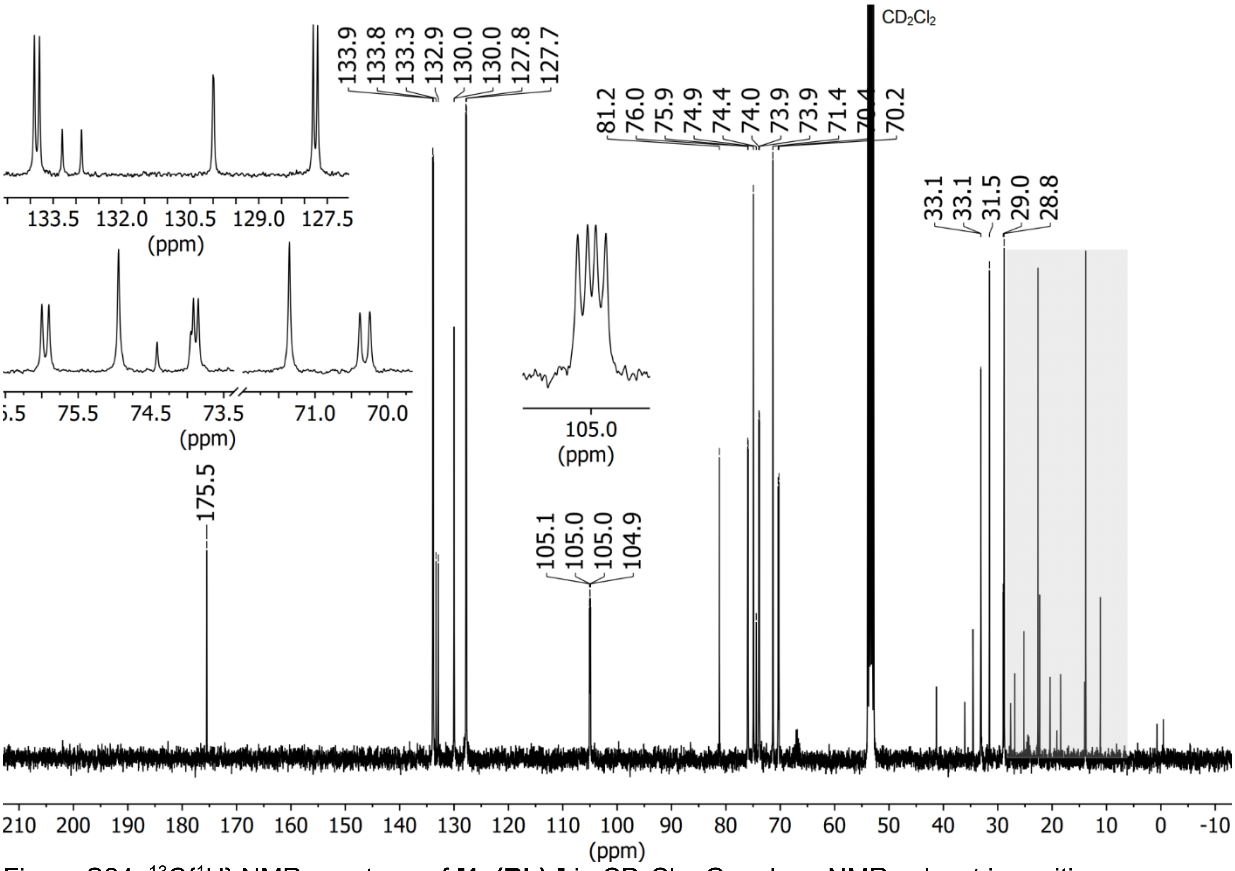

Figure S24. $^{13}C\{^{1}H\}$ NMR spectrum of **[1c(Rh)$_3$]** in CD_2Cl_2.. Grey box: NMR solvent impurities.

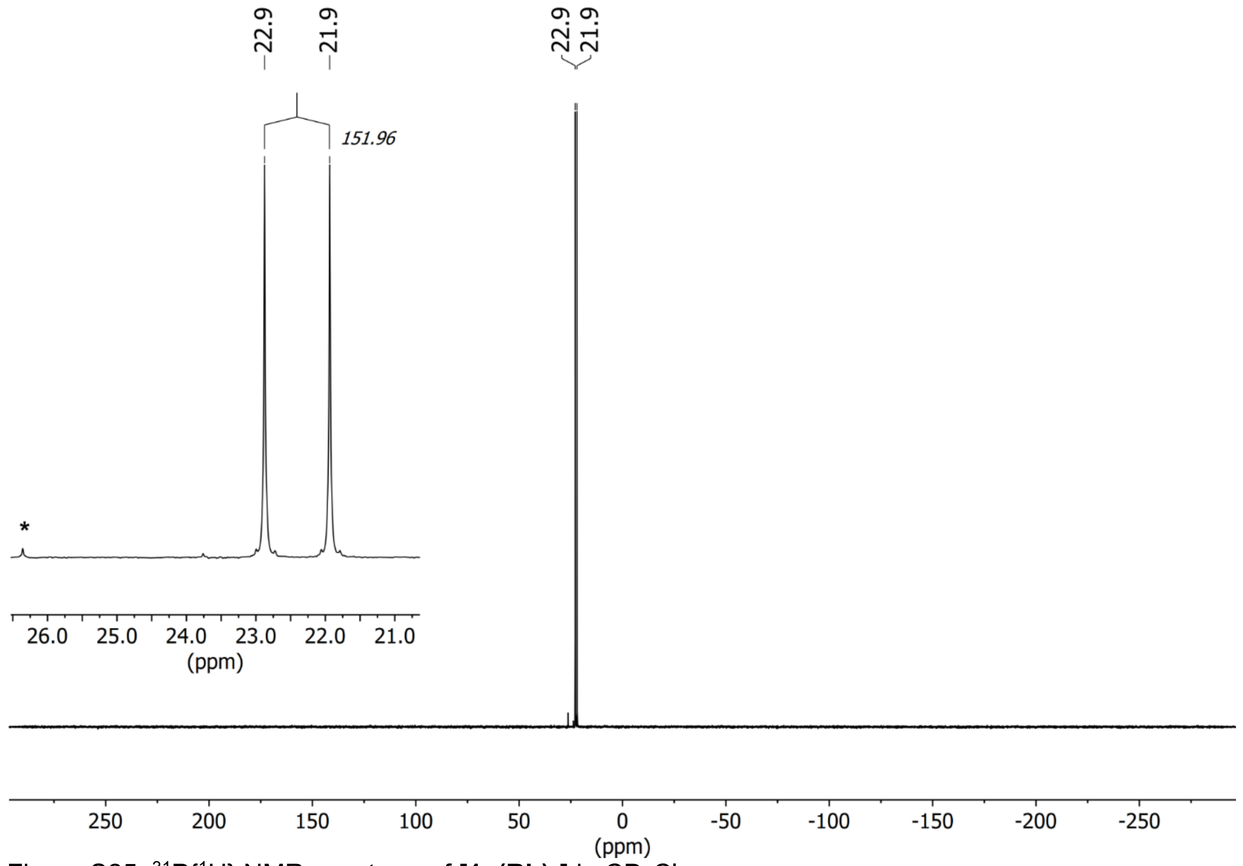

Figure S25. $^{31}P\{^{1}H\}$ NMR spectrum of **[1c(Rh)$_3$]** in CD_2Cl_2

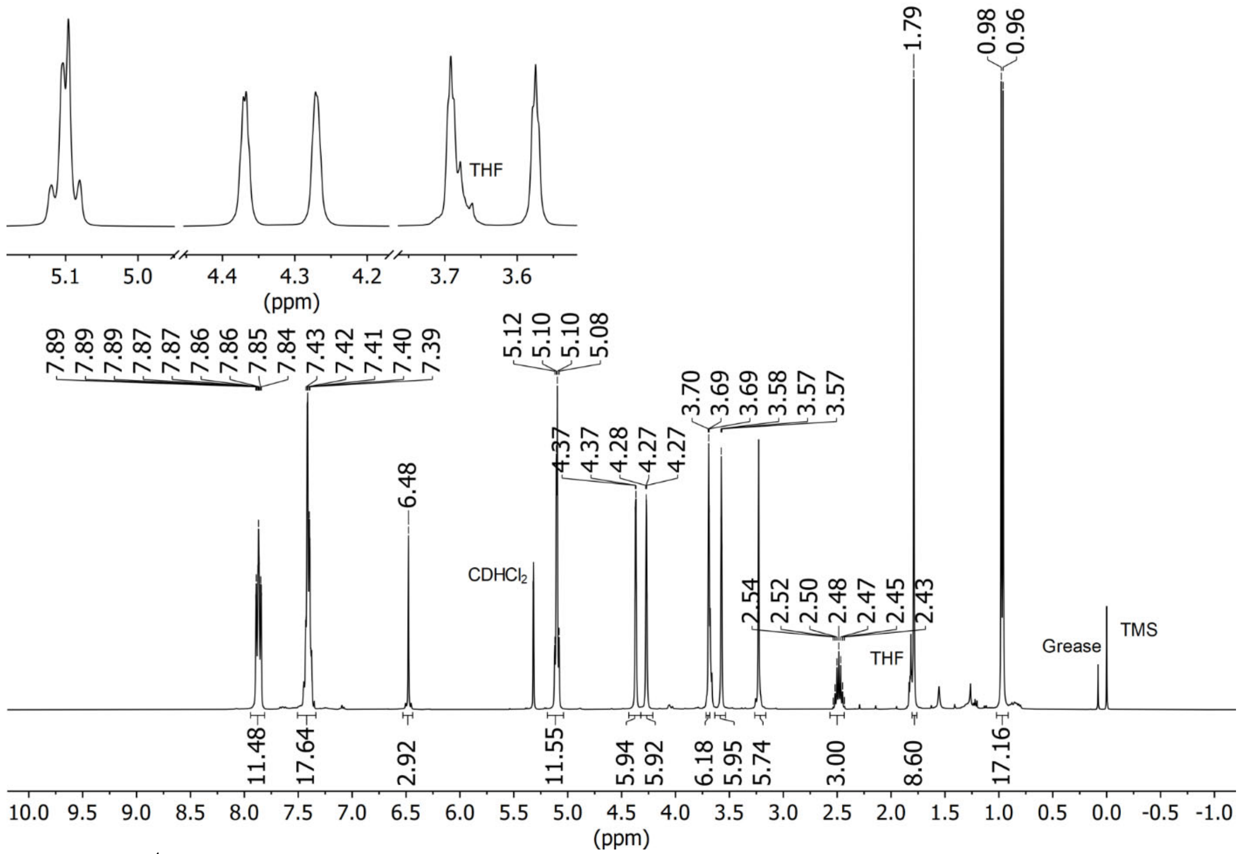

Figure S26. ^{1}H NMR spectrum of **[1d(Rh)$_3$]** in THF-d_8

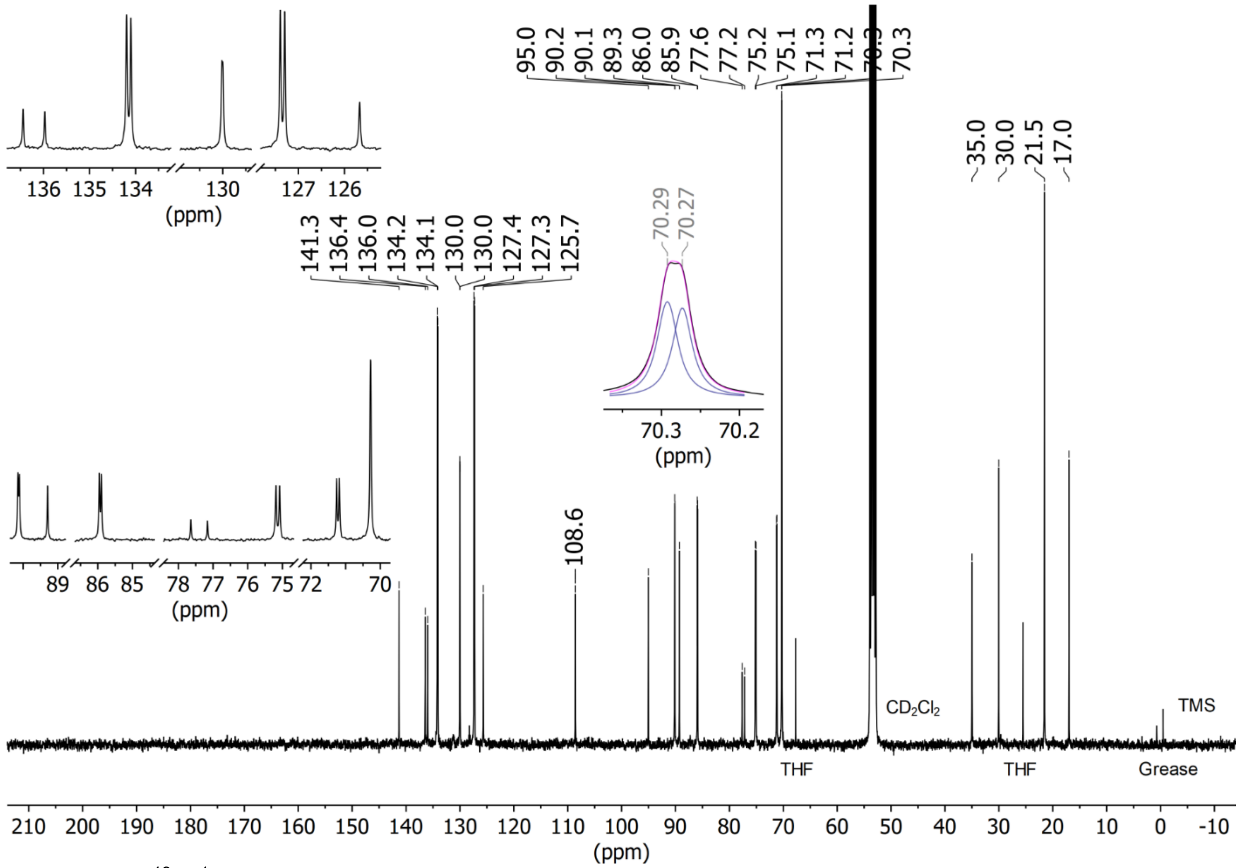

Figure S27. $^{13}C\{^{1}H\}$ NMR spectrum of **[1d(Rh)$_3$]** in THF-d_8

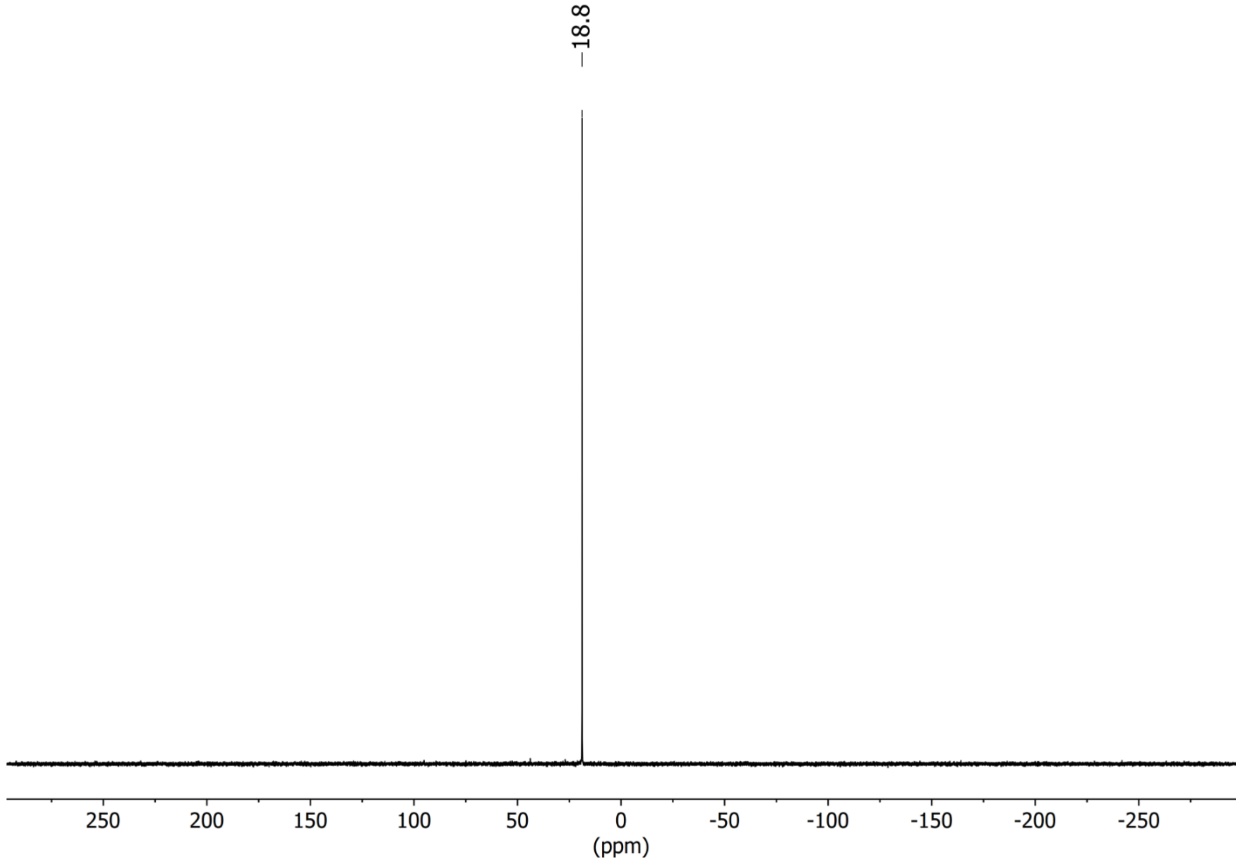

Figure S28. $^{31}P\{^{1}H\}$ NMR spectrum of **[1d(Rh)$_3$]** in THF-d_8

3. Stability of [1a(Ru)$_3$] in CD_2Cl_2 and THF-d$_8$

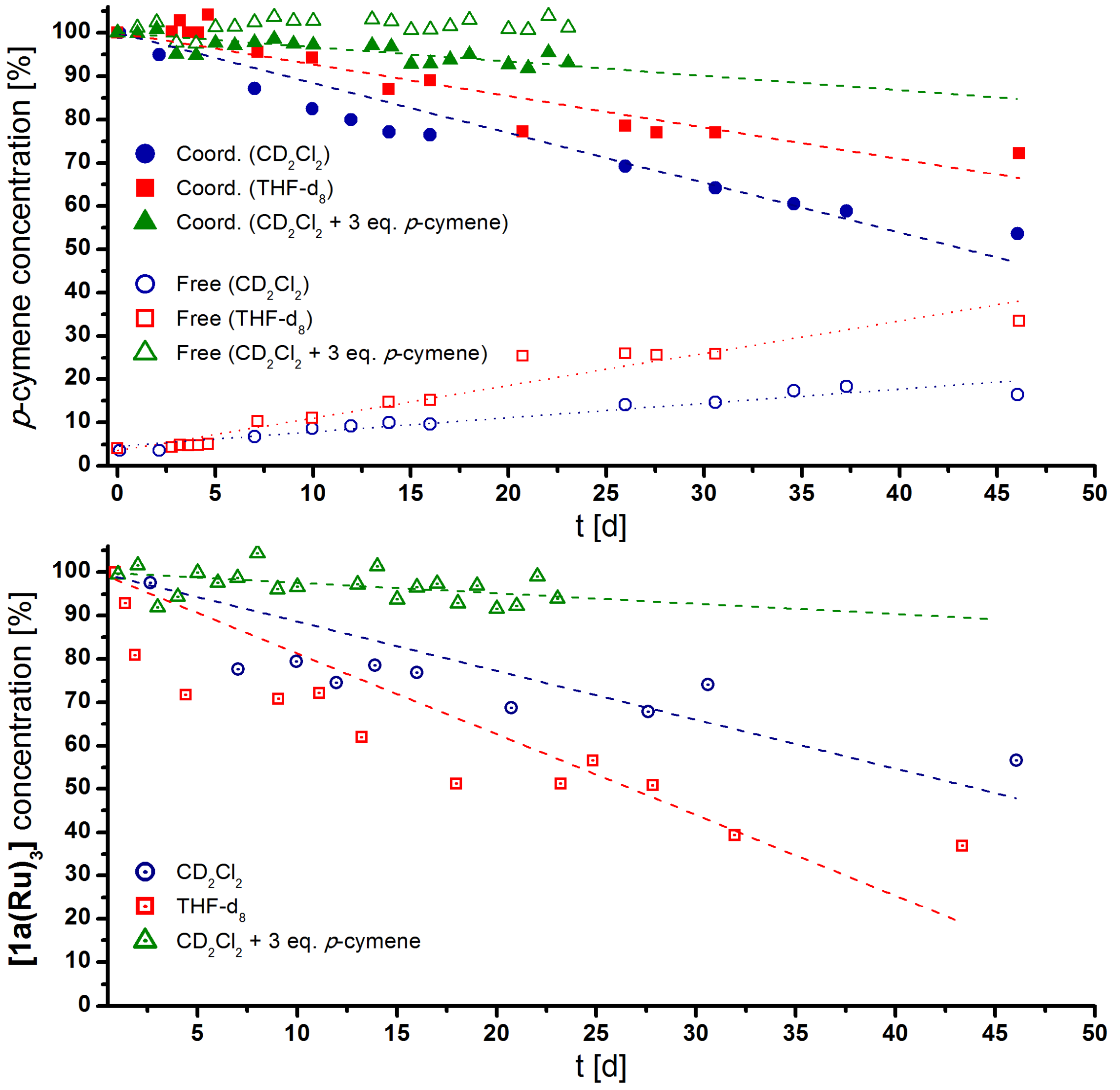

Figure S29. Concentration-over-time graphs for the solvent stability tests of **[1a(Ru)$_3$]** in CD_2Cl_2 (navy), THF-d$_8$ (red), and CD_2Cl_2 in the presence of 3.0 eq. *para*-cymene (green) investigated by means of ^{1}H (top; d = 5 s) and ^{31}P{^{1}H} NMR (bottom; the inverse-gated pulse sequence for quantitative integration was used) spectroscopy. The concentration of coordinated (solid symbols) *vs.* that of free *para*-cymene (hollow symbols) was derived from the integrals of the respective 6 isopropyl methyl protons referenced to an internal standard ([HP(*t*-Bu)$_3$]BF_4, sealed capillary). Similarly, the concentration of the native complex (dot-filled symbols) in ^{31}P{^{1}H} NMR spectroscopy was derived from the integral of the respective signal of **[1a(Ru)$_3$]** referenced to that of the same internal standard. Dashed lines represent linear fits.

Complex **[1a(Ru)$_3$]** was found to slowly lose η^6-coordinated *para*-cymene in CD_2Cl_2 (navy symbols) or THF-d$_8$ (red symbols) solution (Fig. S29). This process was followed with NMR spectroscopy. While, in general, it becomes clear that the loss of *para*-cymene is tied to the decrease in complex concentration, the rate of this process differs depending on what signal is considered. The appearance of free *para*-cymene (hollow symbols in Fig. S29) seems to be faster in THF-d$_8$, while the disappearance of coordinated arene appears faster in CD_2Cl_2. With respect to overall complex concentration determined by ^{31}P NMR spectroscopy, the transformation appears faster in THF-d$_8$. Different mechanisms and transformations might thus take place, depending on the presence of a coordinating (THF-d$_8$) *vs.* a non-coordinating (CD_2Cl_2) solvent. It has, however, to be noted that the ^{31}P

NMR signal of native **[1a(Ru)₃]** broadens considerably and differently in both solvents (Fig. S30), rendering an exact integration impossible. General signal broadening and the rise of very broad signals in the baseline of the recorded spectra is also apparent in ^{1}H NMR spectroscopy (Fig. S30, left), speaking for the formation of (less soluble) oligo- and polymeric products.

The stability of **[1a(Ru)$_3$]** in CD_2Cl_2 can be greatly enhanced by the addition of 3.0 equivalents of *para*-cymene (green symbols in Fig. S29). While a slight decrease in overall complex concentration is noticeable, the rate of transformation is much slower. Accordingly, both ^{1}H and $^{31}P\{^{1}H\}$ NMR spectra show much reduced signal broadening and baseline distortions (Fig. S30).

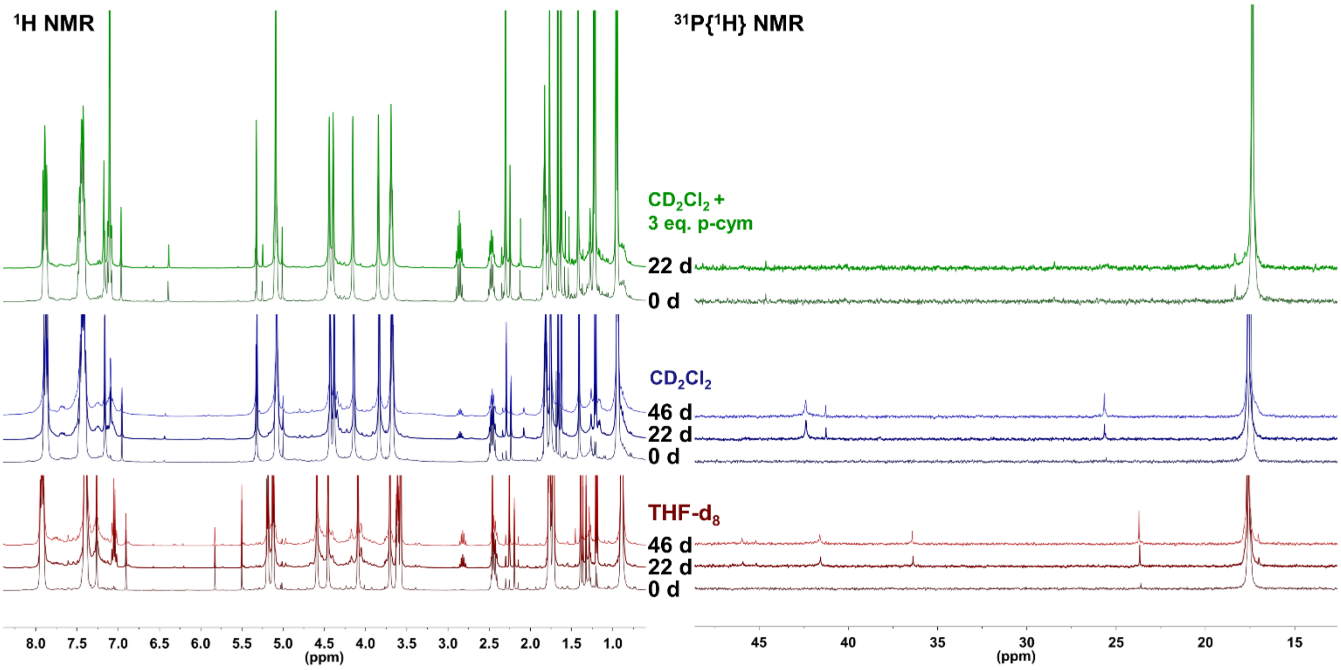

Figure S30. Stacked ^{1}H (left) and $^{31}P\{^{1}H\}$ NMR spectra of **[1a(Ru)$_3$]** in THF-d_8 (red), CD_2Cl_2 (navy), and in the presence of 3.0 equivalents of *para*-cymene (green). ^{1}H NMR spectra have been recorded using a delay d = 5 s. $^{31}P\{^{1}H\}$ NMR spectra have been recorded using the inverse-gated pulse sequence, allowing for quantitative integration of the signals. The spectra recorded after 22 days are highlighted in bold.

4. Single crystal X-ray diffraction analyses

Table S1. Crystallographic data.

	**[(RhCl{1,5-cod})$_3$(C_6H_3{fcPPh$_2$}$_3$)] [1a(Rh)$_3$]**
Empirical formula[a]	$C_{96}H_{93}Cl_3Fe_3P_3Rh_3$ · 1.434 $C_4H_{10}O$ · 1.566 C_4H_8O
Formula weight [g·mol^{-1}]	2141.70
T [K]	130(2)
Crystal system / Space group	Triclinic / $P\overline{1}$
a, b, c [Å]	11.3518(3), 15.4233(4), 26.8182(6)
α, β, γ [°]	89.060(2), 85.478(2), 89.870(2)
V [Å^3]	4680.1(2)
Z	2
ρ_{calc} [g·cm^{-3}]	1.520
Θ_{max} [°]	28.282
F(000)	2202
Reflns collected	132832
Independent reflns	23228
R_1/wR_2 {$I > 2\sigma(I)$}	0.0588 / 0.1057
R_1/wR_2 (all data)	0.0938 / 0.1180
Largest diff. peak/hole [e·Å^{-3}]	1.314 / −0.828

[a] Given as Compound·Solvent for clarity.

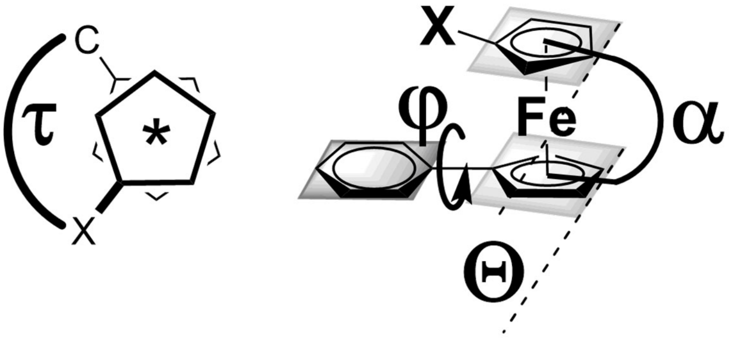

Figure S31. Schematic representation of key structural parameters for the compounds under investigation (τ: torsion about the Cp^X(centroid)···Fe···Cp^C(centroid) axis; ϕ: twist angle between the central arene and the directly bound cyclopentadienyl rings; Θ: angle between the mean planes through the substituted cyclopentadienyl rings; α: angle between Ct^C–Fe–Ct^X). Parameters chosen and named following reference A. Dolmella and G. Bandoli, *Coord. Chem. Rev.*, 2000, **209**, 161.

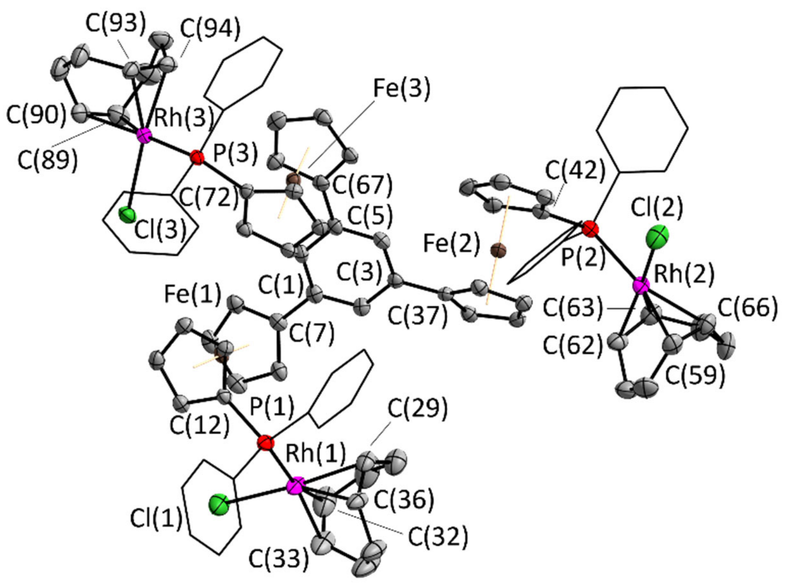

[1a(Rh)$_3$]

Figure S32. Molecular structure of homotrinuclear complex **[1a(Rh)$_3$]** including part of its atom-numbering scheme. Thermal ellipsoids are set at the 50% probability level. P-bound phenyl rings are depicted in wireframe style.

Table S2. Selected bond lengths [Å], distances [Å], and angles [°] of homotrinuclear complex **[1a(Rh)$_3$]**, numbering scheme according to Figure S32, ferrocenylene geometric parameters according to Figure S31.

	[1a(Rh)$_3$]		[1a(Rh)$_3$]
**P(1)–Rh(1) / Rh(1)–Cl(1) /**	2.314(1) / 2.382(1) /	**Rh(1)–C(29/32/33/36)**	2.122(5) / 2.093(5) / 2.211(5) / 2.225(6)
**P(2)–Rh(2) / Rh(2)–Cl(2) /**	2.307(1) / 2.355(1) /	**Rh(2)–C(59/62/63/66)**	2.197(5) / 2.218(5) / 2.117(5) / 2.104(5)
**P(3)–Rh(3) / Rh(3)–Cl(3)**	2.304(1) / 2.370(1)	**Rh(3)–C(89/90/93/94)**	2.205(5) / 2.241(5) / 2.107(5) / 2.118(5)
**Rh(1)···Rh(2) /**	11.5266(7) /	**∡(P(1)–Rh(1)–Cl(1)) /**	91.28(4) /
**Rh(2)···Rh(3) /**	15.9121(7) /	**∡(P(2)–Rh(2)–Cl(2)) /**	88.98(4) /
**Rh(3)···Rh(1)**[a]	11.5422(5)	**∡(P(3)–Rh(3)–Cl(3))**	91.38(4)
**Rh(1)···Fe(1) /**	4.2886(7) /	**α(fc(1)) /**	177.87 /
**Rh(2)···Fe(2) /**	4.4277(7) /	**α(fc(2)) /**	178.05 /
**Rh(3)···Fe(3)**[b]	4.4602(7)	**α(fc(3))**	174.75
**$C^{P}_{ips}(fc_1)$–P(1) /**	1.805(5) /	**Θ(fc(1)) /**	3.61 /
**$C^{P}_{ips}(fc_2)$–P(2) /**	1.804(5) /	**Θ(fc(2)) /**	2.89 /
**$C^{P}_{ips}(fc_3)$–P(3)**[c]	1.800(5)	**Θ(fc(3))**	5.93
**$C^{C}_{ips}(fc_1)$–C^{Link}(1) /**	1.478(6) /	**τ(fc(1)) /**	79.19 /
**$C^{C}_{ips}(fc_2)$–C^{Link}(2) /**	1.484(6) /	**τ(fc(2)) /**	147.16 /
**$C^{C}_{ips}(fc_3)$–C^{Link}(3)**[d]	1.476(6)	**τ(fc(3))**	146.31
**Ct^{C}(1)–Fe(1) /**	1.654 /	**ϕ(fc(1)) /**	29.69 /
**Ct^{C}(2)–Fe(2) /**	1.642 /	**ϕ(fc(2)) /**	35.22 /
**Ct^{C}(3)–Fe(3)**[e]	1.646	**ϕ(fc(3))**	8.97
**Ct^{P}(1)–Fe(1) /**	1.652 /		
**Ct^{P}(2)–Fe(2) /**	1.640 /		
**Ct^{P}(3)–Fe(3)**[f]	1.647		

[a] Intramolecular metal-metal distances; the shortest intermolecular Rh-Rh distance is 6.1288(6) Å; [b] Intramolecular separation between coordinated metal and the corresponding iron centre; [c] $C^{P}_{ips}(fc_n)$ corresponds to the respective *ipso* carbon atom of the P-substituted cyclopentadienyl ring (**[1a(Rh)$_3$]**: C(12), C(42), C(72)); [d] C^{Link}(n) refers to the C atom to which the C-substituted cyclopentadienyl is bound ((**[1a(Rh)$_3$]**: C(1), C(3), C(5)); [e] Ct^{C} denotes the calculated centre of gravity of the carbon-substituted cyclopentadienyl ring; [f] Ct^{P} denotes the calculated centre of gravity of the P-substituted cyclopentadienyl ring.

Electrochemistry of complexes [1a-d(Ru)$_3$] and [1a-d(Rh)$_3$]

The complexes **[1a-d(Ru)$_3$]** and **[1a-d(Rh)$_3$]** have been analysed in both (nBu$_4$N)BF$_4$ and (nBu$_4$N)BAr$^F_4$ as supporting electrolytes in CH$_2$Cl$_2$, respectively; Pt wire counter electrode, glassy carbon (GC) working electrode; Ag/Ag$^+$ reference electrode with external reference FcH/[FcH]$^+$ or Me$_{10}$fc/[Me$_{10}$fc]$^+$ added to one sample of a measurement series in excess; Ohmic drop has not been compensated.

4.1. General / Summary

All complexes are electrochemically active; their redox behaviour is more "well-behaved", i.e., fully (quasi)reversible and thus amenable to full redox control in potential applications, in the less strongly coordinating electrolyte with the BAr$^F_4$ anion. This supporting electrolyte (SE) allows to nicely separate the three iron-centred oxidations which are, under these conditions, mostly reversible but, especially for the third oxidation for some of the ligand systems that feature a substituted benzene or a heterocyclic core, already show signs of irreversibility. The system with the least complications in terms of irreversible oxidation steps, in all cases, is the C$_6$H$_3$-based ligand. All other systems show deviations from electrochemically ideal behaviour in cyclic voltammetry, which makes them less well suited for potential applications in redox-switchable catalysis

For the Rh complexes, a fourth, likely Rh-centred oxidation can be seen for the less electron-withdrawing ligands, but for the C$_6$F$_3$ and C$_3$N$_3$ core ligands, the fourth event is outside the window of electrochemical stability of the supporting electrolyte

For the Ru complexes, two more, likely Ru-centred oxidations become visible in the BAr$_4^-$-based SE. None of the Ru/Rh-centred oxidations appear to be electrochemically reversible.

4.2. Rhodium complexes

The electrochemical characterisation of **[1a(Rh)$_3$]** (Fig. 33) showed a more complex behaviour, giving rise to metal-centred oxidations owing to the redox-active rhodium(I) centres. The peak potentials for the Rh^I/Rh^{II} couple (751 mV [BF$_4^-$], 854 mV [BAr$^F_4$$^-$]) are significantly higher and the oxidations less reversible than for a related *P*-ferrocenophane-derived chlorido(cyclooctadiene)rhodium(I) complex (E^0 = 390 mV *vs*. FcH/[FcH]$^+$) by Breher and co-workers [A. Feyrer, M. K. Armbruster, K. Fink and F. Breher, *Chem. Eur. J.*, 2017, **23**, 7402]. This reduced reversibility might be tied to the fact that the oxidation of the rhodium centre will most likely generate a quadruply charged cation. In the BF$_4^-$-based SE, an irreversible first oxidation complicates the electrochemistry of **[1a(Rh)$_3$]** even more. Further oxidation, even though seemingly limited to the tris(ferrocenyl)benzene core, gives rise to cathodically shifted reduction events (dotted *vs*. solid red and pink lines, Fig. 33 left) again. This is not the case in the BAr$^F_4$$^-$-based SE (Fig. 33, right), although the rhodium-centred oxidation is less reversible and does not affect the tris(ferrocenyl)benzene-based redox events.

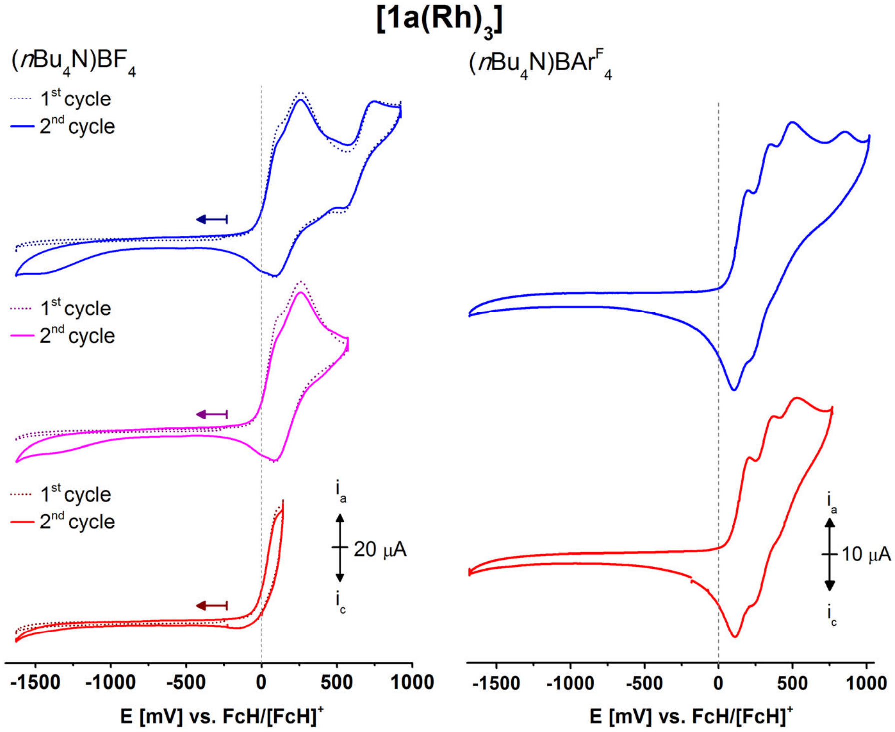

Figure S33. Cyclic voltammograms of rhodium(I) complex **[1a(Rh)$_3$]** in the BF_4- (left) and $BAr^F{}_4$-based (right) supporting electrolytes. For all voltammograms, the second of three consecutively measured cycles is shown if not indicated otherwise (dotted lines). The scan rate was set to 100 mV·s^{-1}, and scans have been carried out in anodic direction if not indicated otherwise by the arrows, signifying starting potential and initial cathodic scan direction.

BF_4-based Supporting Electrolyte

Table S3. Redox E° and peak potentials $E^{ox/red}$ [mV] of the tris(1-diphenylphosphanyl-1'-ferrocenylene)arene rhodium(I) complexes **[1a-d(Rh)$_3$]** in 0.1 M (nBu$_4$N)BF_4/CH_2Cl_2 determined by cyclic voltammetry.[a]

Complex	E^{ox}_{fc} / E^{red}_{fc} [b]	E^{ox}_{Rh} / E^{red}_{Rh} [c]	E^{ox}_{fl} / E^{red}_{fl} [d]
**[1a(Rh)$_3$] (C_6H_3)**	112, 260 / 90 [h]	751 / 537	- / −1400
**[1b(Rh)$_3$] (C_6F_3)**	175, 350 / 130, 270 [i]	700 / 640 [j]	−600[k] / −685
**[1c(Rh)$_3$] (C_3N_3)**	265, 480 / 355	710[l] / -	−640 / −560
**[1d(Rh)$_3$] ($C_6(CH_2)_3$)**	125[m] /20[m]	645[l] / 550	−660 / −560, −1400

[a] Potentials *vs*. the FcH/[FcH]$^+$ couple at a glassy carbon working electrode (scan rate 100 mV·s^{-1}), the splitting between the anodic (oxidation) and cathodic (reduction) peaks given in brackets; [b] Determined in an anhydrous 0.1 mol·L^{-1} (nBu$_4$N)BF_4/CH_2Cl_2 solution; [c] Peak potentials of the Rh/Ru-centred oxidation and reduction events in anhydrous 0.1 mol·L^{-1} (nBu$_4$N)BF_4/CH_2Cl_2 solution; [d] Peak potentials of a second, cathodically shifted reduction and a concomitant oxidation event observed after a first redox cycle of the analyte in anhydrous 0.1 mol·L^{-1} (nBu$_4$N)BF_4/CH_2Cl_2 solution; [h] The Fe-centred oxidations being non-reversible, the peak potentials for both oxidations and the corresponding reduction events are given; [l] Only partially resolved; [j] Only fully visible upon first scan, then only apparent as shoulder; [k] Linked to previous oxidation of rhodium centres as absent in CV with vertex potential below E^{ox}_{Rh}. [l] Only distinctly visible in first scan; [m] Somewhat broadened, likely more than one individual event.

At slow scan speeds (20 mV·s^{-1}), the oxidations in **[1b(Rh)$_3$]** become fully irreversible; no reduction event is seen.

For **[1c(Rh)$_3$]** in the BF_4-based SE, even the first oxidation is irreversible and already linked to a minor cathodically shifted reduction event, indicating the presence of an EC mechanism (electrochemical oxidation followed by chemical reaction). The small shoulder in the first anodic scan of **[1c(Rh)$_3$]** in the BF_4-based SE is also seen in **[1b(Rh)$_3$]** but vanishes upon further cycling; unlike for **[1a(Rh)$_3$]**, it is thus difficult to determine whether there is rhodium-based oxidation event within the scanned potential window.

BAr^F_4-based Supporting Electrolyte

Table S4. Redox E° and peak potentials $E^{ox/red}$ [mV] of the tris(1-diphenylphosphanyl-1'-ferrocenylene)arene rhodium(I) complexes **[1a-d(Rh)₃]** in 0.1 M ($^nBu_4N)BAr^F_4/CH_2Cl_2$ determined by cyclic voltammetry (BAr^F_4 = [B{3,5-$(CF_3)_2C_6H_3$}$_4$]).[a]

	E°₁ (ΔEp)	E°₂ (ΔEp)	E°₃ (ΔEp)	ΔE°1/2 / ΔE°2/3 [b]	E^{ox}_{Rh} / E^{red}_{Rh} [c]
**[1a(Rh)₃] (C_6H_3)**	158 (99)	305 (134)	466 (126)	147 / 161	854 / - [d]
**[1b(Rh)₃] (C_6F_3)**	190 (86)	321 (106)	486 (116)	131 / 164	- [e]
**[1c(Rh)₃] (C_3N_3)**	283 (86)	450 (n.r.) [f]	682 (211)	167 / 230	- [e]
**[1d(Rh)₃] ($C_6(CH_2)_3$)**	116 (93)	215 (128)	390 (n.r.) [g]	99 / n.d.	E°₄ = 606 (90), E°₅ = 998 (300) [h]

[a] Potentials *vs*. the FcH/[FcH]⁺ couple at a glassy carbon working electrode (scan rate 100 mV·s⁻¹), the splitting between the anodic (oxidation) and cathodic (reduction) peaks given in brackets where applicable; [b] Potential-to-potential separation of the first and second/second and third redox process, respectively; [c] Peak potentials of the Rh-centred oxidation and reduction events; [d] No reduction event was observed; [e] No additional redox events were observed within the electrochemical stability window of the supporting electrolyte; [f] The reduction event was not resolved (n.r.) and the redox potential for the process was thus estimated from the cyclic voltammogram; [g] Estimated from shoulder in square-wave voltammogram, reduction not resolved; [h] Two reversible, likely Rh-centred redox processes were identified and are given with redox potentials and peak separations ΔE_p in round brackets ([mV])

The third, likely still Fe-centred waves for the Rh complexes in the BAr^F_4/CH_2Cl_2 SE are usually quite broad, indicating deviations from ideal redox processes (i.e., triply oxidised forms of these complexes either not very stable and/or deposit on the electrode).
Square-wave voltammetry at 100 mV·s⁻¹ has been run for **[1c(Rh)₃]**, **[1b(Rh)₃]** and **[1d(Rh)₃]**. The third waves for the Rh complexes in the BAr^F_4/CH_2Cl_2 SE are usually quite broad.

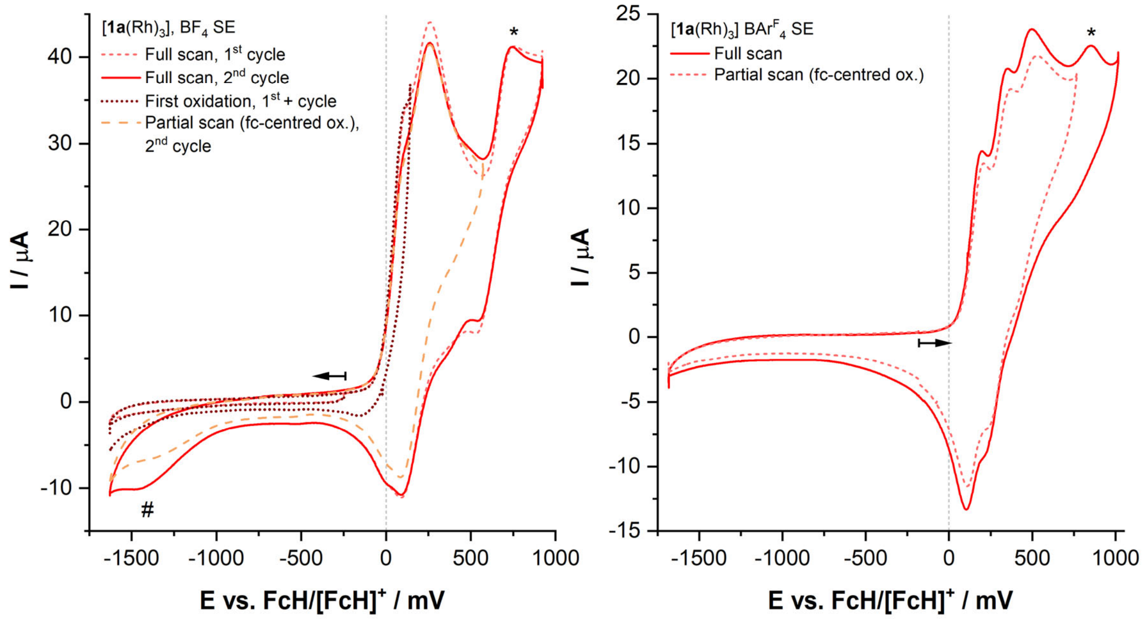

Figure S34. Cyclic voltammograms of C_6H_3 ligand core rhodium complex **[1a(Rh)₃]** in BF_4- (left) and BAr^F_4-based (right) supporting electrolytes. The second of three consecutively measured cycles are shown if not indicated otherwise. The scan rate was set to 100 mV·s⁻¹; scan direction and initial potential indicated by the arrow. Presumably Rh-centred oxidation events are marked with an asterisk (*); cathodic reduction events that are linked to prior oxidation of metal centres are indicated with the hashtag symbol (#). In the case of the BAr^F_4-based supporting electrolyte, application of higher anodic potentials led to the onset of electrolyte decomposition and is thus not shown.

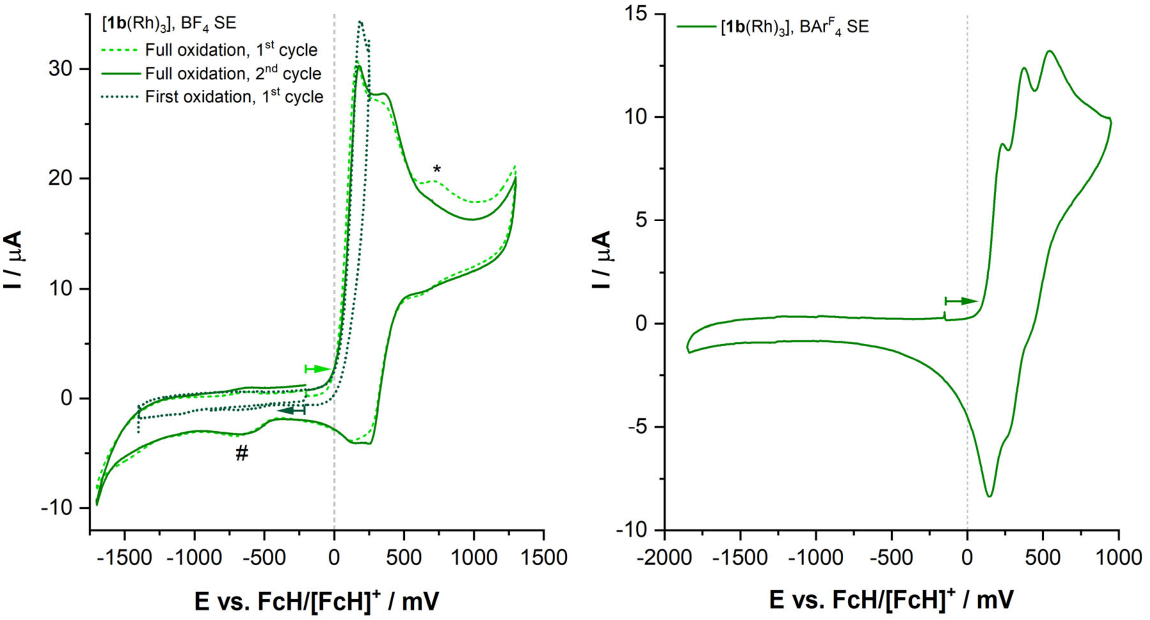

Figure S35. Cyclic voltammograms of C_6F_3 ligand core rhodium complex **[1b(Rh)3]** in BF_4- (left) and BAr^F_4-based (right) supporting electrolytes. The second of three consecutively measured cycles are shown if not indicated otherwise. The scan rate was set to 100 mV·s^{-1}; scan direction and initial potential indicated by the arrow. Presumably Rh-centred oxidation events are marked with an asterisk (*); cathodic reduction events that are linked to prior oxidation of metal centres are indicated with the hashtag symbol (#). In the case of the BAr^F_4-based supporting electrolyte, application of higher anodic potentials led to the onset of electrolyte decomposition and is thus not shown.

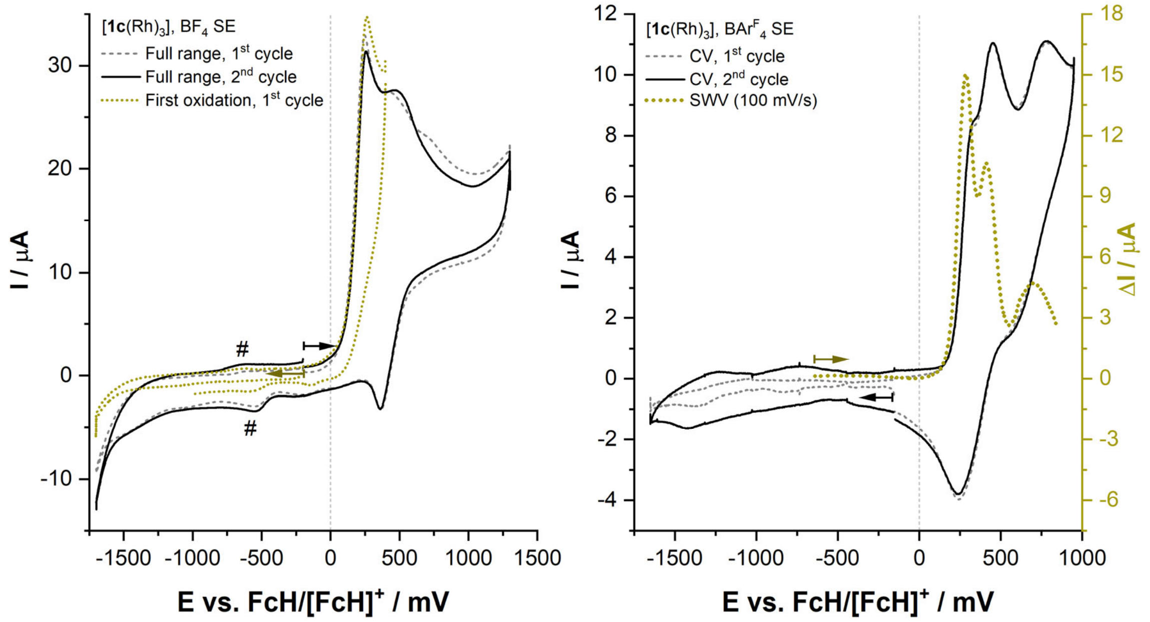

Figure S36. Cyclic voltammograms of C_3N_3 ligand core rhodium complex **[1c(Rh)3]** in BF_4- (left) and BAr^F_4-based (right) supporting electrolytes. The second of three consecutively measured cycles are shown if not indicated otherwise. The scan rate was set to 100 mV·s^{-1}; scan direction and initial potential indicated by the arrow. Cathodic reduction events that are linked to prior oxidation of metal centres are indicated with the hashtag symbol (#). In the case of the BAr^F_4-based supporting electrolyte, application of higher anodic potentials led to the onset of electrolyte decomposition and is thus not shown. Square-wave voltammetry (SWV; y axis on the right) has been run at 100 mV·s^{-1} and is shown to further illustrate the severely broadened third ferrocene-based oxidation and the closely spaced first two oxidation events.

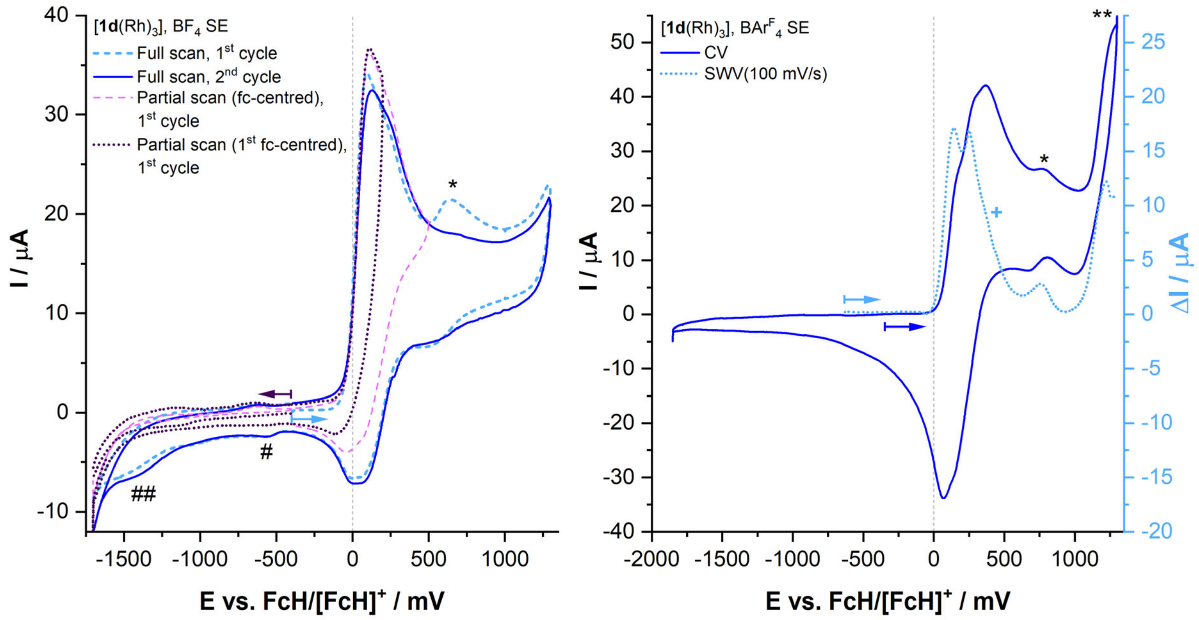

Figure S37. Cyclic voltammograms of $C_6(CH_2)_3$ ligand core rhodium complex **[1d(Rh)3]** in BF_4- (left) and $BAr^F{}_4$-based (right) supporting electrolytes. The second of three consecutively measured cycles are shown if not indicated otherwise. The scan rate was set to 100 mV·s^{-1}; scan direction and initial potential indicated by the arrow. Cathodic reduction events that are linked to prior oxidation of metal centres are indicated with the hashtag symbol (#). In the case of the $BAr^F{}_4$-based supporting electrolyte, application of higher anodic potentials led to the onset of electrolyte decomposition and is thus not shown. Square-wave voltammetry (SWV; y axis on the right) has been run at 100 mV·s^{-1} and is shown to highlight the closely spaced second and third (+) oxidation event of the tris-ferrocenyl core and the second, likely Rh-centred oxidation (**).

4.3. Ruthenium Complexes

BF_4-based Supporting Electrolyte

Table S5. Redox E° and peak potentials $E^{ox/red}$ [mV] of the tris(1-diphenylphosphanyl-1'-ferrocenylene)arene ruthenium(II) complexes **[1a-d(Ru)$_3$]** in 0.1 M (^{n}Bu$_4$N)BF$_4$/CH$_2$Cl$_2$ determined by cyclic voltammetry.[a]

	E^o_{fc} (ΔE_p)	E^{ox}_{Ru} / E^{red}_{Ru} [b]	E^{ox}_{cath} / E^{red}_{cath} [c]
**[1a(Ru)$_3$] (C$_6$H$_3$)**	89 (297)	665, 925 / 626	−404 / −539
**[1b(Ru)$_3$] (C$_6$F$_3$)**	170 (221)	948 / 660	−365 / −522
**[1c(Ru)$_3$] (C$_3$N$_3$)**	243 (176)[d]	929 / -	−353 / −413
**[1d(Ru)$_3$] (C$_6$(CH$_2$)$_3$)**	54 (136)	690, 900, 1223 / 640	−402 / −535

[a] Potentials *vs*. the FcH/[FcH]$^+$ couple at a glassy carbon working electrode (scan rate 100 mV·s^{-1}), the splitting between the anodic (oxidation) and cathodic (reduction) peaks given in brackets where applicable; [b] Peak potentials of the Ru-centred oxidation and reduction events in anhydrous 0.1 mol·L^{-1} (*n*Bu$_4$N)BF$_4$/CH$_2$Cl$_2$ solution; [c] Peak potentials of a second, cathodically shifted reduction and a concomitant oxidation event observed after a first redox cycle of the analyte in anhydrous 0.1 mol·L^{-1} (*n*Bu$_4$N)BF$_4$/CH$_2$Cl$_2$ solution; [d] Not fully reversible at 100 mV·s^{-1} on its own, further losing reversible character upon continued scanning to higher potential

As an electrochemical comparison for **[1a(Ru)$_3$]**, a tethered (1'-methoxy-1-ferrocenylene)-based diarylphosphine ruthenium(II) complex, recently reported by our group, is well suited and shows similar redox properties with $E^0(Fe^{II}/Fe^{III})$ = 110 mV and $E^{ox}(Ru^{II}/Ru^{III})$ = 700 mV *vs*. FcH/[FcH]$^+$ in (*n*Bu$_4$N)PF$_6$/CH$_2$Cl$_2$ [J. Popp, S. Hanf and E. Hey-Hawkins, ACS Omega, **2019**, *4*, 22540]. In general, all ruthenium(II) complexes show a very similar electrochemical fingerprint under the given conditions (Figures S38-S41). In the BF$_4$-based SE (Figure S38), first, a (quasi)-reversible oxidation for the tris(ferrocenyl)arene core takes place, resulting in cathodically shifted reduction events (not directly shown for **[1a(Ru)$_3$]**, for which the cathodic vertex potential for that measurement was chosen differently). At more anodic potential, one or more presumably ruthenium-centred oxidation events occur, resulting in (partial) loss of reversibility for the ligand-core-related redox events, the least prominent for **[1b(Ru)$_3$]** and **[1d(Ru)$_3$].**

BAr^F_4-based Supporting Electrolyte

Table S6. Redox E° and peak potentials $E^{ox/red}$ [mV] of the tris(1-diphenylphosphanyl-1'-ferrocenylene)arene ruthenium(II) complexes **[1a-d(Ru)$_3$]** in 0.1 M (*n*Bu$_4$N)BAr$^F_4$/CH$_2$Cl$_2$ determined by cyclic voltammetry (BAr$^F_4$ = [B{3,5-(CF$_3$)$_2$C$_6$H$_3$}$_4$]; n.r. = not resolved).[a]

	E^o_1 (ΔE_p)	E^o_2 (ΔE_p)	E^o_3 (ΔE_p)	$\Delta E^o_{1/2}$ / $\Delta E^o_{2/3}$ [b]	E^{ox}_{Ru} / E^{red}_{Ru} [c]
**[1a(Ru)$_3$] (C$_6$H$_3$)**	64 (118)	184 (127)	336 (133)	119 / 152	869, 1089 / -
**[1b(Ru)$_3$] (C$_6$F$_3$)**	116 (140)	267 (151)	448 (182)	151 / 181	844, 1178 / 847, 1003
**[1c(Ru)$_3$] (C$_3$N$_3$)**	204 (122)	325 (146)	535 (179)	121 / 210	841, 1124 / −450 [d]
**[1d(Ru)$_3$] (C$_6$(CH$_2$)$_3$)**	91 (148)	134[e] (234)	-	43 / n.r.	843 / -

[a] Potentials *vs*. the FcH/[FcH]$^+$ couple at a glassy carbon working electrode (scan rate 100 mV·s^{-1}), the splitting between the anodic (oxidation) and cathodic (reduction) peaks given in brackets where applicable; [b] Potential-to-potential separation of the first and second/second and third redox process, respectively; [c] Peak potentials of the Ru-centred oxidation and reduction events; [d] Marking a cathodically shifted reduction event that appears on previous oxidation of the Ru^{II} centres; [e] The second and third Fe-centred oxidations were not resolved in cyclic voltammetry, and the second redox event was characterised by a significantly broadened CV wave, likely involving a 2e$^-$ process.

When measured in the BAr$^F_4$-based SE (Figures S38-S41), the well-known threefold redox chemistry of the tris(ferrocenyl)arene core is retained (bold lines). The ruthenium-centred oxidations split into two distinct yet irreversible oxidation events and, for **[1b(Ru)$_3$]** (green) and **[1c(Ru)$_3$]** (blue), seem to consist of one 1e$^-$- and one 2e$^-$-transfer steps.

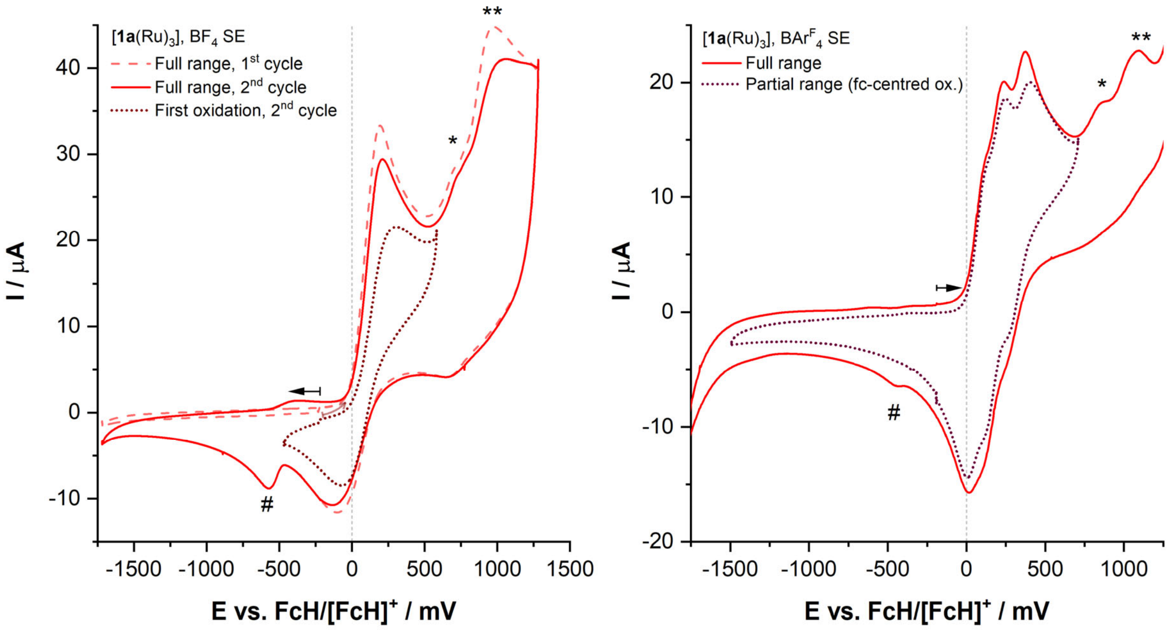

Figure S38. Cyclic voltammograms of C_6H_3 ligand core ruthenium complex **[1a(Ru)$_3$]** in BF_4- (left) and BAr^F_4-based (right) supporting electrolytes. The second of three consecutively measured cycles are shown if not indicated otherwise. The scan rate was set to 100 mV·s^{-1}; scan direction and initial potential indicated by the arrow. Presumably Ru-centred oxidation events are marked with asterisks (*, **); cathodic reduction events that are linked to prior oxidation of metal centres are indicated with the hashtag symbol (#). In the case of the BAr^F_4-based supporting electrolyte, application of higher anodic potentials led to the onset of electrolyte decomposition and is thus not shown.

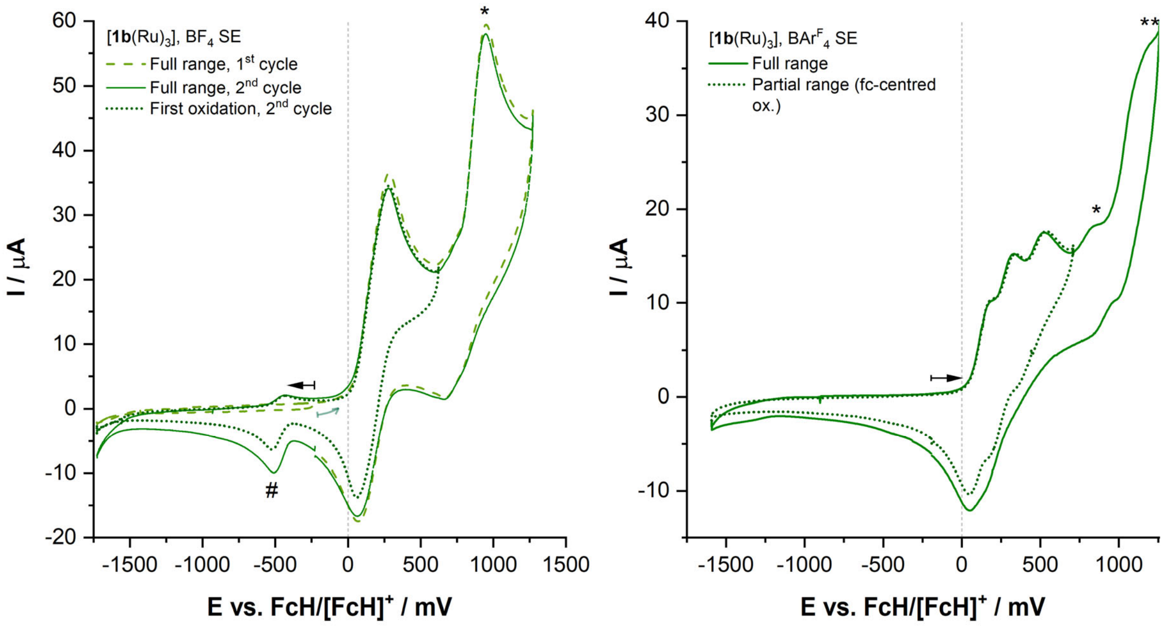

Figure S39. Cyclic voltammograms of C_6F_3 ligand core ruthenium complex **[1b(Ru)$_3$]** in BF_4- (left) and BAr^F_4-based (right) supporting electrolytes. The second of three consecutively measured cycles are shown if not indicated otherwise. The scan rate was set to 100 mV·s^{-1}; scan direction and initial potential indicated by the arrow. Presumably Ru-centred oxidation events are marked with asterisks (*, **); cathodic reduction events that are linked to prior oxidation of metal centres are indicated with the hashtag symbol (#).

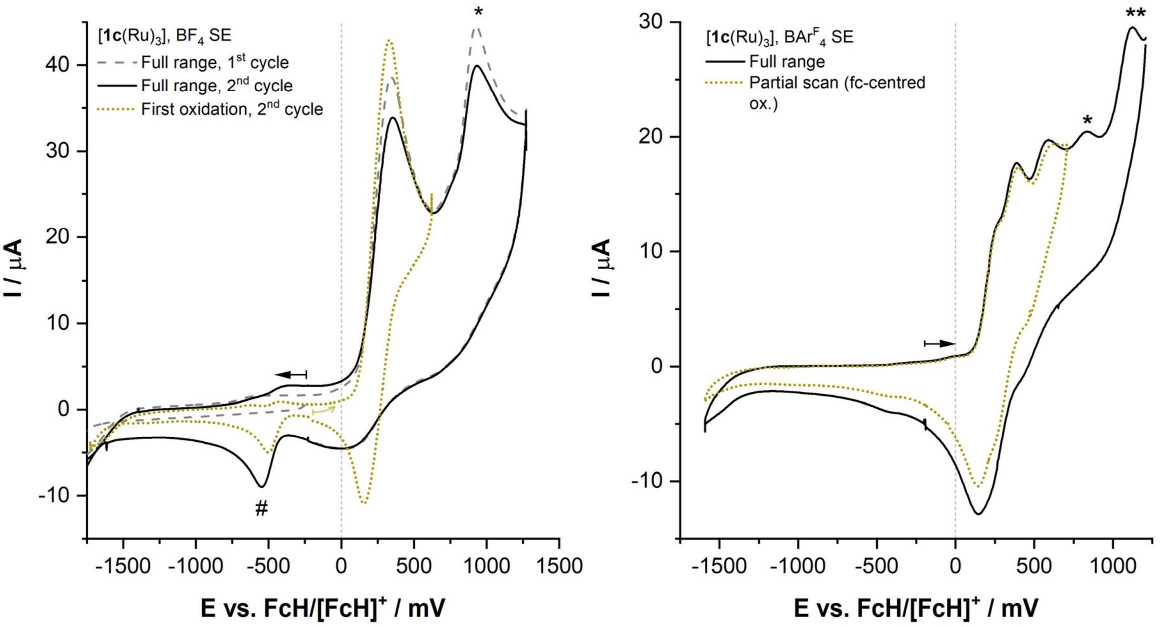

Figure S40. Cyclic voltammograms of C_3N_3 ligand core ruthenium complex **[1c(Ru)$_3$]** in BF_4- (left) and BAr^F_4-based (right) supporting electrolytes. The second of three consecutively measured cycles are shown if not indicated otherwise. The scan rate was set to 100 mV·s^{-1}; scan direction and initial potential indicated by the arrow. Presumably Ru-centred oxidation events are marked with asterisks (*, **); cathodic reduction events that are linked to prior oxidation of metal centres are indicated with the hashtag symbol (#).

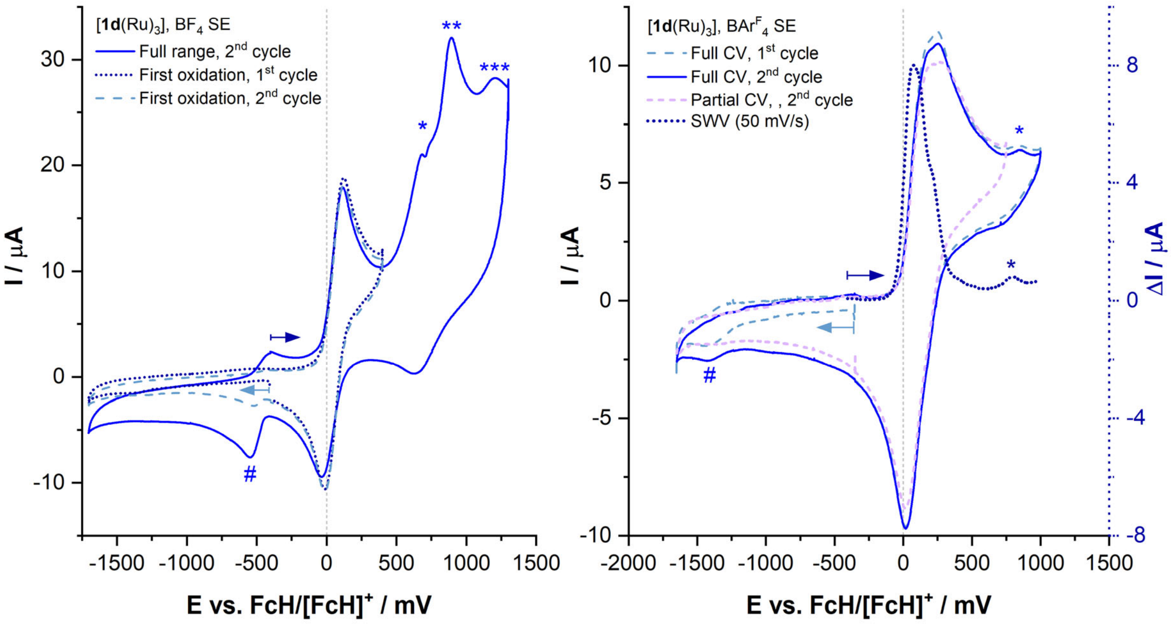

Figure S41. Cyclic voltammograms of $C_6(CH_2)_3$ ligand core ruthenium complex **[1d(Ru)$_3$]** in BF_4- (left) and BAr^F_4-based (right) supporting electrolytes. The scan rate was set to 100 mV·s^{-1}; scan direction and initial potential indicated by the arrow. Presumably Ru-centred oxidation events are marked with asterisks (*–***); cathodic reduction events that are linked to prior oxidation of metal centres are indicated with the hashtag symbol (#). Square-wave voltammetry (SWV; y axis on the right) has been run at 50 mV·s^{-1} and is shown to illustrate the closely-spaced ferrocene-based oxidation events.

Table S7. Redox potentials of the tris(1-diphenylphosphanyl-1'-ferrocenylene)arene rhodium(I) **[1a(Rh)]** and ruthenium(II) complexes **[1(Ru)]** in two different supporting electrolytes determined by cyclic voltammetry.[a]

**Electrolyte**	**(nBu$_4$N)BF$_4$/CH$_2$Cl$_2$**			**(nBu$_4$N)BAr$^F_4$/CH$_2$Cl$_2$**				
**Potentials [mV]**	**$E°_{fc}$ (ΔE_p)**[b]	**E^{ox}_M / E^{red}_M**[c]	**E^{ox}_{fl} / E^{red}_{fl}**[d]	**$E°_1$ (ΔE_p)**[e]	**$E°_2$ (ΔE_p)**[e]	**$E°_3$ (ΔE_p)**[e]	**$\Delta E°_{1/2}$ / $\Delta E°_{2/3}$**[f]	**E^{ox}_M / E^{red}_M**[g]
**[1a(Rh)$_3$]**	112, 260 / 89 [h]	751 / 537	- / −1400	158 (99)	305 (134)	466 (126)	147 / 161	854 / -
**[1a(Ru)$_3$]**	89 (297)	665, 925 / 626	−404 / −539	64 (118)	184 (127)	336 (133)	119 / 152	869, 1089 / -
**[1b(Ru)$_3$]**	170 (221)	948 / 660	−365 / −522	116 (140)	267 (151)	448 (182)	151 / 181	844, 1178 / 847, 1003
**[1c(Ru)$_3$]**	243 (176)[i]	929 / -	−353 / −413	204 (122)	325 (146)	535 (179)	121 / 210	841, 1124 / −450 [j]

[a] Potentials *vs*. the FcH/[FcH]$^+$ couple at a glassy carbon working electrode (scan rate 100 mV·s^{-1}), the splitting between the anodic (oxidation) and cathodic (reduction) peaks given in brackets; [b] Determined in an anhydrous 0.1 mol·L^{-1} (nBu$_4$N)BF$_4$/CH$_2$Cl$_2$ solution; [c] Peak potentials of the Rh/Ru-centred oxidation and reduction events in anhydrous 0.1 mol·L^{-1} (nBu$_4$N)BF$_4$/CH$_2$Cl$_2$ solution; [d] Peak potentials of a second, cathodically shifted reduction and a concomitant oxidation event observed after a first redox cycle of the analyte in anhydrous 0.1 mol·L^{-1} (nBu$_4$N)BF$_4$/CH$_2$Cl$_2$ solution; [e] Determined in an anhydrous 0.1 mol·L^{-1} (nBu$_4$N)BAr$^F_4$/CH$_2$Cl$_2$ (BAr$^F_4$ = [B{3,5-(CF$_3$)$_2$C$_6$H$_3$}$_4$]) solution; [f] Peak-to-peak separation of the first and second/second and third redox process in (nBu$_4$N)BAr$^F_4$/CH$_2$Cl$_2$; [g] Peak potentials of the Rh/Ru-centred oxidation and reduction events in anhydrous 0.1 mol·L^{-1} (nBu$_4$N)BAr$^F_4$/CH$_2$Cl$_2$ solution; [h] The Fe-centred oxidations being non-reversible, the peak potentials for both oxidations and the corresponding reduction events are given; [i] Not fully reversible at 100 mV·s^{-1} on its own, further losing reversible character upon continued scanning to higher potential; [j] Marking a cathodically shifted reduction event that appears on previous oxidation of the Ru^{II} centres.
